# Supplementary material for: From-Toilet-to-Freezer: A Review on Requirements for an Automatic Protocol to Collect and Store Human Fecal Samples for Research Purposes
Source: Biomedicines. 2023 Sep 28;11(10):2658. doi: 10.3390/biomedicines11102658 (PMC10603957; doi:10.3390/biomedicines11102658)
Supplement: Supplementary file 1 [file biomedicines-11-02658-s001.zip › biomedicines-2620778-supplementary.pdf]

Table S1. Examples of works in the literature studying the relation between fecal microbiota and health or disease at phylum, family, genus and species levels.

	Sleep apnea	Type 2 diabetes	Inflammatory bowel disease (IBD)	Irritable bowel syndrome (IBS)	Underweight, malnourished or anorexia	Overweight to obesity level	Colorectal cancer (CRC)	Alzheimer's disease	Autism spectrum disorder	Parkinson's disease	Depression	Hepatic encephalophaty
**Phylum**												
Actinobacteria	[1]	[2]	[3]	[4]	[5]	[6]	[7]	[8]	[9]	[10]	[11]	[12]
Bacteroidetes	[13]	[14]	[15]	[16]	[17]	[18]	[19]	[20]	[21]	[22]	[23]	[24]
Firmicutes	[13]	[25]	[26]	[27]	[28]	[29]	[30]	[31]	[21]	[32]	[33]	[34]
Fusobacteria	[35]	[36]	[37]	[38]	[39]	[40]	[41]	[42]	[43]	[44]	[23]	[45]
Proteobacteria	[13]	[46]	[47]	[48]	[49]	[50]	[51]	[52]	[53]	[54]	[55]	[24]
Spirochaetes	[56]	[57]	[58]	[59]	[60]	[61]	[62]	[63]	[64]	[65]	[66]	[67]
Verrucomicrobia	[68]	[69]	[70]	[71]	[72]	[73]	[74]	[75]	[76]	[54]	[77]	[78]
**Family**												
Akkermansiaceae	[79]	[80]	[81]	[82]	[83]	[84]	[74]	[85]	[86]	[87]	[88]	[89]
Alcaligenaceae	[90]	[91]	[92]	[93]	[94]	[95]	[96]	[97]	[98]	[99]	[100]	[101]
Bacteroidaceae	[102]	[103]	[104]	[105]	[106]	[107]	[108]	[31]	[109]	[110]	[111]	[112]
Bifidobacteriaceae	[113]	[114]	[115]	[116]	[117]	[118]	[119]	[120]	[121]	[122]	[123]	[124]
Clostridiaceae	[13]	[2]	[125]	[126]	[127]	[128]	[129]	[130]	[131]	[44]	[132]	[133]
Coriobacteriaceae	[134]	[135]	[136]	[137]	[138]	[139]	[96]	[140]	[141]	[142]	[132]	[143]
Corynebacteriaceae	[144]	[145]	[146]	[147]	[148]	[149]	[150]	[151]	[53]	[152]	[153]	[154]
Desulfovibrionaceae	[13]	[155]	[92]	[156]	[157]	[158]	[159]	[160]	[161]	[162]	[163]	[89]
Enterobacteriaceae	[164]	[155]	[165]	[166]	[167]	[95]	[168]	[169]	[170]	[171]	[172]	[101]
Enterococcaceae	[173]	[174]	[175]	[156]	[109]	[176]	[177]	[178]	[109]	[171]	[179]	[180]
Erysipelotrichaceae	[181]	[182]	[104]	[183]	[184]	[185]	[186]	[187]	[161]	[188]	[189]	[190]
Eubacteriaceae	[13]	[191]	[192]	[193]	[167]	[194]	[195]	[196]	[43]	[197]	[123]	[101]
Fusobacteriaceae	[198]	[155]	[92]	[199]	[83]	[200]	[201]	[202]	[64]	[203]	[204]	
Geobacteraceae		[205]		[93]		[206]	[207]				[93]	
Helicobacteraceae	[208]	[209]	[210]	[211]	[212]	[213]	[214]	[215]	[216]	[217]	[218]	[89]
Lachnospiraceae	[219]	[220]	[221]	[156]	[222]	[223]	[224]	[8]	[225]	[226]	[88]	[45]
Lactobacillaeceae	[113]	[227]	[228]	[229]	[106]	[230]	[231]	[63]	[232]	[233]	[234]	[45]
Leptotrichiaceae	[235]	[236]	[237]	[147]		[238]	[239]	[240]		[241]	[242]	[243]
Leuconostocaceae	[244]	[245]	[246]	[156]	[247]	[248]	[244]	[249]	[161]	[250]	[251]	[101]
Methanobacteriaceae		[252]	[253]	[254]	[255]	[256]	[257]	[242]		[258]	[259]	[260]
Oscillospiraceae	[13]	[261]	[262]	[193]	[263]	[264]	[265]	[63]	[266]	[267]	[268]	[269]
Peptostreptococcaceae	[164]	[270]	[271]	[156]	[272]	[273]	[274]	[31]	[232]	[275]	[123]	[78]
Porphyromonadaceae		[276]	[277]	[71]	[157]	[278]	[186]	[279]	[280]	[281]	[282]	[283]

Prevotellaceae	[13]	[103]	[284]	[55]	[17]	[285]	[186]	[286]	[287]	[288]	[289]	[290]
Propionibacteriaceae		[155]	[291]	[291]	[292]	[293]	[294]	[242]		[295]	[296]	[297]
Pseudomonadaceae			[298]	[299]	[300]	[301]	[302]	[303]	[161]	[304]	[305]	[306]
Rhodospirillaceae		[307]	[308]	[309]	[310]	[278]	[311]	[312]			[313]	
Rikenellaceae	[314]	[227]	[15]	[315]	[316]	[278]	[214]	[317]	[280]	[318]	[319]	[180]
Ruminococcaceae	[219]	[227]	[277]	[320]	[321]	[322]	[129]	[323]	[287]	[122]	[123]	[45]
Sphingobacteriaceae		[324]	[146]	[325]	[326]	[95]	[327]	[249]	[328]	[329]	[330]	
Staphylococcaceae	[314]	[91]	[331]	[332]	[333]	[334]	[335]	[317]	[336]	[337]	[338]	[339]
Streptococcaceae	[164]	[340]	[298]	[341]	[148]	[342]	[343]	[344]	[345]	[318]	[346]	[347]
Succinivibrionaceae		[348]	[70]	[349]	[350]	[350]	[351]			[329]	[100]	[352]
Sutterellaceae	[13]	[353]	[354]	[156]	[355]	[355]	[356]	[357]	[358]	[329]	[289]	[124]
Tannerellaceae		[359]	[360]			[361]	[201]	[362]		[295]	[363]	[364]
Treponemataceae												
Veillonellaceae	[90]	[365]	[15]	[366]	[17]	[367]	[368]	[369]	[370]	[329]	[371]	[101]
**Genus**												
Akkermansia	[164]	[372]	[373]	[374]	[375]	[376]	[377]	[378]	[379]	[380]	[381]	
Alistipes	[382]	[383]	[360]	[384]	[50]	[385]	[386]	[130]	[21]	[281]	[172]	[78]
Alkaliphilus			[387]	[388]		[389]			[390]			[391]
Atopobium	[392]	[393]	[394]	[395]		[396]	[368]	[42]	[43]	[397]	[11]	[398]
Bacteroides	[13]	[399]	[400]	[401]	[402]	[403]	[177]	[279]	[404]	[258]	[405]	[133]
Barnesiella	[406]	[407]	[408]	[409]	[222]	[410]	[411]	[412]	[161]	[281]	[413]	[414]
Bifidobacterium	[392]	[415]	[416]	[320]	[417]	[418]	[419]	[75]	[358]	[420]	[421]	[422]
Bilophila	[164]	[423]	[424]	[425]	[417]	[426]	[427]	[160]	[21]	[428]	[429]	[414]
Blautia	[13]	[430]	[431]	[71]	[432]	[433]	[434]	[286]	[9]	[435]	[436]	[12]
Butyricicoccus	[437]	[438]	[439]	[440]	[272]	[441]	[214]	[196]	[442]	[443]	[436]	
Caloramator			[444]						[76]			
Catenibacterium		[445]	[92]	[183]	[402]	[446]	[427]	[447]	[448]	[449]	[100]	[450]
Clostridium	[392]	[2]	[451]	[452]	[167]	[453]	[377]	[454]	[455]	[456]	[457]	[422]
Collinsella	[392]	[458]	[459]	[460]	[461]	[462]	[463]	[447]	[21]	[464]	[123]	[465]
Coprococcus	[466]	[467]	[468]	[27]	[469]	[470]	[427]	[412]	[471]	[258]	[472]	[78]
Corynebacterium	[473]	[474]	[475]	[440]	[476]	[477]	[478]	[479]	[21]	[162]	[480]	[481]
Desulfovibrio	[208]	[482]	[92]	[483]	[402]	[484]	[427]	[485]	[486]	[487]	[488]	[489]
Dialister	[13]	[490]	[491]	[492]	[493]	[494]	[495]	[31]	[21]	[496]	[497]	[78]
Dorea	[173]	[498]	[431]	[384]	[499]	[500]	[302]	[501]	[9]	[502]	[503]	[504]
Eggerthella	[505]	[506]	[210]	[507]	[508]	[509]	[510]	[202]	[511]	[512]	[488]	[513]
Enterococcus	[392]	[514]	[515]	[516]	[517]	[518]	[519]	[12]	[345]	[520]	[521]	[522]
Escherichia	[164]	[523]	[524]	[525]	[526]	[527]	[419]	[528]	[529]	[530]	[531]	[532]
Eubacterium	[13]	[533]	[373]	[229]	[316]	[534]	[535]	[536]	[161]	[464]	[472]	[537]
Faecalibacterium	[13]	[538]	[539]	[540]	[316]	[541]	[542]	[543]	[544]	[502]	[545]	[546]
Fusobacterium	[35]	[69]	[547]	[548]	[39]	[549]	[550]	[551]	[64]	[44]	[23]	[532]
Geobacter		[552]							[448]			

Gordonibacter		[135]	[553]	[492]		[554]	[257]	[447]		[555]	[556]	
Helicobacter	[557]	[558]	[559]	[560]	[561]	[562]	[563]	[564]	[565]	[566]	[567]	[568]
Holdemania	[437]	[569]	[570]	[571]	[572]	[293]	[573]		[64]	[574]	[488]	[575]
Klebsiella	[13]	[482]	[576]	[577]	[578]	[579]	[580]	[581]	[582]	[583]	[584]	[465]
Lactobacillus	[392]	[585]	[586]	[460]	[316]	[418]	[377]	[587]	[588]	[258]	[421]	[589]
Leuconostoc	[590]	[61]	[586]	[16]		[554]	[591]	[592]	[593]	[594]	[595]	[532]
Marvinbryantia	[314]	[596]	[81]	[597]	[598]	[230]	[599]	[600]	[601]	[602]	[603]	
Methanobrevibacter	[604]	[605]	[606]	[607]		[418]	[608]	[609]	[610]	[267]	[611]	
Oscillibacter	[392]	[612]	[613]	[614]		[410]	[30]	[615]	[616]	[617]	[23]	[618]
Oscillospira	[619]	[620]	[387]	[621]	[578]	[578]	[622]	[623]	[624]	[258]	[625]	[143]
Parabacteroides	[382]	[626]	[360]	[627]	[526]	[628]	[629]	[630]	[624]	[22]	[631]	[101]
Peptostreptococcus	[392]	[2]	[632]	[507]		[633]	[274]	[634]	[131]	[635]	[123]	[481]
Prevotella	[636]	[637]	[638]	[639]	[272]	[403]	[640]	[641]	[404]	[642]	[584]	[643]
Propionibacterium	[392]	[644]	[291]	[374]	[645]	[646]	[377]	[75]	[161]	[22]		[504]
Pseudomonas	[382]	[445]	[647]	[648]		[649]	[650]	[651]	[652]	[653]	[654]	[12]
Rhodospirillum			[655]			[656]	[657]	[658]		[574]	[659]	
Roseburia	[392]	[14]	[660]	[483]	[661]	[662]	[663]	[75]	[588]	[664]	[665]	[504]
Ruminococcus	[208]	[372]	[666]	[384]	[499]	[667]	[411]	[8]	[668]	[258]	[669]	[78]
Sarcina		[670]	[671]			[672]	[573]		[345]		[673]	
Shigella	[164]	[674]	[277]	[388]	[675]	[509]	[650]	[378]	[21]	[435]	[488]	[12]
Slackia	[164]	[676]	[373]	[27]	[167]	[677]	[678]	[120]		[329]	[679]	
Sneathia		[680]	[681]	[374]		[682]	[463]	[587]				
Sphingobacterium		[683]	[684]	[254]		[95]	[685]	[686]		[687]	[330]	
Sporobacter				[492]		[688]	[689]		[232]		[690]	
Staphylococcus	[382]	[691]	[692]	[507]	[167]	[693]	[311]	[694]	[565]	[695]	[696]	[532]
Streptococcus	[392]	[697]	[698]	[699]	[417]	[534]	[274]	[279]	[700]	[701]	[346]	[45]
Succinivibrio	[702]	[703]	[704]	[705]	[432]	[706]	[351]	[501]		[687]	[100]	
Sutterella	[392]	[423]	[92]	[627]	[707]	[649]	[708]	[317]	[668]	[44]	[123]	[101]
Symbiobacterium			[709]			[710]						
Tannerella	[711]	[712]	[713]	[116]		[714]	[715]	[716]	[717]	[718]	[719]	
Treponema	[720]	[721]	[722]	[349]		[723]	[495]	[724]		[250]	[725]	
Veillonella	[164]	[61]	[726]	[395]		[727]	[728]	[447]	[729]	[329]	[730]	[481]
Xylanibacter		[731]	[732]	[699]		[733]	[734]					
**Species**												
Akkermansia muciniphila	[735]	[736]	[737]	[483]	[72]	[738]	[739]	[740]	[610]	[741]	[742]	
Alistipes finegoldii		[743]	[744]			[745]	[746]	[747]	[748]	[749]	[750]	
Bacteroides caccae	[751]	[752]	[638]	[753]		[754]	[755]		[266]		[405]	
Bacteroides fragilis	[13]	[340]	[475]	[116]	[756]	[757]	[758]	[8]	[759]	[397]	[760]	[761]
Bacteroides uniformis		[762]	[763]		[764]	[549]	[30]	[303]	[765]	[766]	[405]	[618]
Bacteroides vulgatus	[767]	[768]	[769]	[384]	[770]	[771]	[30]	[479]	[759]	[479]	[772]	[618]

Bifidobacterium adolescentis		[773]	[774]	[775]	[402]	[771]	[776]	[75]	[777]	[778]	[779]	[780]
Bifidobacterium bifidum		[781]	[416]	[782]	[499]	[693]	[783]	[784]	[785]	[786]	[421]	
Bifidobacterium catenulatum		[787]	[788]	[789]	[402]	[693]	[790]		[791]	[792]	[793]	
Bifidobacterium longum	[794]	[538]	[795]	[796]	[499]	[771]	[377]	[784]	[797]	[766]	[798]	[799]
Bilophila wadsworthia	[800]	[801]	[802]	[803]	[417]	[804]	[563]	[805]	[806]	[162]	[807]	
Clostridium coccoides		[393]	[475]	[452]	[808]	[809]	[810]	[811]	[455]	[397]	[812]	
Clostridium difficile			[451]	[395]	[813]	[814]	[815]	[811]	[816]	[530]	[817]	[818]
Clostridium histolyticum			[819]	[820]	[402]	[821]	[822]		[455]		[812]	[34]
Clostridium lituseburense			[823]	[820]		[824]			[825]			
Desulfovibrio intestinalis									[826]			
Dialister invisus	[827]	[182]	[769]	[828]		[829]	[830]		[759]		[831]	
Enterococcus faecium	[832]	[490]	[833]	[834]	[402]	[835]		[378]	[836]	[653]	[521]	[837]
Escherichia coli	[838]	[839]	[840]	[841]	[813]	[527]	[842]	[501]	[529]	[843]	[844]	[845]
Eubacterium rectale	[846]	[847]	[823]	[460]	[402]	[848]	[195]	[811]	[849]	[850]	[851]	[12]
Faecalibacterium prausnitzii	[13]	[538]	[852]	[853]	[417]	[848]	[542]	[854]	[797]	[855]	[851]	
Fusobacterium nucleatum	[856]	[857]	[858]	[388]		[168]	[739]	[551]	[859]	[520]	[654]	
Helicobacter pylori	[860]	[558]	[559]	[560]	[861]	[862]	[863]	[564]	[565]	[864]	[567]	[568]
Lactobacillus reuteri	[102]	[865]	[866]	[867]	[417]	[418]	[868]	[869]	[870]	[871]	[872]	[873]
Methanobrevibacter smithii		[874]	[606]	[875]	[876]	[418]	[608]	[651]		[855]		
Parabacteroides distasonis	[102]	[877]	[744]	[878]	[167]	[879]	[880]	[747]	[881]	[295]	[631]	
Roseburia hominis	[13]	[882]	[883]	[884]	[885]	[886]	[887]	[805]	[888]		[889]	
Roseburia intestinalis	[832]	[372]	[890]	[388]	[167]	[662]	[377]		[588]	[891]	[889]	[892]
Ruminococcus bromii		[372]	[893]	[894]		[470]	[755]	[895]	[896]	[435]	[346]	
Ruminococcus faecis			[897]	[898]	[432]	[899]			[64]			
Ruminococcus gnavus	[102]	[626]	[666]	[900]	[499]	[899]	[96]	[196]	[668]	[502]	[901]	[902]
Shigella flexneri		[903]	[904]	[905]	[906]	[907]	[908]	[909]	[700]	[910]		

References

1. Bikov A, Szabo H, Piroska M, Kunos L, Szily M, Ligeti B, et al. Gut Microbiome in Patients with Obstructive Sleep Apnoea. Applied Sciences. 2022;12[4]:2007.

2. Doumatey AP, Adeyemo A, Zhou J, Lei L, Adebamowo SN, Adebamowo C, et al. Gut microbiome profiles are associated with type 2 diabetes in urban Africans. Frontiers in cellular and infection microbiology. 2020:63.

3. Ogilvie LA, Jones BV. Dysbiosis modulates capacity for bile acid modification in the gut microbiomes of patients with inflammatory bowel disease: a mechanism and marker of disease? Gut. 2012;61[11]:1642-3.

4. Hustoft T, Hausken T, Ystad S, Valeur J, Brokstad K, Hatlebakk J, et al. Effects of varying dietary content of fermentable short-chain carbohydrates on symptoms, fecal microenvironment, and cytokine profiles in patients with irritable bowel syndrome. Neurogastroenterol Motil. 2017;29[4]:e12969.

5. Landini L, Dadson P, Gallo F, Honka M-J, Cena H. Microbiota in Anorexia Nervosa–Potential for Treatment. Nutr Res Rev. 2022:1-51.

6. Ruiz A, Cerdó T, Jáuregui R, Pieper DH, Marcos A, Clemente A, et al. One-year calorie restriction impacts gut microbial composition but not its metabolic performance in obese adolescents. Environ Microbiol. 2017;19[4]:1536-51.

7. Zhang Y-K, Zhang Q, Wang Y-L, Zhang W-Y, Hu H-Q, Wu H-Y, et al. A Comparison study of age and colorectal cancer-related gut bacteria. Frontiers in Cellular and Infection Microbiology. 2021;11:606490.

8. Zhuang Z-Q, Shen L-L, Li W-W, Fu X, Zeng F, Gui L, et al. Gut microbiota is altered in patients with Alzheimer's disease. J Alzheimers Dis. 2018;63[4]:1337-46.

9. Ding H, Yi X, Zhang X, Wang H, Liu H, Mou W-W. Imbalance in the gut microbiota of children with autism spectrum disorders. Frontiers in Cellular and Infection Microbiology. 2021;11:572752.

10. Li Z, Lu G, Li Z, Wu B, Luo E, Qiu X, et al. Altered actinobacteria and firmicutes phylum associated epitopes in patients with Parkinson's disease. Front Immunol. 2021;12:632482.

11. Otaka M, Kikuchi-Hayakawa H, Ogura J, Ishikawa H, Yomogida Y, Ota M, et al. Effect of Lacticaseibacillus paracasei strain Shirota on improvement in depressive symptoms, and its association with abundance of Actinobacteria in gut microbiota. Microorganisms. 2021;9[5]:1026.

12. Jia W, Rajani C, Kaddurah-Daouk R, Li H. Expert insights: The potential role of the gut microbiome-bile acid-brain axis in the development and progression of Alzheimer's disease and hepatic encephalopathy. Med Res Rev. 2020;40[4]:1496-507.

13. Valentini F, Evangelisti M, Arpinelli M, Di Nardo G, Borro M, Simmaco M, et al. Gut microbiota composition in children with obstructive sleep apnoea syndrome: a pilot study. Sleep Med. 2020;76:140-7.

14. Larsen N, Vogensen FK, Van Den Berg FW, Nielsen DS, Andreasen AS, Pedersen BK, et al. Gut microbiota in human adults with type 2 diabetes differs from non-diabetic adults. PLoS One. 2010;5[2]:e9085.

15. Knox NC, Forbes JD, Peterson C-L, Van Domselaar G, Bernstein CN. The gut microbiome in inflammatory bowel disease: lessons learned from other immune-mediated inflammatory diseases. Official journal of the American College of Gastroenterology| ACG. 2019;114[7]:1051-70.

16. Duan R, Zhu S, Wang B, Duan L. Alterations of gut microbiota in patients with irritable bowel syndrome based on 16S rRNA-targeted sequencing: a systematic review. Clinical and translational gastroenterology. 2019;10[2].

17. Wan Y, Yuan J, Li J, Li H, Yin K, Wang F, et al. Overweight and underweight status are linked to specific gut microbiota and intestinal tricarboxylic acid cycle intermediates. Clin Nutr. 2020;39[10]:3189-98.

18. Xu P, Li M, Zhang J, Zhang T. Correlation of intestinal microbiota with overweight and obesity in Kazakh school children. BMC Microbiol. 2012;12:1-6.

19. Gagnière J, Raisch J, Veziant J, Barnich N, Bonnet R, Buc E, et al. Gut microbiota imbalance and colorectal cancer. World J Gastroenterol. 2016;22[2]:501-18.

20. Doifode T, Giridharan VV, Generoso JS, Bhatti G, Collodel A, Schulz PE, et al. The impact of the microbiota-gut-brain axis on Alzheimer's disease pathophysiology. Pharmacol Res. 2021;164:105314.

21. Strati F, Cavalieri D, Albanese D, De Felice C, Donati C, Hayek J, et al. New evidences on the altered gut microbiota in autism spectrum disorders. Microbiome. 2017;5:1-11.

22. Lin C-H, Chen C-C, Chiang H-L, Liou J-M, Chang C-M, Lu T-P, et al. Altered gut microbiota and inflammatory cytokine responses in patients with Parkinson's disease. J Neuroinflammation. 2019;16[1]:1-9.

23. Barandouzi ZA, Starkweather AR, Henderson WA, Gyamfi A, Cong XS. Altered composition of gut microbiota in depression: a systematic review. Frontiers in psychiatry. 2020;11:541.

24. Sung CM, Lin Y-f, Chen K-F, Ke H-m, Huang H-Y, Gong Y-N, et al. Predicting clinical outcomes of cirrhosis patients with hepatic encephalopathy from the fecal microbiome. Cell Mol Gastroenterol Hepatol. 2019;8[2]:301-18. e2.

25. James MM, Pal N, Sharma P, Kumawat M, Shubham S, Verma V, et al. Role of butyrogenic Firmicutes in type-2 diabetes. Journal of Diabetes & Metabolic Disorders. 2022;21[2]:1873-82.

26. Matsuoka K, Kanai T, editors. The gut microbiota and inflammatory bowel disease. Semin Immunopathol; 2015: Springer.

27. Kassinen A, Krogius-Kurikka L, Mäkivuokko H, Rinttilä T, Paulin L, Corander J, et al. The fecal microbiota of irritable bowel syndrome patients differs significantly from that of healthy subjects. Gastroenterology. 2007;133[1]:24-33.

28. Koliada A, Syzenko G, Moseiko V, Budovska L, Puchkov K, Perederiy V, et al. Association between body mass index and Firmicutes/Bacteroidetes ratio in an adult Ukrainian population. BMC Microbiol. 2017;17[1]:1-6.

29. Turnbaugh PJ, Ley RE, Mahowald MA, Magrini V, Mardis ER, Gordon JI. An obesity-associated gut microbiome with increased capacity for energy harvest. Nature. 2006;444[7122]:1027-31.

30. Wang T, Cai G, Qiu Y, Fei N, Zhang M, Pang X, et al. Structural segregation of gut microbiota between colorectal cancer patients and healthy volunteers. The ISME journal. 2012;6[2]:320-9.

31. Vogt NM, Kerby RL, Dill-McFarland KA, Harding SJ, Merluzzi AP, Johnson SC, et al. Gut microbiome alterations in Alzheimer’s disease. Sci Rep. 2017;7[1]:13537.

32. Li Y, Li R, Du Y, Xu X, Xue Y, Gao D, et al. Features of gut microbiota in patients with idiopathic Parkinson's disease. Zhonghua yi xue za zhi. 2020;100[13]:1017-22.

33. Huang Y, Shi X, Li Z, Shen Y, Shi X, Wang L, et al. Possible association of Firmicutes in the gut microbiota of patients with major depressive disorder. Neuropsychiatr Dis Treat. 2018:3329-37.

34. Zuo Z, Fan H, Tang XD, Chen YM, Xun LT, Li Y, et al. Effect of different treatments and alcohol addiction on gut microbiota in minimal hepatic encephalopathy patients. Exp Ther Med. 2017;14[5]:4887-95.

35. Li Q, Xu T, Shao C, Gao W, Wang M, Dong Y, et al. Obstructive sleep apnea is related to alterations in fecal microbiome and impaired intestinal barrier function. Sci Rep. 2023;13[1]:778.

36. Wei B, Wang Y, Xiang S, Jiang Y, Chen R, Hu N. Alterations of gut microbiome in patients with type 2 diabetes mellitus who had undergone cholecystectomy. American Journal of Physiology-Endocrinology and Metabolism. 2021;320[1]:E113-E21.

37. Sheehan D, Moran C, Shanahan F. The microbiota in inflammatory bowel disease. J Gastroenterol. 2015;50:495-507.

38. Mei L, Zhou J, Su Y, Mao K, Wu J, Zhu C, et al. Gut microbiota composition and functional prediction in diarrhea-predominant irritable bowel syndrome. BMC Gastroenterol. 2021;21:1-12.

39. Gao X, Zhang M, Xue J, Huang J, Zhuang R, Zhou X, et al. Body mass index differences in the gut microbiota are gender specific. Front Microbiol. 2018;9:1250.

40. Crovesy L, Masterson D, Rosado EL. Profile of the gut microbiota of adults with obesity: a systematic review. Eur J Clin Nutr. 2020;74[9]:1251-62.

41. Vogtmann E, Hua X, Zeller G, Sunagawa S, Voigt AY, Hercog R, et al. Colorectal cancer and the human gut microbiome: reproducibility with whole-genome shotgun sequencing. PLoS One. 2016;11[5]:e0155362.

42. Bello-Medina PC, Hernández-Quiroz F, Pérez-Morales M, González-Franco DA, Cruz-Pauseno G, García-Mena J, et al. Spatial memory and gut microbiota alterations are already present in early adulthood in a pre-clinical transgenic model of Alzheimer’s disease. Front Neurosci. 2021;15:595583.

43. Ho LKH, Tong VJW, Syn N, Nagarajan N, Tham EH, Tay SK, et al. Gut microbiota changes in children with autism spectrum disorder: a systematic review. Gut Pathog. 2020;12[1]:1-18.

44. Lin A, Zheng W, He Y, Tang W, Wei X, He R, et al. Gut microbiota in patients with Parkinson's disease in southern China. Parkinsonism Relat Disord. 2018;53:82-8.

45. Rai R, Saraswat VA, Dhiman RK. Gut microbiota: its role in hepatic encephalopathy. J Clin Exp Hepatol. 2015;5:S29-S36.

46. Zhang Z, Tian T, Chen Z, Liu L, Luo T, Dai J. Characteristics of the gut microbiome in patients with prediabetes and type 2 diabetes. PeerJ. 2021;9:e10952.

47. Shin N-R, Whon TW, Bae J-W. Proteobacteria: microbial signature of dysbiosis in gut microbiota. Trends Biotechnol. 2015;33[9]:496-503.

48. Barandouzi ZA, Lee J, del Carmen Rosas M, Chen J, Henderson WA, Starkweather AR, et al. Associations of neurotransmitters and the gut microbiome with emotional distress in mixed type of irritable bowel syndrome. Sci Rep. 2022;12[1]:1648.

49. Huey SL, Jiang L, Fedarko MW, McDonald D, Martino C, Ali F, et al. Nutrition and the gut microbiota in 10-to 18-month-old children living in urban slums of Mumbai, India. Msphere. 2020;5[5]:e00731-20.

50. Abdullah B, Daud S, Aazmi MS, Idorus MY, Mahamooth MIJ. Gut microbiota in pregnant Malaysian women: a comparison between trimesters, body mass index and gestational diabetes status. BMC Pregnancy Childbirth. 2022;22[1]:1-15.

51. Liu W, Zhang R, Shu R, Yu J, Li H, Long H, et al. Study of the relationship between microbiome and colorectal cancer susceptibility using 16SrRNA sequencing. BioMed research international. 2020;2020.

52. Franceschi F, Ojetti V, Candelli M, Covino M, Cardone S, Potenza A, et al. Microbes and Alzheimer’disease: lessons from H. pylori and GUT microbiota. Eur Rev Med Pharmacol Sci. 2019;23[1]:426-30.

53. Plaza-Díaz J, Gómez-Fernández A, Chueca N, Torre-Aguilar MJdl, Gil Á, Perez-Navero JL, et al. Autism spectrum disorder [ASD] with and without mental regression is associated with changes in the fecal microbiota. Nutrients. 2019;11[2]:337.

54. Zapała B, Stefura T, Wójcik-Pędziwiatr M, Kabut R, Bałajewicz-Nowak M, Milewicz T, et al. Differences in the composition of gut microbiota between patients with parkinson's disease and healthy controls: A cohort study. Journal of Clinical Medicine. 2021;10[23]:5698.

55. Simpson CA, Mu A, Haslam N, Schwartz OS, Simmons JG. Feeling down? A systematic review of the gut microbiota in anxiety/depression and irritable bowel syndrome. J Affect Disord. 2020;266:429-46.

56. Galli J, Calò L, Posteraro B, Rossi G, Sterbini FP, Paludetti G, et al. Pediatric oropharyngeal microbiome: mapping in chronic tonsillitis and tonsillar hypertrophy. Int J Pediatr Otorhinolaryngol. 2020;139:110478.

57. Zhang F, Wang M, Yang J, Xu Q, Liang C, Chen B, et al. Response of gut microbiota in type 2 diabetes to hypoglycemic agents. Endocrine. 2019;66:485-93.

58. Damman CJ, Miller SI, Surawicz CM, Zisman TL. The microbiome and inflammatory bowel disease: is there a therapeutic role for fecal microbiota transplantation? Official journal of the American College of Gastroenterology| ACG. 2012;107[10]:1452-9.

59. Pimentel M, Lembo A. Microbiome and its role in irritable bowel syndrome. Dig Dis Sci. 2020;65:829-39.

60. Ghosh TS, Sen Gupta S, Bhattacharya T, Yadav D, Barik A, Chowdhury A, et al. Gut microbiomes of Indian children of varying nutritional status. PLoS One. 2014;9[4]:e95547.

61. Graessler J, Qin Y, Zhong H, Zhang J, Licinio J, Wong M-L, et al. Metagenomic sequencing of the human gut microbiome before and after bariatric surgery in obese patients with type 2 diabetes: correlation with inflammatory and metabolic parameters. The pharmacogenomics journal. 2013;13[6]:514-22.

62. Yang Y, Cai Q, Shu XO, Steinwandel MD, Blot WJ, Zheng W, et al. Prospective study of oral microbiome and colorectal cancer risk in low-income and African American populations. Int J Cancer. 2019;144[10]:2381-9.

63. Kaiyrlykyzy A, Kozhakhmetov S, Babenko D, Zholdasbekova G, Alzhanova D, Olzhayev F, et al. Study of gut microbiota alterations in Alzheimer's dementia patients from Kazakhstan. Sci Rep. 2022;12[1]:15115.

64. Zhang Q, Zou R, Guo M, Duan M, Li Q, Zheng H. Comparison of gut microbiota between adults with autism spectrum disorder and obese adults. PeerJ. 2021;9:e10946.

65. Lubomski M, Xu X, Holmes AJ, Muller S, Yang JY, Davis RL, et al. The gut microbiome in Parkinson's disease: A longitudinal study of the impacts on disease progression and the use of device-assisted therapies. Front Aging Neurosci. 2022:414.

66. Paudel D, Uehara O, Giri S, Yoshida K, Morikawa T, Kitagawa T, et al. Effect of psychological stress on the oral-gut microbiota and the potential oral-gut-brain axis. Jpn Dent Sci Rev. 2022;58:365-75.

67. Ling Z, Liu X, Cheng Y, Jiang X, Jiang H, Wang Y, et al. Decreased diversity of the oral microbiota of patients with hepatitis B virus-induced chronic liver disease: a pilot project. Sci Rep. 2015;5[1]:1-11.

68. Anderson JR, Carroll I, Azcarate-Peril MA, Rochette AD, Heinberg LJ, Peat C, et al. A preliminary examination of gut microbiota, sleep, and cognitive flexibility in healthy older adults. Sleep Med. 2017;38:104-7.

69. Salguero MV, Al-Obaide MA, Singh R, Siepmann T, Vasylyeva TL. Dysbiosis of Gram-negative gut microbiota and the associated serum lipopolysaccharide exacerbates inflammation in type 2 diabetic patients with chronic kidney disease. Exp Ther Med. 2019;18[5]:3461-9.

70. Salem F, Kindt N, Marchesi JR, Netter P, Lopez A, Kokten T, et al. Gut microbiome in chronic rheumatic and inflammatory bowel diseases: Similarities and differences. United European gastroenterology journal. 2019;7[8]:1008-32.

71. Barandouzi ZA, Lee J, Maas K, Starkweather AR, Cong XS. Altered gut microbiota in irritable bowel syndrome and its association with food components. Journal of Personalized Medicine. 2021;11[1]:35.

72. de Clercq NC, Frissen MN, Davids M, Groen AK, Nieuwdorp M. Weight gain after fecal microbiota transplantation in a patient with recurrent underweight following clinical recovery from anorexia nervosa. Psychother Psychosom. 2019;88[1]:58-60.

73. Barlow GM, Yu A, Mathur R. Role of the gut microbiome in obesity and diabetes mellitus. Nutr Clin Pract. 2015;30[6]:787-97.

74. Huang R, He K, Duan X, Xiao J, Wang H, Xiang G. Changes of intestinal microflora in colorectal cancer patients after surgical resection and chemotherapy. Comput Math Methods Med. 2022;2022.

75. Askarova S, Umbayev B, Masoud A-R, Kaiyrlykyzy A, Safarova Y, Tsoy A, et al. The links between the gut microbiome, aging, modern lifestyle and Alzheimer's disease. Frontiers in cellular and infection microbiology. 2020;10:104.

76. De Angelis M, Piccolo M, Vannini L, Siragusa S, De Giacomo A, Serrazzanetti DI, et al. Fecal microbiota and metabolome of children with autism and pervasive developmental disorder not otherwise specified. PLoS One. 2013;8[10]:e76993.

77. Hemmings SM, Malan-Muller S, van den Heuvel LL, Demmitt BA, Stanislawski MA, Smith DG, et al. The microbiome in posttraumatic stress disorder and trauma-exposed controls: an exploratory study. Psychosom Med. 2017;79[8]:936.

78. Li M, Li K, Tang S, Lv Y, Wang Q, Wang Z, et al. Restoration of the gut microbiota is associated with a decreased risk of hepatic encephalopathy after TIPS. JHEP Reports. 2022;4[5]:100448.

79. Nogal A, Valdes AM, Menni C. The role of short-chain fatty acids in the interplay between gut microbiota and diet in cardio-metabolic health. Gut microbes. 2021;13[1]:1897212.

80. Deledda A, Palmas V, Heidrich V, Fosci M, Lombardo M, Cambarau G, et al. Dynamics of Gut Microbiota and Clinical Variables after Ketogenic and Mediterranean Diets in Drug-Naïve Patients with Type 2 Diabetes Mellitus and Obesity. Metabolites. 2022;12[11]:1092.

81. Chang C-C, Liu C-Y, Su I-C, Lee Y-J, Yeh H-J, Chen W-C, et al. Functional Plasmon-Activated Water Increases Akkermansia muciniphila Abundance in Gut Microbiota to Ameliorate Inflammatory Bowel Disease. Int J Mol Sci. 2022;23[19]:11422.

82. Wang Q-S, Wang Y-L, Zhang W-Y, Li K-D, Luo X-F, Cui Y-L. Puerarin from Pueraria lobata alleviates the symptoms of irritable bowel syndrome-diarrhea. Food Funct. 2021;12[5]:2211-24.

83. Han D-S, Wu W-K, Liu P-Y, Yang Y-T, Hsu H-C, Kuo C-H, et al. Differences in the gut microbiome and reduced fecal butyrate in elders with low skeletal muscle mass. Clin Nutr. 2022;41[7]:1491-500.

84. Zhang M, Chen J, Yang M, Qian C, Liu Y, Qi Y, et al. Low Doses of Sucralose Alter Fecal Microbiota in High-Fat Diet-Induced Obese Rats. Frontiers in Nutrition. 2021;8:1151.

85. Ubeda C, Vázquez-Carretero MD, Luque-Tirado A, Ríos-Reina R, Rubio-Sánchez R, Franco-Macías E, et al. Fecal Volatile Organic Compounds and Microbiota Associated with the Progression of Cognitive Impairment in Alzheimer's Disease. Int J Mol Sci. 2022;24[1]:707.

86. Li N, Chen H, Cheng Y, Xu F, Ruan G, Ying S, et al. Fecal microbiota transplantation relieves gastrointestinal and autism symptoms by improving the gut microbiota in an open-label study. Frontiers in Cellular and Infection Microbiology. 2021;11:948.

87. Nishiwaki H, Hamaguchi T, Ito M, Ishida T, Maeda T, Kashihara K, et al. Short-chain fatty acid-producing gut microbiota is decreased in Parkinson's disease but not in rapid-eye-movement sleep behavior disorder. Msystems. 2020;5[6]:e00797-20.

88. Park M, Choi J, Lee H-J. Flavonoid-rich orange juice intake and altered gut microbiome in young adults with depressive symptom: a randomized controlled study. Nutrients. 2020;12[6]:1815.

89. Wang L, Wan Y-JY. The role of gut microbiota in liver disease development and treatment. Liver research. 2019;3[1]:3-18.

90. Xu H, Li X, Zheng X, Xia Y, Fu Y, Li X, et al. Pediatric obstructive sleep apnea is associated with changes in the oral microbiome and urinary metabolomics profile: a pilot study. J Clin Sleep Med. 2018;14[9]:1559-67.

91. Bailey MJ, Naik NN, Wild LE, Patterson WB, Alderete TL. Exposure to air pollutants and the gut microbiota: a potential link between exposure, obesity, and type 2 diabetes. Gut Microbes. 2020;11[5]:1188-202.

92. Humbel F, Rieder JH, Franc Y, Juillerat P, Scharl M, Misselwitz B, et al. Association of alterations in intestinal microbiota with impaired psychological function in patients with inflammatory bowel diseases in remission. Clin Gastroenterol Hepatol. 2020;18[9]:2019-29. e11.

93. Jalanka J, Lam C, Bennett A, Hartikainen A, Crispie F, Finnegan LA, et al. Colonic gene expression and fecal microbiota in diarrhea-predominant irritable bowel syndrome: increased toll-like receptor 4 but minimal inflammation and no response to mesalazine. J Neurogastroenterol Motil. 2021;27[2]:279.

94. Eggers S, Safdar N, Sethi AK, Suen G, Peppard PE, Kates AE, et al. Urinary lead concentration and composition of the adult gut microbiota in a cross-sectional population-based sample. Environ Int. 2019;133:105122.

95. Palmas V, Pisanu S, Madau V, Casula E, Deledda A, Cusano R, et al. Gut microbiota markers associated with obesity and overweight in Italian adults. Sci Rep. 2021;11[1]:1-14.

96. Chen W, Liu F, Ling Z, Tong X, Xiang C. Human intestinal lumen and mucosa-associated microbiota in patients with colorectal cancer. PLoS One. 2012;7[6]:e39743.

97. Kobayashi Y, Sugahara H, Shimada K, Mitsuyama E, Kuhara T, Yasuoka A, et al. Therapeutic potential of Bifidobacterium breve strain A1 for preventing cognitive impairment in Alzheimer's disease. Sci Rep. 2017;7[1]:1-10.

98. Williams BL, Hornig M, Parekh T, Lipkin WI. Application of novel PCR-based methods for detection, quantitation, and phylogenetic characterization of Sutterella species in intestinal biopsy samples from children with autism and gastrointestinal disturbances. MBio. 2012;3[1]:e00261-11.

99. Johnson ME, Stringer A, Bobrovskaya L. Rotenone induces gastrointestinal pathology and microbiota alterations in a rat model of Parkinson's disease. Neurotoxicology. 2018;65:174-85.

100. Zheng S, Zhu Y, Wu W, Zhang Q, Wang Y, Wang Z, et al. A correlation study of intestinal microflora and first-episode depression in Chinese patients and healthy volunteers. Brain and Behavior. 2021;11[8]:e02036.

101. Bajaj JS, Ridlon JM, Hylemon PB, Thacker LR, Heuman DM, Smith S, et al. Linkage of gut microbiome with cognition in hepatic encephalopathy. American Journal of Physiology-Gastrointestinal and Liver Physiology. 2012;302[1]:G168-G75.

102. Bikov A, Dragonieri S, Csoma B, Mazzuca C, Finamore P, Rocchi G, et al. The Role of Gut Bacteriome in Asthma, Chronic Obstructive Pulmonary Disease and Obstructive Sleep Apnoea. Microorganisms. 2022;10[12]:2457.

103. Fugmann M, Breier M, Rottenkolber M, Banning F, Ferrari U, Sacco V, et al. The stool microbiota of insulin resistant women with recent gestational diabetes, a high risk group for type 2 diabetes. Sci Rep. 2015;5[1]:1-8.

104. Alam MT, Amos GC, Murphy AR, Murch S, Wellington EM, Arasaradnam RP. Microbial imbalance in inflammatory bowel disease patients at different taxonomic levels. Gut Pathog. 2020;12[1]:1-8.

105. Peter J, Fournier C, Durdevic M, Knoblich L, Keip B, Dejaco C, et al. A microbial signature of psychological distress in irritable bowel syndrome. Psychosom Med. 2018;80[8]:698.

106. Aguilar T, Nava GM, Olvera-Ramírez AM, Ronquillo D, Camacho M, Zavala GA, et al. Gut bacterial families are associated with body composition and metabolic risk markers in school-aged children in rural Mexico. Childhood Obesity. 2020;16[5]:358-66.

107. Cho KY. Lifestyle modifications result in alterations in the gut microbiota in obese children. BMC Microbiol. 2021;21[1]:1-15.

108. Lauka L, Reitano E, Carra MC, Gaiani F, Gavriilidis P, Brunetti F, et al. Role of the intestinal microbiome in colorectal cancer surgery outcomes. World J Surg Oncol. 2019;17[1]:1-12.

109. Pulikkan J, Maji A, Dhakan DB, Saxena R, Mohan B, Anto MM, et al. Gut microbial dysbiosis in Indian children with autism spectrum disorders. Microb Ecol. 2018;76:1102-14.

110. Vidal-Martinez G, Chin B, Camarillo C, Herrera GV, Yang B, Sarosiek I, et al. A pilot microbiota study in Parkinson’s disease patients versus control subjects, and effects of FTY720 and FTY720-mitoxy therapies in parkinsonian and multiple system atrophy mouse models. J Parkinsons Dis. 2020;10[1]:185-92.

111. Zheng P, Yang J, Li Y, Wu J, Liang W, Yin B, et al. Gut microbial signatures can discriminate unipolar from bipolar depression. Advanced Science. 2020;7[7]:1902862.

112. Bajaj JS, Heuman DM, Sanyal AJ, Hylemon PB, Sterling RK, Stravitz RT, et al. Modulation of the metabiome by rifaximin in patients with cirrhosis and minimal hepatic encephalopathy. PLoS One. 2013;8[4]:e60042.

113. Farré N, Farré R, Gozal D. Sleep apnea morbidity: a consequence of microbial-immune cross-talk? Chest. 2018;154[4]:754-9.

114. Gonai M, Shigehisa A, Kigawa I, Kurasaki K, Chonan O, Matsuki T, et al. Galacto-oligosaccharides ameliorate dysbiotic Bifidobacteriaceae decline in Japanese patients with type 2 diabetes. Beneficial microbes. 2017;8[5]:705-16.

115. Teofani A, Marafini I, Laudisi F, Pietrucci D, Salvatori S, Unida V, et al. Intestinal taxa abundance and diversity in inflammatory bowel disease patients: An analysis including covariates and confounders. Nutrients. 2022;14[2]:260.

116. Pittayanon R, Lau JT, Yuan Y, Leontiadis GI, Tse F, Surette M, et al. Gut microbiota in patients with irritable bowel syndrome—a systematic review. Gastroenterology. 2019;157[1]:97-108.

117. Nakayama J, Watanabe K, Jiang J, Matsuda K, Chao S-H, Haryono P, et al. Diversity in gut bacterial community of school-age children in Asia. Sci Rep. 2015;5[1]:8397.

118. Lane M, Howland G, West M, Hockey M, Marx W, Loughman A, et al. The effect of ultra-processed very low-energy diets on gut microbiota and metabolic outcomes in individuals with obesity: a systematic literature review. Obes Res Clin Pract. 2020;14[3]:197-204.

119. Youssef O, Lahti L, Kokkola A, Karla T, Tikkanen M, Ehsan H, et al. Stool microbiota composition differs in patients with stomach, colon, and rectal neoplasms. Dig Dis Sci. 2018;63:2950-8.

120. Nagpal R, Neth BJ, Wang S, Craft S, Yadav H. Modified Mediterranean-ketogenic diet modulates gut microbiome and short-chain fatty acids in association with Alzheimer's disease markers in subjects with mild cognitive impairment. EBioMedicine. 2019;47:529-42.

121. Xie X, Li L, Wu X, Hou F, Chen Y, Shi L, et al. Alteration of the fecal microbiota in Chinese children with autism spectrum disorder. Autism Res. 2022;15[6]:996-1007.

122. Shen T, Yue Y, He T, Huang C, Qu B, Lv W, et al. The association between the gut microbiota and Parkinson's disease, a meta-analysis. Front Aging Neurosci. 2021;13:636545.

123. Knuesel T, Mohajeri MH. The role of the gut microbiota in the development and progression of major depressive and bipolar disorder. Nutrients. 2022;14[1]:37.

124. Bajaj JS, Salzman NH, Acharya C, Sterling RK, White MB, Gavis EA, et al. Fecal microbial transplant capsules are safe in hepatic encephalopathy: a phase 1, randomized, placebo-controlled trial. Hepatology. 2019;70[5]:1690-703.

125. Muñiz Pedrogo DA, Chen J, Hillmann B, Jeraldo P, Al-Ghalith G, Taneja V, et al. An increased abundance of Clostridiaceae characterizes arthritis in inflammatory bowel disease and rheumatoid arthritis: a cross-sectional study. Inflamm Bowel Dis. 2019;25[5]:902-13.

126. Ricci C, Rizzello F, Valerii MC, Spisni E, Gionchetti P, Turroni S, et al. Geraniol treatment for irritable bowel syndrome: A double-blind randomized clinical trial. Nutrients. 2022;14[19]:4208.

127. Hourigan S, Grigoryan Z, Kim S, Chirumamilla S, Rabizadeh S, Golub J, et al. Decreased Diversity of the Fecal Microbiome in Pediatric Carriage of Clostridium difficile. Gastroenterology. 2014;146[5]:S-13.

128. Golloso-Gubat MJ, Ducarmon QR, Tan RCA, Zwittink RD, Kuijper EJ, Nacis JS, et al. Gut microbiota and dietary intake of normal-weight and overweight Filipino children. Microorganisms. 2020;8[7]:1015.

129. Peters BA, Dominianni C, Shapiro JA, Church TR, Wu J, Miller G, et al. The gut microbiota in conventional and serrated precursors of colorectal cancer. Microbiome. 2016;4:1-14.

130. Hung C-C, Chang C-C, Huang C-W, Nouchi R, Cheng C-H. Gut microbiota in patients with Alzheimer's disease spectrum: A systematic review and meta-analysis. Aging [Albany N Y]. 2022;14[1]:477.

131. Srikantha P, Mohajeri MH. The possible role of the microbiota-gut-brain-axis in autism spectrum disorder. Int J Mol Sci. 2019;20[9]:2115.

132. Painold A, Mörkl S, Kashofer K, Halwachs B, Dalkner N, Bengesser S, et al. A step ahead: exploring the gut microbiota in inpatients with bipolar disorder during a depressive episode. Bipolar disorders. 2019;21[1]:40-9.

133. Haraguchi M, Miuma S, Masumoto H, Ichikawa T, Kanda Y, Sasaki R, et al. Bacteroides in colonic mucosa-associated microbiota affects the development of minimal hepatic encephalopathy in patients with cirrhosis. Hepatol Int. 2019;13:482-9.

134. Durgan DJ, Ganesh BP, Cope JL, Ajami NJ, Phillips SC, Petrosino JF, et al. Role of the gut microbiome in obstructive sleep apnea–induced hypertension. Hypertension. 2016;67[2]:469-74.

135. Liu H, Zhang H, Wang X, Yu X, Hu C, Zhang X. The family Coriobacteriaceae is a potential contributor to the beneficial effects of Roux-en-Y gastric bypass on type 2 diabetes. Surg Obes Relat Dis. 2018;14[5]:584-93.

136. Abecia L, Hoyles L, Khoo C, Frantz N, McCartney AL. Effects of a novel galactooligosaccharide on the faecal microbiota of healthy and inflammatory bowel disease cats during a randomized, double-blind, cross-over feeding study. International Journal of Probiotics and Prebiotics. 2010;5[2]:61-8.

137. Krogius-Kurikka L, Lyra A, Malinen E, Aarnikunnas J, Tuimala J, Paulin L, et al. Microbial community analysis reveals high level phylogenetic alterations in the overall gastrointestinal microbiota of diarrhoea-predominant irritable bowel syndrome sufferers. BMC Gastroenterol. 2009;9[1]:1-11.

138. Mörkl S, Lackner S, Müller W, Gorkiewicz G, Kashofer K, Oberascher A, et al. Gut microbiota and body composition in anorexia nervosa inpatients in comparison to athletes, overweight, obese, and normal weight controls. Int J Eat Disord. 2017;50[12]:1421-31.

139. Alderete TL, Jones RB, Chen Z, Kim JS, Habre R, Lurmann F, et al. Exposure to traffic-related air pollution and the composition of the gut microbiota in overweight and obese adolescents. Environ Res. 2018;161:472-8.

140. Wang M, Cao J, Gong C, Amakye WK, Yao M, Ren J. Exploring the microbiota-Alzheimer's disease linkage using short-term antibiotic treatment followed by fecal microbiota transplantation. Brain Behav Immun. 2021;96:227-38.

141. Chen K, Fu Y, Wang Y, Liao L, Xu H, Zhang A, et al. Therapeutic effects of the in vitro cultured human gut microbiota as transplants on altering gut microbiota and improving symptoms associated with autism spectrum disorder. Microb Ecol. 2020;80:475-86.

142. Zhang L-N, Yuan W-L, Ye M, Yin L, Wang S-J. Changes in the intestinal microbiota of patients with Parkinson's disease and their clinical significance. Int J Clin Pharmacol Ther. 2022.

143. Lin Y, Yan G, Feng F, Wang M, Long F. Characterization of intestinal microbiota and serum metabolites in patients with mild hepatic encephalopathy. Open Life Sciences. 2022;17[1]:139-54.

144. Hu C, Wang P, Yang Y, Li J, Jiao X, Yu H, et al. Chronic intermittent hypoxia participates in the pathogenesis of atherosclerosis and perturbs the formation of intestinal microbiota. Frontiers in Cellular and Infection Microbiology. 2021;11:560201.

145. Zhao Z, Chen Y, Li X, Zhu L, Wang X, Li L, et al. Myricetin relieves the symptoms of type 2 diabetes mice and regulates intestinal microflora. Biomed Pharmacother. 2022;153:113530.

146. Regner EH, Ohri N, Stahly A, Gerich ME, Fennimore BP, Ir D, et al. Functional intraepithelial lymphocyte changes in inflammatory bowel disease and spondyloarthritis have disease specific correlations with intestinal microbiota. Arthritis Res Ther. 2018;20:1-13.

147. Ng SC, Lam EF, Lam TT, Chan Y, Law W, Tse PC, et al. Effect of probiotic bacteria on the intestinal microbiota in irritable bowel syndrome. J Gastroenterol Hepatol. 2013;28[10]:1624-31.

148. Brandwein M, Katz I, Katz A, Kohen R. Beyond the gut: skin microbiome compositional changes are associated with BMI. Human Microbiome Journal. 2019;13:100063.

149. Palomo-Buitrago ME, Sabater-Masdeu M, Moreno-Navarrete JM, Caballano-Infantes E, Arnoriaga-Rodríguez M, Coll C, et al. Glutamate interactions with obesity, insulin resistance, cognition and gut microbiota composition. Acta Diabetol. 2019;56:569-79.

150. Tang Q, Liu R, Chu G, Wang Y, Cui H, Zhang T, et al. A Comprehensive Analysis of Microflora and Metabolites in the Development of Ulcerative Colitis into Colorectal Cancer Based on the Lung–Gut Correlation Theory. Molecules. 2022;27[18]:5838.

151. Ling Z, Zhu M, Yan X, Cheng Y, Shao L, Liu X, et al. Structural and functional dysbiosis of fecal microbiota in Chinese patients with Alzheimer's disease. Frontiers in Cell and Developmental Biology. 2021;8:634069.

152. Wallen ZD, Appah M, Dean MN, Sesler CL, Factor SA, Molho E, et al. Characterizing dysbiosis of gut microbiome in PD: evidence for overabundance of opportunistic pathogens. npj Parkinson's Disease. 2020;6[1]:11.

153. Chen Y-h, Xue F, Yu S-f, Li X-s, Liu L, Jia Y-y, et al. Gut microbiota dysbiosis in depressed women: The association of symptom severity and microbiota function. J Affect Disord. 2021;282:391-400.

154. Yan R, Wang K, Wang Q, Jiang H, Lu Y, Chen X, et al. Probiotic Lactobacillus casei Shirota prevents acute liver injury by reshaping the gut microbiota to alleviate excessive inflammation and metabolic disorders. Microb Biotechnol. 2022;15[1]:247-61.

155. Wang Y, Luo X, Mao X, Tao Y, Ran X, Zhao H, et al. Gut microbiome analysis of type 2 diabetic patients from the Chinese minority ethnic groups the Uygurs and Kazaks. PLoS One. 2017;12[3]:e0172774.

156. Lee SM, Kim N, Yoon H, Kim YS, Choi SI, Park JH, et al. Compositional and functional changes in the gut microbiota in irritable bowel syndrome patients. Gut and Liver. 2021;15[2]:253.

157. Lv Y, Qin X, Jia H, Chen S, Sun W, Wang X. The association between gut microbiota composition and BMI in Chinese male college students, as analysed by next-generation sequencing. Br J Nutr. 2019;122[9]:986-95.

158. Xu P, Wang J, Hong F, Wang S, Jin X, Xue T, et al. Melatonin prevents obesity through modulation of gut microbiota in mice. J Pineal Res. 2017;62[4]:e12399.

159. Luo J, Zhang C, Liu R, Gao L, Ou S, Liu L, et al. Ganoderma lucidum polysaccharide alleviating colorectal cancer by alteration of special gut bacteria and regulation of gene expression of colonic epithelial cells. J Funct Foods. 2018;47:127-35.

160. Sheng C, Yang K, He B, Du W, Cai Y, Han Y. Combination of gut microbiota and plasma amyloid-β as a potential index for identifying preclinical Alzheimer’s disease: a cross-sectional analysis from the SILCODE study. Alzheimers Res Ther. 2022;14[1]:1-15.

161. Liu S, Li E, Sun Z, Fu D, Duan G, Jiang M, et al. Altered gut microbiota and short chain fatty acids in Chinese children with autism spectrum disorder. Sci Rep. 2019;9[1]:287.

162. Murros KE. Hydrogen sulfide produced by gut bacteria may induce Parkinson’s disease. Cells. 2022;11[6]:978.

163. Rhee SJ, Kim H, Lee Y, Lee HJ, Park C, Yang J, et al. The association between serum microbial DNA composition and symptoms of depression and anxiety in mood disorders. Sci Rep. 2021;11[1]:1-9.

164. Szabo H, Piroska M, Hernyes A, Zoldi L, Juhasz J, Ligeti B, et al. The Relationship between Atherosclerosis and Gut Microbiome in Patients with Obstructive Sleep Apnoea. Applied Sciences. 2022;12[22]:11484.

165. Baldelli V, Scaldaferri F, Putignani L, Del Chierico F. The role of Enterobacteriaceae in gut microbiota dysbiosis in inflammatory bowel diseases. Microorganisms. 2021;9[4]:697.

166. Carroll IM, Ringel-Kulka T, Siddle JP, Ringel Y. Alterations in composition and diversity of the intestinal microbiota in patients with diarrhea-predominant irritable bowel syndrome. Neurogastroenterol Motil. 2012;24[6]:521-e248.

167. Million M, Diallo A, Raoult D. Gut microbiota and malnutrition. Microb Pathog. 2017;106:127-38.

168. Sánchez-Alcoholado L, Ordóñez R, Otero A, Plaza-Andrade I, Laborda-Illanes A, Medina JA, et al. Gut microbiota-mediated inflammation and gut permeability in patients with obesity and colorectal cancer. Int J Mol Sci. 2020;21[18]:6782.

169. Zhuang Z, Yang R, Wang W, Qi L, Huang T. Associations between gut microbiota and Alzheimer’s disease, major depressive disorder, and schizophrenia. J Neuroinflammation. 2020;17[1]:1-9.

170. Lou M, Cao A, Jin C, Mi K, Xiong X, Zeng Z, et al. Deviated and early unsustainable stunted development of gut microbiota in children with autism spectrum disorder. Gut. 2022;71[8]:1588-99.

171. Pietrucci D, Cerroni R, Unida V, Farcomeni A, Pierantozzi M, Mercuri NB, et al. Dysbiosis of gut microbiota in a selected population of Parkinson's patients. Parkinsonism Relat Disord. 2019;65:124-30.

172. Jiang H, Ling Z, Zhang Y, Mao H, Ma Z, Yin Y, et al. Altered fecal microbiota composition in patients with major depressive disorder. Brain Behav Immun. 2015;48:186-94.

173. Maki KA, Burke LA, Calik MW, Watanabe-Chailland M, Sweeney D, Romick-Rosendale LE, et al. Sleep fragmentation increases blood pressure and is associated with alterations in the gut microbiome and fecal metabolome in rats. Physiol Genomics. 2020.

174. Li P, Lu B, Gong J, Li L, Chen G, Zhang J, et al. Chickpea extract ameliorates metabolic syndrome symptoms via restoring intestinal ecology and metabolic profile in type 2 diabetic rats. Mol Nutr Food Res. 2021;65[13]:2100007.

175. Chen L, Wang W, Zhou R, Ng SC, Li J, Huang M, et al. Characteristics of fecal and mucosa-associated microbiota in Chinese patients with inflammatory bowel disease. Medicine. 2014;93[8].

176. Foley KP, Zlitni S, Duggan BM, Barra NG, Anhê FF, Cavallari JF, et al. Gut microbiota impairs insulin clearance in obese mice. Molecular Metabolism. 2020;42:101067.

177. Wu N, Yang X, Zhang R, Li J, Xiao X, Hu Y, et al. Dysbiosis signature of fecal microbiota in colorectal cancer patients. Microb Ecol. 2013;66:462-70.

178. Hou M, Xu G, Ran M, Luo W, Wang H. APOE-ε4 carrier status and gut microbiota dysbiosis in patients with Alzheimer disease. Front Neurosci. 2021;15:619051.

179. Liu RT, Rowan-Nash AD, Sheehan AE, Walsh RF, Sanzari CM, Korry BJ, et al. Reductions in anti-inflammatory gut bacteria are associated with depression in a sample of young adults. Brain Behav Immun. 2020;88:308-24.

180. Mancini A, Campagna F, Amodio P, Tuohy KM. Gut: liver: brain axis: the microbial challenge in the hepatic encephalopathy. Food Funct. 2018;9[3]:1373-88.

181. Moreno-Indias I, Torres M, Montserrat JM, Sanchez-Alcoholado L, Cardona F, Tinahones FJ, et al. Intermittent hypoxia alters gut microbiota diversity in a mouse model of sleep apnoea. Eur Respir J. 2015;45[4]:1055-65.

182. Vals-Delgado C, Alcala-Diaz JF, Molina-Abril H, Roncero-Ramos I, Caspers MP, Schuren FH, et al. An altered microbiota pattern precedes Type 2 diabetes mellitus development: From the CORDIOPREV study. Journal of Advanced Research. 2022;35:99-108.

183. Pozuelo M, Panda S, Santiago A, Mendez S, Accarino A, Santos J, et al. Reduction of butyrate-and methane-producing microorganisms in patients with Irritable Bowel Syndrome. Sci Rep. 2015;5[1]:12693.

184. Nakayama J, Yamamoto A, Palermo-Conde LA, Higashi K, Sonomoto K, Tan J, et al. Impact of westernized diet on gut microbiota in children on Leyte Island. Front Microbiol. 2017;8:197.

185. Zhang H, DiBaise JK, Zuccolo A, Kudrna D, Braidotti M, Yu Y, et al. Human gut microbiota in obesity and after gastric bypass. Proceedings of the National Academy of Sciences. 2009;106[7]:2365-70.

186. Zackular JP, Baxter NT, Iverson KD, Sadler WD, Petrosino JF, Chen GY, et al. The gut microbiome modulates colon tumorigenesis. MBio. 2013;4[6]:e00692-13.

187. Jung JH, Kim G, Byun MS, Lee JH, Yi D, Park H, et al. Gut microbiome alterations in preclinical Alzheimer’s disease. PLoS One. 2022;17[11]:e0278276.

188. Bedarf JR, Hildebrand F, Coelho LP, Sunagawa S, Bahram M, Goeser F, et al. Functional implications of microbial and viral gut metagenome changes in early stage L-DOPA-naïve Parkinson’s disease patients. Genome Med. 2017;9:1-13.

189. Tan J, Li X, Zhu Y, Sullivan MA, Deng B, Zhai X, et al. Antidepressant shugan jieyu capsule alters gut microbiota and intestinal microbiome function in rats with chronic unpredictable mild stress-induced depression. Front Pharmacol. 2022;13.

190. Yuan J. Fecal microbiota and metabolome characteristics in cirrhotic patients accompanied with hepatic encephalopathy. 2019.

191. Davies N, O’Sullivan JM, Plank LD, Murphy R. Gut microbial predictors of type 2 diabetes remission following bariatric surgery. Obes Surg. 2020;30:3536-48.

192. Willing BP, Dicksved J, Halfvarson J, Andersson AF, Lucio M, Zheng Z, et al. A pyrosequencing study in twins shows that gastrointestinal microbial profiles vary with inflammatory bowel disease phenotypes. Gastroenterology. 2010;139[6]:1844-54. e1.

193. Hollister EB, Cain KC, Shulman RJ, Jarrett ME, Burr RL, Ko C, et al. Relationships of microbiome markers with extra-intestinal, psychological distress and gastrointestinal symptoms, and quality of life in women with irritable bowel syndrome. J Clin Gastroenterol. 2020;54[2]:175.

194. Romo-Vaquero M, Cortés-Martín A, Loria-Kohen V, Ramírez-de-Molina A, García-Mantrana I, Collado MC, et al. Deciphering the human gut microbiome of urolithin metabotypes: association with enterotypes and potential cardiometabolic health implications. Mol Nutr Food Res. 2019;63[4]:1800958.

195. Wang Y, Wan X, Wu X, Zhang C, Liu J, Hou S. Eubacterium rectale contributes to colorectal cancer initiation via promoting colitis. Gut Pathog. 2021;13[1]:1-11.

196. Zhang L, Wang Y, Xiayu X, Shi C, Chen W, Song N, et al. Altered gut microbiota in a mouse model of Alzheimer’s disease. J Alzheimers Dis. 2017;60[4]:1241-57.

197. Vascellari S, Melis M, Palmas V, Pisanu S, Serra A, Perra D, et al. Clinical phenotypes of Parkinson’s disease associate with distinct gut microbiota and metabolome enterotypes. Biomolecules. 2021;11[2]:144.

198. Pourdeh EF, Ulker I. Do all Bariatric Surgery Methods Have the Same Effects on the Gut Microbiota? Bariatric Surgery-Past and Present: IntechOpen; 2022.

199. Villanueva-Millan MJ, Leite G, Wang J, Morales W, Parodi G, Pimentel ML, et al. Methanogens and hydrogen sulfide producing bacteria guide distinct gut microbe profiles and irritable bowel syndrome subtypes. The American Journal of Gastroenterology. 2022;117[12]:2055-66.

200. Moreno-Indias I, Sánchez-Alcoholado L, García-Fuentes E, Cardona F, Queipo-Ortuño MI, Tinahones FJ. Insulin resistance is associated with specific gut microbiota in appendix samples from morbidly obese patients. American journal of translational research. 2016;8[12]:5672.

201. Taddese R, Garza DR, Ruiter LN, de Jonge MI, Belzer C, Aalvink S, et al. Growth rate alterations of human colorectal cancer cells by 157 gut bacteria. Gut Microbes. 2020;12[1]:1799733.

202. Mancuso C, Santangelo R. Alzheimer’s disease and gut microbiota modifications: the long way between preclinical studies and clinical evidence. Pharmacol Res. 2018;129:329-36.

203. Abrishamkar P, Rodriguez CVB, Choudry B, Or S. Antidepressant usage is associated with alterations in gut microbiota diversity and abundance in Parkinson’s Disease patients. Undergraduate Journal of Experimental Microbiology and Immunology. 2022;8.

204. Luo F, Fang C. Association between gut microbiota and post-stroke depression in Chinese population: A meta-analysis. Heliyon. 2022:e12605.

205. Shin NR, Gu N, Choi HS, Kim H. Combined effects of Scutellaria baicalensis with metformin on glucose tolerance of patients with type 2 diabetes via gut microbiota modulation. American Journal of Physiology-Endocrinology and Metabolism. 2020;318[1]:E52-E61.

206. Liu B, Zhang Y, Wang R, An Y, Gao W, Bai L, et al. Western diet feeding influences gut microbiota profiles in apoE knockout mice. Lipids Health Dis. 2018;17[1]:1-8.

207. Coker OO, Nakatsu G, Dai RZ, Wu WKK, Wong SH, Ng SC, et al. Enteric fungal microbiota dysbiosis and ecological alterations in colorectal cancer. Gut. 2019;68[4]:654-62.

208. Moreno-Indias I, Torres M, Sanchez-Alcoholado L, Cardona F, Almendros I, Gozal D, et al. Normoxic recovery mimicking treatment of sleep apnea does not reverse intermittent hypoxia-induced bacterial dysbiosis and low-grade endotoxemia in mice. Sleep. 2016;39[10]:1891-7.

209. Han L, Li T, Du M, Chang R, Zhan B, Mao X. Beneficial effects of potentilla discolor bunge water extract on inflammatory cytokines release and gut microbiota in high-fat diet and streptozotocin-induced type 2 diabetic mice. Nutrients. 2019;11[3]:670.

210. Soltys K, Stuchlikova M, Hlavaty T, Gaalova B, Budis J, Gazdarica J, et al. Seasonal changes of circulating 25-hydroxyvitamin D correlate with the lower gut microbiome composition in inflammatory bowel disease patients. Sci Rep. 2020;10[1]:6024.

211. Wang X, Qi Q, Wang Y, Wu H, Jin X, Yao H, et al. Gut microbiota was modulated by moxibustion stimulation in rats with irritable bowel syndrome. Chin Med. 2018;13[1]:1-14.

212. Monira S, Nakamura S, Gotoh K, Izutsu K, Watanabe H, Alam NH, et al. Gut microbiota of healthy and malnourished children in Bangladesh. Front Microbiol. 2011;2:228.

213. Li L, Li R, Zhu R, Chen B, Tian Y, Zhang H, et al. Salvianolic acid B prevents body weight gain and regulates gut microbiota and LPS/TLR4 signaling pathway in high-fat diet-induced obese mice. Food Funct. 2020;11[10]:8743-56.

214. Wu M, Wu Y, Deng B, Li J, Cao H, Qu Y, et al. Isoliquiritigenin decreases the incidence of colitis-associated colorectal cancer by modulating the intestinal microbiota. Oncotarget. 2016;7[51]:85318.

215. Shen L, Liu L, Ji H-F. Alzheimer’s disease histological and behavioral manifestations in transgenic mice correlate with specific gut microbiome state. J Alzheimers Dis. 2017;56[1]:385-90.

216. Coretti L, Cristiano C, Florio E, Scala G, Lama A, Keller S, et al. Sex-related alterations of gut microbiota composition in the BTBR mouse model of autism spectrum disorder. Sci Rep. 2017;7[1]:1-10.

217. Sun J, Li H, Jin Y, Yu J, Mao S, Su K-P, et al. Probiotic Clostridium butyricum ameliorated motor deficits in a mouse model of Parkinson’s disease via gut microbiota-GLP-1 pathway. Brain Behav Immun. 2021;91:703-15.

218. Duan J, Huang Y, Tan X, Chai T, Wu J, Zhang H, et al. Characterization of gut microbiome in mice model of depression with divergent response to escitalopram treatment. Translational Psychiatry. 2021;11[1]:303.

219. Zhang C, Chen F, Shen Y, Chen Y, Ma J. Sleep apnea is associated with the increase of certain genera of Ruminococcaceae and Lachnospiraceae in the gut microbiome of hypertensive patients. Expert Rev Respir Med. 2022:1-10.

220. Zhao L, Lou H, Peng Y, Chen S, Zhang Y, Li X. Comprehensive relationships between gut microbiome and faecal metabolome in individuals with type 2 diabetes and its complications. Endocrine. 2019;66:526-37.

221. Maukonen J, Kolho K-L, Paasela M, Honkanen J, Klemetti P, Vaarala O, et al. Altered fecal microbiota in paediatric inflammatory bowel disease. Journal of Crohn's and Colitis. 2015;9[12]:1088-95.

222. Schulz N, Belheouane M, Dahmen B, Ruan VA, Specht HE, Dempfle A, et al. Gut microbiota alteration in adolescent anorexia nervosa does not normalize with short-term weight restoration. Int J Eat Disord. 2021;54[6]:969-80.

223. Ma L, Ni Y, Wang Z, Tu W, Ni L, Zhuge F, et al. Spermidine improves gut barrier integrity and gut microbiota function in diet-induced obese mice. Gut Microbes. 2020;12[1]:1832857.

224. Hexun Z, Miyake T, Maekawa T, Mori H, Yasukawa D, Ohno M, et al. High abundance of lachnospiraceae in the human gut microbiome is related to high immunoscores in advanced colorectal cancer. Cancer Immunol Immunother. 2022:1-12.

225. Ding X, Xu Y, Zhang X, Zhang L, Duan G, Song C, et al. Gut microbiota changes in patients with autism spectrum disorders. J Psychiatr Res. 2020;129:149-59.

226. Hill-Burns EM, Debelius JW, Morton JT, Wissemann WT, Lewis MR, Wallen ZD, et al. Parkinson's disease and Parkinson's disease medications have distinct signatures of the gut microbiome. Mov Disord. 2017;32[5]:739-49.

227. Zhang H-h, Liu J, Lv Y-j, Jiang Y-l, Pan J-x, Zhu Y-j, et al. Changes in intestinal microbiota of type 2 diabetes in mice in response to dietary supplementation with instant tea or matcha. Canadian journal of diabetes. 2020;44[1]:44-52.

228. Guo H, Yu L, Tian F, Chen W, Zhai Q. The Potential Therapeutic Role of Lactobacillaceae rhamnosus for Treatment of Inflammatory Bowel Disease. Foods. 2023;12[4]:692.

229. Taverniti V, Guglielmetti S. Methodological issues in the study of intestinal microbiota in irritable bowel syndrome. World Journal of Gastroenterology: WJG. 2014;20[27]:8821.

230. Ye J, Zhao Y, Chen X, Zhou H, Yang Y, Zhang X, et al. Pu-erh tea ameliorates obesity and modulates gut microbiota in high fat diet fed mice. Food Research International. 2021;144:110360.

231. Zhao L, Zhang X, Zuo T, Yu J. The composition of colonic commensal bacteria according to anatomical localization in colorectal cancer. Engineering. 2017;3[1]:90-7.

232. Fu S-C, Lee C-H, Wang H. Exploring the association of autism spectrum disorders and constipation through analysis of the gut microbiome. Int J Environ Res Public Health. 2021;18[2]:667.

233. Hopfner F, Künstner A, Müller SH, Künzel S, Zeuner KE, Margraf NG, et al. Gut microbiota in Parkinson disease in a northern German cohort. Brain Res. 2017;1667:41-5.

234. Chevalier G, Siopi E, Guenin-Macé L, Pascal M, Laval T, Rifflet A, et al. Effect of gut microbiota on depressive-like behaviors in mice is mediated by the endocannabinoid system. Nature Communications. 2020;11[1]:6363.

235. Zhang C, Zhang T, Lu W, Duan X, Luo X, Liu S, et al. Altered airway microbiota composition in patients with pulmonary hypertension. Hypertension. 2020;76[5]:1589-99.

236. Balmasova IP, Olekhnovich EI, Klimina KM, Korenkova AA, Vakhitova MT, Babaev EA, et al. Drift of the subgingival periodontal microbiome during chronic periodontitis in Type 2 diabetes mellitus patients. Pathogens. 2021;10[5]:504.

237. Hourigan S, Chen L, Grigoryan Z, Laroche G, Weidner M, Sears C, et al. Microbiome changes associated with sustained eradication of Clostridium difficile after single faecal microbiota transplantation in children with and without inflammatory bowel disease. Aliment Pharmacol Ther. 2015;42[6]:741-52.

238. Shin S, Cho KY. Altered gut microbiota and shift in Bacteroidetes between young obese and normal-weight Korean children: a cross-sectional observational study. BioMed Research International. 2020;2020.

239. Wang P, Ding S, Sun L, Feng Y, Guo K, Zhu Y, et al. Characteristics and differences of gut microbiota in patients with different traditional Chinese medicine syndromes of colorectal cancer and normal population. J Cancer. 2020;11[24]:7357.

240. Chen L, Xu X, Wu X, Cao H, Li X, Hou Z, et al. A comparison of the composition and functions of the oral and gut microbiotas in Alzheimer's patients. Frontiers in Cellular and Infection Microbiology. 2022:1241.

241. Shen L. Gut, oral and nasal microbiota and Parkinson's disease. Microbial Cell Factories. 2020;19[1]:50.

242. Tien NTT. Analysis of the gut microbiota of Japanese Alzheimer's disease patients and characterization of their butyrate-producing bacteria. 2018.

243. Chen Z, Xie Y, Zhou F, Zhang B, Wu J, Yang L, et al. Featured gut microbiomes associated with the progression of chronic hepatitis B disease. Front Microbiol. 2020;11:383.

244. Gonzalez-Mercado VJ, Lim J, Saligan LN, Perez N, Rodriguez C, Bernabe R, et al. Gut microbiota and depressive symptoms at the end of CRT for rectal cancer: A cross-sectional pilot study. Depression Research and Treatment. 2021;2021.

245. Zhang Y, Lu S, Yang Y, Wang Z, Wang B, Zhang B, et al. The diversity of gut microbiota in type 2 diabetes with or without cognitive impairment. Aging Clin Exp Res. 2021;33:589-601.

246. Knights D, Lassen KG, Xavier RJ. Advances in inflammatory bowel disease pathogenesis: linking host genetics and the microbiome. Gut. 2013;62[10]:1505-10.

247. Yun Y, Kim H-N, Lee E-j, Ryu S, Chang Y, Shin H, et al. Fecal and blood microbiota profiles and presence of nonalcoholic fatty liver disease in obese versus lean subjects. PLoS One. 2019;14[3]:e0213692.

248. Kim M-H, Yun KE, Kim J, Park E, Chang Y, Ryu S, et al. Gut microbiota and metabolic health among overweight and obese individuals. Sci Rep. 2020;10[1]:1-11.

249. Park J-Y, Choi J, Lee Y, Lee J-E, Lee E-H, Kwon H-J, et al. Metagenome analysis of bodily microbiota in a mouse model of Alzheimer disease using bacteria-derived membrane vesicles in blood. Exp Neurobiol. 2017;26[6]:369.

250. Li F, Wang P, Chen Z, Sui X, Xie X, Zhang J. Alteration of the fecal microbiota in North-Eastern Han Chinese population with sporadic Parkinson's disease. Neurosci Lett. 2019;707:134297.

251. Tsai C-F, Chuang C-H, Wang Y-P, Lin Y-B, Tu P-C, Liu P-Y, et al. Differences in gut microbiota correlate with symptoms and regional brain volumes in patients with late-life depression. Front Aging Neurosci. 2022;14.

252. Xiang K, Zhang J-J, Xu Y-Y, Zhong X, Ni J, Pan H-F. Genetically predicted causality of 28 gut microbiome families and type 2 diabetes mellitus risk. Front Endocrinol [Lausanne]. 2022;13:9.

253. Danilova N, Abdulkhakov S, Grigoryeva T, Dubinkina V, Odintsova V, Tyakht A, et al. P794 Alterations of intestinal microbiota in ulcerative colitis. Journal of Crohn's and Colitis. 2017;11[suppl_1]:S486-S.

254. Chen H, Ou R, Tang N, Su W, Yang R, Yu X, et al. Alternation of the gut microbiota in irritable bowel syndrome: an integrated analysis based on multicenter amplicon sequencing data. J Transl Med. 2023;21[1]:1-16.

255. Smitka K, Prochazkova P, Roubalova R, Dvorak J, Papezova H, Hill M, et al. Current aspects of the role of autoantibodies directed against appetite-regulating hormones and the gut microbiome in eating disorders. Front Endocrinol [Lausanne]. 2021;12:613983.

256. Aranaz P, Ramos-Lopez O, Cuevas-Sierra A, Martinez JA, Milagro FI, Riezu-Boj JI. A predictive regression model of the obesity-related inflammatory status based on gut microbiota composition. Int J Obes. 2021;45[10]:2261-8.

257. Long S, Yang Y, Shen C, Wang Y, Deng A, Qin Q, et al. Metaproteomics characterizes human gut microbiome function in colorectal cancer. NPJ biofilms and microbiomes. 2020;6[1]:14.

258. Petrov V, Saltykova I, Zhukova I, Alifirova V, Zhukova N, Dorofeeva YB, et al. Analysis of gut microbiota in patients with Parkinson's disease. Bull Exp Biol Med. 2017;162:734-7.

259. Fontana A, Manchia M, Panebianco C, Paribello P, Arzedi C, Cossu E, et al. Exploring the role of gut microbiota in major depressive disorder and in treatment resistance to antidepressants. Biomedicines. 2020;8[9]:311.

260. Ponziani FR, Bhoori S, Castelli C, Putignani L, Rivoltini L, Del Chierico F, et al. Hepatocellular carcinoma is associated with gut microbiota profile and inflammation in nonalcoholic fatty liver disease. Hepatology. 2019;69[1]:107-20.

261. Zhang F, Chen D, Zhang L, Zhao Q, Ma Y, Zhang X, et al. Diaphragma juglandis extracts modifies the gut microbiota during prevention of type 2 diabetes in rats. J Ethnopharmacol. 2022;283:114484.

262. Wang K, Guo J, Chang X, Gui S. Painong-San extract alleviates dextran sulfate sodium-induced colitis in mice by modulating gut microbiota, restoring intestinal barrier function and attenuating TLR4/NF-κB signaling cascades. J Pharm Biomed Anal. 2022;209:114529.

263. Sampsell K, Wang W, Ohland C, Mager LF, Pett N, Lowry DE, et al. Exercise and Prebiotic Fiber Provide Gut Microbiota-Driven Benefit in a Survivor to Germ-Free Mouse Translational Model of Breast Cancer. Cancers [Basel]. 2022;14[11]:2722.

264. Maya-Lucas O, Murugesan S, Nirmalkar K, Alcaraz LD, Hoyo-Vadillo C, Pizano-Zárate ML, et al. The gut microbiome of Mexican children affected by obesity. Anaerobe. 2019;55:11-23.

265. Qi Z, Zhibo Z, Jing Z, Zhanbo Q, Shugao H, Weili J, et al. Prediction model of poorly differentiated colorectal cancer [CRC] based on gut bacteria. BMC Microbiol. 2022;22[1]:312.

266. Jones J, Reinke SN, Mousavi-Derazmahalleh M, Palmer DJ, Christophersen CT. Changes to the gut microbiome in young children showing early behavioral signs of autism. Front Microbiol. 2022;13.

267. Romano S, Savva GM, Bedarf JR, Charles IG, Hildebrand F, Narbad A. Meta-analysis of the Parkinson's disease gut microbiome suggests alterations linked to intestinal inflammation. npj Parkinson's Disease. 2021;7[1]:27.

268. Liu X, Li X, Teng T, Jiang Y, Xiang Y, Fan L, et al. Comparative analysis of gut microbiota and fecal metabolome features among multiple depressive animal models. J Affect Disord. 2022;314:103-11.

269. Jian J, Nie M, Xiang B, Qian H, Yin C, Zhang X, et al. Rifaximin ameliorates non-alcoholic steatohepatitis in mice through regulating gut microbiome-related bile acids. Front Pharmacol. 2022:1099.

270. Maskarinec G, Raquinio P, Kristal BS, Setiawan VW, Wilkens LR, Franke AA, et al. The gut microbiome and type 2 diabetes status in the Multiethnic Cohort. PLoS One. 2021;16[6]:e0250855.

271. Rengarajan S, Vivio EE, Parkes M, Peterson DA, Roberson ED, Newberry RD, et al. Dynamic immunoglobulin responses to gut bacteria during inflammatory bowel disease. Gut Microbes. 2020;11[3]:405-20.

272. Shivakumar N, Sivadas A, Devi S, Jahoor F, McLaughlin J, Smith CP, et al. Gut microbiota profiles of young South Indian children: Child sex-specific relations with growth. PLoS One. 2021;16[5]:e0251803.

273. Schott EM, Farnsworth CW, Grier A, Lillis JA, Soniwala S, Dadourian GH, et al. Targeting the gut microbiome to treat the osteoarthritis of obesity. JCI insight. 2018;3[8].

274. Cheng Y, Ling Z, Li L. The intestinal microbiota and colorectal cancer. Front Immunol. 2020;11:615056.

275. Li P, Killinger BA, Ensink E, Beddows I, Yilmaz A, Lubben N, et al. Gut microbiota dysbiosis is associated with elevated bile acids in Parkinson's disease. Metabolites. 2021;11[1]:29.

276. Gu Y, Chen H, Li X, Li D, Sun Y, Yang L, et al. Lactobacillus paracasei IMC 502 ameliorates type 2 diabetes by mediating gut microbiota–SCFA–hormone/inflammation pathway in mice. J Sci Food Agric. 2022.

277. Morgan XC, Tickle TL, Sokol H, Gevers D, Devaney KL, Ward DV, et al. Dysfunction of the intestinal microbiome in inflammatory bowel disease and treatment. Genome Biol. 2012;13[9]:1-18.

278. Clarke SF, Murphy EF, O'Sullivan O, Ross RP, O'Toole PW, Shanahan F, et al. Targeting the microbiota to address diet-induced obesity: a time dependent challenge. PLoS One. 2013;8[6]:e65790.

279. Zhang M, Zhao D, Zhou G, Li C. Dietary pattern, gut microbiota, and Alzheimer's disease. J Agric Food Chem. 2020;68[46]:12800-9.

280. Inoue R, Sakaue Y, Sawai C, Sawai T, Ozeki M, Romero-Pérez GA, et al. A preliminary investigation on the relationship between gut microbiota and gene expressions in peripheral mononuclear cells of infants with autism spectrum disorders. Biosci Biotechnol Biochem. 2016;80[12]:2450-8.

281. Ren T, Gao Y, Qiu Y, Jiang S, Zhang Q, Zhang J, et al. Gut microbiota altered in mild cognitive impairment compared with normal cognition in sporadic Parkinson's disease. Front Neurol. 2020;11:137.

282. Sun L, Ma L, Zhang H, Cao Y, Wang C, Hou N, et al. Fto deficiency reduces anxiety-and depression-like behaviors in mice via alterations in gut microbiota. Theranostics. 2019;9[3]:721.

283. Bajaj JS. The role of microbiota in hepatic encephalopathy. Gut microbes. 2014;5[3]:397-403.

284. Palm NW, De Zoete MR, Cullen TW, Barry NA, Stefanowski J, Hao L, et al. Immunoglobulin A coating identifies colitogenic bacteria in inflammatory bowel disease. Cell. 2014;158[5]:1000-10.

285. Cuevas-Sierra A, Riezu-Boj JI, Guruceaga E, Milagro FI, Martínez JA. Sex-specific associations between gut Prevotellaceae and host genetics on adiposity. Microorganisms. 2020;8[6]:938.

286. Hang Z, Lei T, Zeng Z, Cai S, Bi W, Du H. Composition of intestinal flora affects the risk relationship between Alzheimer's disease/Parkinson's disease and cancer. Biomed Pharmacother. 2022;145:112343.

287. Sun H, You Z, Jia L, Wang F. Autism spectrum disorder is associated with gut microbiota disorder in children. BMC Pediatr. 2019;19:1-7.

288. Scheperjans F, Aho V, Pereira PA, Koskinen K, Paulin L, Pekkonen E, et al. Gut microbiota are related to Parkinson's disease and clinical phenotype. Mov Disord. 2015;30[3]:350-8.

289. Wu J, Li J, Gaurav C, Muhammad U, Chen Y, Li X, et al. CUMS and dexamethasone induce depression-like phenotypes in mice by differentially altering gut microbiota and triggering macroglia activation. General psychiatry. 2021;34[6].

290. Bajaj JS, Betrapally NS, Hylemon PB, Heuman DM, Daita K, White MB, et al. Salivary microbiota reflects changes in gut microbiota in cirrhosis with hepatic encephalopathy. Hepatology. 2015;62[4]:1260-71.

291. Čipčić Paljetak H, Barešić A, Panek M, Perić M, Matijašić M, Lojkić I, et al. Gut microbiota in mucosa and feces of newly diagnosed, treatment-naïve adult inflammatory bowel disease and irritable bowel syndrome patients. Gut Microbes. 2022;14[1]:2083419.

292. Ahasan MS, Waltzek TB, Owens L, Ariel E. Characterisation and comparison of the mucosa-associated bacterial communities across the gastrointestinal tract of stranded green turtles, Chelonia mydas. AIMS microbiology. 2020;6[4]:361.

293. Orbe-Orihuela YC, Godoy-Lozano EE, Lagunas-Martínez A, Castañeda-Márquez AC, Murga-Garrido S, Díaz-Benítez CE, et al. Association of Gut Microbiota with Dietary-dependent Childhood Obesity. Arch Med Res. 2022;53[4]:407-15.

294. Phipps O, Al-Hassi HO, Quraishi MN, Dickson EA, Segal J, Steed H, et al. Oral and intravenous iron therapy differentially alter the on-and off-tumor microbiota in anemic colorectal cancer patients. Cancers [Basel]. 2021;13[6]:1341.

295. Ghyselinck J, Verstrepen L, Moens F, Van Den Abbeele P, Bruggeman A, Said J, et al. Influence of probiotic bacteria on gut microbiota composition and gut wall function in an in-vitro model in patients with Parkinson's disease. International Journal of Pharmaceutics: X. 2021;3:100087.

296. Kelly JR, Borre Y, O'Brien C, Patterson E, El Aidy S, Deane J, et al. Transferring the blues: depression-associated gut microbiota induces neurobehavioural changes in the rat. J Psychiatr Res. 2016;82:109-18.

297. Bajaj J. Potential mechanisms of action of rifaximin in the management of hepatic encephalopathy and other complications of cirrhosis. Aliment Pharmacol Ther. 2016;43:11-26.

298. Eun CS, Kwak M-J, Han DS, Lee AR, Park DI, Yang S-K, et al. Does the intestinal microbial community of Korean Crohn’s disease patients differ from that of western patients? BMC Gastroenterol. 2016;16:1-11.

299. Tang B, Hu Y, Chen J, Su C, Zhang Q, Huang C. Oral and fecal microbiota in patients with diarrheal irritable bowel syndrome. Heliyon. 2023:e13114.

300. Wang S-T, Meng X-Z, Zhang J-H, Dai Y-F, Shen Y, Xu X-Y, et al. 16S rRNA sequencing analysis of the correlation between the intestinal microbiota and body-mass of grass carp [Ctenopharyngodon idella]. Comparative Biochemistry and Physiology Part D: Genomics and Proteomics. 2020;35:100699.

301. Yuan W, Lu W, Wang H, Wu W, Zhou Q, Chen Y, et al. A multiphase dietetic protocol incorporating an improved ketogenic diet enhances weight loss and alters the gut microbiome of obese people. Int J Food Sci Nutr. 2022;73[2]:238-50.

302. Yang J, McDowell A, Kim EK, Seo H, Lee WH, Moon C-M, et al. Development of a colorectal cancer diagnostic model and dietary risk assessment through gut microbiome analysis. Exp Mol Med. 2019;51[10]:1-15.

303. Park S, Wu X. Modulation of the Gut Microbiota in Memory Impairment and Alzheimer’s Disease via the Inhibition of the Parasympathetic Nervous System. Int J Mol Sci. 2022;23[21]:13574.

304. Zhu H, Zhang H, Hou B, Xu B, Ji L, Wu Y. Curcumin Regulates Gut Microbiota and Exerts a Neuroprotective Effect in the MPTP Model of Parkinson’s Disease. Evid Based Complement Alternat Med. 2022;2022.

305. Yu M, Jia H-M, Qin L-L, Zou Z-M. Gut microbiota and gut tissue metabolites involved in development and prevention of depression. J Affect Disord. 2022;297:8-17.

306. Wang JY, Bajaj JS, Wang JB, Shang J, Zhou XM, Guo XL, et al. Lactulose improves cognition, quality of life, and gut microbiota in minimal hepatic encephalopathy: A multicenter, randomized controlled trial. J Dig Dis. 2019;20[10]:547-56.

307. Lin R, He X, Chen H, He Q, Yao Z, Li Y, et al. Oil tea improves glucose and lipid levels and alters gut microbiota in type 2 diabetic mice. Nutr Res. 2018;57:67-77.

308. Dou X, Gao N, Yan D, Shan A. Sodium butyrate alleviates mouse colitis by regulating gut microbiota dysbiosis. Animals. 2020;10[7]:1154.

309. Lian Q, Ding H, Zhu H, Zhang C, Yu S, Jie H, et al. Study of Jianpi mixture on intestinal microbiota of diarrhea irritable bowel syndrome mice. Evid Based Complement Alternat Med. 2020;2020.

310. Du R, Jiao S, Dai Y, An J, Lv J, Yan X, et al. Probiotic Bacillus amyloliquefaciens C-1 improves growth performance, stimulates GH/IGF-1, and regulates the gut microbiota of growth-retarded beef calves. Front Microbiol. 2018;9:2006.

311. Koliarakis I, Athanasakis E, Sgantzos M, Mariolis-Sapsakos T, Xynos E, Chrysos E, et al. Intestinal microbiota in colorectal cancer surgery. Cancers [Basel]. 2020;12[10]:3011.

312. Zhou Y, Wang Y, Quan M, Zhao H, Jia J. Gut microbiota changes and their correlation with cognitive and neuropsychiatric symptoms in Alzheimer's disease. J Alzheimers Dis. 2021;81[2]:583-95.

313. Petrak F, Herpertz S, Hirsch J, Röhrig B, Donati-Hirsch I, Juckel G, et al. Gut microbiota differs in composition between adults with type 1 diabetes with or without depression and healthy control participants: a case-control study. BMC Microbiol. 2022;22[1]:169.

314. Badran M, Khalyfa A, Ericsson A, Gozal D. Fecal microbiota transplantation from mice exposed to chronic intermittent hypoxia elicits sleep disturbances in naïve mice. Exp Neurol. 2020;334:113439.

315. De Palma G, Lynch MD, Lu J, Dang VT, Deng Y, Jury J, et al. Transplantation of fecal microbiota from patients with irritable bowel syndrome alters gut function and behavior in recipient mice. Sci Transl Med. 2017;9[379]:eaaf6397.

316. Bai J, Hu Y, Bruner D. Composition of gut microbiota and its association with body mass index and lifestyle factors in a cohort of 7–18 years old children from the American Gut Project. Pediatr Obes. 2019;14[4]:e12480.

317. Bäuerl C, Collado MC, Diaz Cuevas A, Viña J, Pérez Martínez G. Shifts in gut microbiota composition in an APP/PSS1 transgenic mouse model of Alzheimer's disease during lifespan. Blackwell Science Ltd Oxford, UK; 2018. p. 464-71.

318. Jin M, Li J, Liu F, Lyu N, Wang K, Wang L, et al. Analysis of the gut microflora in patients with Parkinson's disease. Front Neurosci. 2019;13:1184.

319. Yang J, Zhang Z, Xie Z, Bai L, Xiong P, Chen F, et al. Metformin modulates microbiota-derived inosine and ameliorates methamphetamine-induced anxiety and depression-like withdrawal symptoms in mice. Biomed Pharmacother. 2022;149:112837.

320. Chassard C, Dapoigny M, Scott KP, Crouzet L, Del'Homme C, Marquet P, et al. Functional dysbiosis within the gut microbiota of patients with constipated-irritable bowel syndrome. Aliment Pharmacol Ther. 2012;35[7]:828-38.

321. Morissette B, Talbot G, Beaulieu C, Lessard M. Growth performance of piglets during the first two weeks of lactation affects the development of the intestinal microbiota. J Anim Physiol Anim Nutr [Berl]. 2018;102[2]:525-32.

322. Liu B-N, Liu X-T, Liang Z-H, Wang J-H. Gut microbiota in obesity. World J Gastroenterol. 2021;27[25]:3837.

323. Tran TT, Corsini S, Kellingray L, Hegarty C, Le Gall G, Narbad A, et al. APOE genotype influences the gut microbiome structure and function in humans and mice: relevance for Alzheimer's disease pathophysiology. The FASEB Journal. 2019;33[7]:8221.

324. Onyema Oshim I, Regina Agbakoba N, Celestine Oguejiofor O, Anukam KC. Selective Microbial Biomarkers in Type-2 Diabetes with Principal Component Analysis and Receiver-operating Characteristic Curves. International Journal of Scientific Research in Dental and Medical Sciences. 2021;3[1]:23-34.

325. Chen H, Ou R, Tang N, Su W, Yang R, Yu X, et al. An Integrated Multicenter Amplicon Sequencing Data Reveals New Evidence of the Interaction Between the Gut Microbiota and Irritable Bowel Syndrome. 2022.

326. Lan D, Ji W, Lin B, Chen Y, Huang C, Xiong X, et al. Correlations between gut microbiota community structures of Tibetans and geography. Sci Rep. 2017;7[1]:16982.

327. Liu J, Dong W, Zhao J, Wu J, Xia J, Xie S, et al. Gut microbiota profiling variated during colorectal cancer development in mouse. BMC Genomics. 2022;23[4]:1-13.

328. Tartaglione AM, Villani A, Ajmone-Cat MA, Minghetti L, Ricceri L, Pazienza V, et al. Maternal immune activation induces autism-like changes in behavior, neuroinflammatory profile and gut microbiota in mouse offspring of both sexes. Translational Psychiatry. 2022;12[1]:384.

329. Vascellari S, Palmas V, Melis M, Pisanu S, Cusano R, Uva P, et al. Gut microbiota and metabolome alterations associated with Parkinson's disease. Msystems. 2020;5[5]:e00561-20.

330. Zhang P, Zhang D, Lai J, Fu Y, Wu L, Huang H, et al. Characteristics of the gut microbiota in bipolar depressive disorder patients with distinct weight. CNS Neurosci Ther. 2023.

331. Hyun C-K. Molecular and pathophysiological links between metabolic disorders and inflammatory bowel diseases. Int J Mol Sci. 2021;22[17]:9139.

332. Ponziani FR, Pompili M, Gasbarrini A. Rifaximin re-treatment in patients with irritable bowel syndrome: feels like the first time? : Springer; 2017. p. 2220-2.

333. Coley EJ, Hsiao EY. Malnutrition and the microbiome as modifiers of early neurodevelopment. Trends Neurosci. 2021;44[9]:753-64.

334. Ngowi EE, Wang YZ, Khattak S, Khan NH, Mahmoud SSM, Helmy YASH, et al. Impact of the factors shaping gut microbiota on obesity. J Appl Microbiol. 2021;131[5]:2131-47.

335. Raskov H, Burcharth J, Pommergaard H-C. Linking gut microbiota to colorectal cancer. J Cancer. 2017;8[17]:3378.

336. Liu J, Gao Z, Liu C, Liu T, Gao J, Cai Y, et al. Alteration of Gut Microbiota: New Strategy for Treating Autism Spectrum Disorder. Frontiers in Cell and Developmental Biology. 2022;10:192.

337. Yan Z, Li R, Shi W, Yao L. Role of the gut-microbiota-metabolite axis in the rotenone model of early-stage Parkinson's Disease. Metab Brain Dis. 2022;37[7]:2511-20.

338. Song X, Wang W, Ding S, Liu X, Wang Y, Ma H. Puerarin ameliorates depression-like behaviors of with chronic unpredictable mild stress mice by remodeling their gut microbiota. J Affect Disord. 2021;290:353-63.

339. Lachar J, Bajaj JS, editors. Changes in the microbiome in cirrhosis and relationship to complications: hepatic encephalopathy, spontaneous bacterial peritonitis, and sepsis. Semin Liver Dis; 2016: Thieme Medical Publishers.

340. Sanchez-Alcoholado L, Castellano-Castillo D, Jordán-Martínez L, Moreno-Indias I, Cardila-Cruz P, Elena D, et al. Role of gut microbiota on cardio-metabolic parameters and immunity in coronary artery disease patients with and without type-2 diabetes mellitus. Front Microbiol. 2017;8:1936.

341. Staudacher HM, Scholz M, Lomer MC, Ralph FS, Irving PM, Lindsay JO, et al. Gut microbiota associations with diet in irritable bowel syndrome and the effect of low FODMAP diet and probiotics. Clin Nutr. 2021;40[4]:1861-70.

342. Zeng H, Ishaq SL, Zhao F-Q, Wright A-DG. Colonic inflammation accompanies an increase of β-catenin signaling and Lachnospiraceae/Streptococcaceae bacteria in the hind gut of high-fat diet-fed mice. J Nutr Biochem. 2016;35:30-6.

343. Wieczorska K, Stolarek M, Stec R. The role of the gut microbiome in colorectal cancer: where are we? Where are we going? Clin Colorectal Cancer. 2020;19[1]:5-12.

344. Altaib H, Nakamura K, Abe M, Badr Y, Yanase E, Nomura I, et al. Differences in the concentration of the fecal neurotransmitters GABA and glutamate are associated with microbial composition among healthy human subjects. Microorganisms. 2021;9[2]:378.

345. Mohamadkhani A. Gut microbiota and fecal metabolome perturbation in children with autism spectrum disorder. Middle East journal of digestive diseases. 2018;10[4]:205.

346. Zhao H, Jin K, Jiang C, Pan F, Wu J, Luan H, et al. A pilot exploration of multi-omics research of gut microbiome in major depressive disorders. Translational Psychiatry. 2022;12[1]:8.

347. Rocco A, Sgamato C, Compare D, Coccoli P, Nardone OM, Nardone G. Gut microbes and hepatic encephalopathy: from the old concepts to new perspectives. Frontiers in Cell and Developmental Biology. 2021;9:748253.

348. Almugadam BS, Liu Y, Chen S-m, Wang C-h, Shao C-y, Ren B-w, et al. Alterations of gut microbiota in type 2 diabetes individuals and the confounding effect of antidiabetic agents. Journal of diabetes research. 2020;2020.

349. Zhou X-Y, Li M, Li X, Long X, Zuo X-L, Hou X-H, et al. Visceral hypersensitive rats share common dysbiosis features with irritable bowel syndrome patients. World J Gastroenterol. 2016;22[22]:5211.

350. Zhai S, Qin S, Li L, Zhu L, Zou Z, Wang L. Dietary butyrate suppresses inflammation through modulating gut microbiota in high-fat diet-fed mice. FEMS Microbiol Lett. 2019;366[13]:fnz153.

351. Carson TL, Byrd DA, Smith KS, Carter D, Abaskaron M, Little RB, et al. A case-control study of the association between the gut microbiota and colorectal cancer: exploring the roles of diet, stress, and race. 2023.

352. Zhou R, Fan X, Schnabl B. Role of the intestinal microbiome in liver fibrosis development and new treatment strategies. Translational Research. 2019;209:22-38.

353. Li J, Jia S, Yuan C, Yu B, Zhang Z, Zhao M, et al. Jerusalem artichoke inulin supplementation ameliorates hepatic lipid metabolism in type 2 diabetes mellitus mice by modulating the gut microbiota and fecal metabolome. Food Funct. 2022;13[22]:11503-17.

354. Zuo W, Wang B, Bai X, Luan Y, Fan Y, Michail S, et al. 16S rRNA and metagenomic shotgun sequencing data revealed consistent patterns of gut microbiome signature in pediatric ulcerative colitis. Sci Rep. 2022;12[1]:6421.

355. Setiawan J, Chan N, Echavez C, Mathews A. Increased body mass is associated with decreased gut microbiome diversity in Parkinson's Disease patients. Undergraduate Journal of Experimental Microbiology and Immunology. 2022;8.

356. Fernández J, Saettone P, Franchini MC, Villar CJ, Lombó F. Antitumor bioactivity and gut microbiota modulation of polyhydroxybutyrate [PHB] in a rat animal model for colorectal cancer. Int J Biol Macromol. 2022;203:638-49.

357. Nagarajan A. Characterization of the Metabolic and Cognitive Features of a Novel Alzheimer's Disease Rat Model Along with Investigation of Its Gut Microbiota Change over Time: The University of Alabama at Birmingham; 2021.

358. Fattorusso A, Di Genova L, Dell'Isola GB, Mencaroni E, Esposito S. Autism spectrum disorders and the gut microbiota. Nutrients. 2019;11[3]:521.

359. Liu M, Huang B, Wang L, Lu Q, Liu R. Peanut skin procyanidins ameliorate insulin resistance via modulation of gut microbiota and gut barrier in type 2 diabetic mice. J Sci Food Agric. 2022;102[13]:5935-47.

360. Cheng S, Hu J, Wu X, Pan J-A, Jiao N, Li Y, et al. Altered gut microbiome in FUT2 loss-of-function mutants in support of personalized medicine for inflammatory bowel diseases. Journal of Genetics and Genomics. 2021;48[9]:771-80.

361. Zhao Q, Fu Y, Zhang F, Wang C, Yang X, Bai S, et al. Heat-Treated Adzuki Bean Protein Hydrolysates Reduce Obesity in Mice Fed a High-Fat Diet via Remodeling Gut Microbiota and Improving Metabolic Function. Mol Nutr Food Res. 2022;66[8]:2100907.

362. Park S-H, Lee JH, Shin J, Kim J-S, Cha B, Lee S, et al. Cognitive function improvement after fecal microbiota transplantation in Alzheimer's dementia patient: A case report. Curr Med Res Opin. 2021;37[10]:1739-44.

363. Ma L, Zhang J, Fujita Y, Shinno-Hashimoto H, Shan J, Wan X, et al. Effects of spleen nerve denervation on depression–like phenotype, systemic inflammation, and abnormal composition of gut microbiota in mice after administration of lipopolysaccharide: A role of brain–spleen axis. J Affect Disord. 2022;317:156-65.

364. Burz SD, Monnoye M, Philippe C, Farin W, Ratziu V, Strozzi F, et al. Fecal microbiota transplant from human to mice gives insights into the role of the gut microbiota in non-alcoholic fatty liver disease [NAFLD]. Microorganisms. 2021;9[1]:199.

365. Liu G, Liang L, Yu G, Li Q. Pumpkin polysaccharide modifies the gut microbiota during alleviation of type 2 diabetes in rats. Int J Biol Macromol. 2018;115:711-7.

366. Yao C, Li Y, Luo L, Xie F, Xiong Q, Li T, et al. Significant Differences in Gut Microbiota Between Irritable Bowel Syndrome with Diarrhea and Healthy Controls in Southwest China. Dig Dis Sci. 2022:1-22.

367. Nirmalkar K, Murugesan S, Pizano-Zárate ML, Villalobos-Flores LE, García-González C, Morales-Hernández RM, et al. Gut microbiota and endothelial dysfunction markers in obese Mexican children and adolescents. Nutrients. 2018;10[12]:2009.

368. Ahn J, Sinha R, Pei Z, Dominianni C, Wu J, Shi J, et al. Human gut microbiome and risk for colorectal cancer. J Natl Cancer Inst. 2013;105[24]:1907-11.

369. Liu Q, Xi Y, Wang Q, Liu J, Li P, Meng X, et al. Mannan oligosaccharide attenuates cognitive and behavioral disorders in the 5xFAD Alzheimer's disease mouse model via regulating the gut microbiota-brain axis. Brain Behav Immun. 2021;95:330-43.

370. Krajmalnik-Brown R, Lozupone C, Kang D-W, Adams JB. Gut bacteria in children with autism spectrum disorders: challenges and promise of studying how a complex community influences a complex disease. Microb Ecol Health Dis. 2015;26[1]:26914.

371. Sanada K, Nakajima S, Kurokawa S, Barceló-Soler A, Ikuse D, Hirata A, et al. Gut microbiota and major depressive disorder: A systematic review and meta-analysis. J Affect Disord. 2020;266:1-13.

372. Gurung M, Li Z, You H, Rodrigues R, Jump DB, Morgun A, et al. Role of gut microbiota in type 2 diabetes pathophysiology. EBioMedicine. 2020;51:102590.

373. Pittayanon R, Lau JT, Leontiadis GI, Tse F, Yuan Y, Surette M, et al. Differences in Gut Microbiota in Patients With vs Without Inflammatory Bowel Diseases: A Systematic Review. Gastroenterology. 2020;158[4]:930-46 e1.

374. Bhattarai Y, Muniz Pedrogo DA, Kashyap PC. Irritable bowel syndrome: a gut microbiota-related disorder? American Journal of Physiology-Gastrointestinal and Liver Physiology. 2017;312[1]:G52-G62.

375. Preidis GA, Ajami NJ, Wong MC, Bessard BC, Conner ME, Petrosino JF. Composition and function of the undernourished neonatal mouse intestinal microbiome. J Nutr Biochem. 2015;26[10]:1050-7.

376. Zhou Q, Zhang Y, Wang X, Yang R, Zhu X, Zhang Y, et al. Gut bacteria Akkermansia is associated with reduced risk of obesity: evidence from the American Gut Project. Nutr Metab [Lond]. 2020;17:90.

377. Chattopadhyay I, Dhar R, Pethusamy K, Seethy A, Srivastava T, Sah R, et al. Exploring the Role of Gut Microbiome in Colon Cancer. Appl Biochem Biotechnol. 2021;193[6]:1780-99.

378. Kowalski K, Mulak A. Brain-Gut-Microbiota Axis in Alzheimer's Disease. J Neurogastroenterol Motil. 2019;25[1]:48-60.

379. Xu M, Xu X, Li J, Li F. Association Between Gut Microbiota and Autism Spectrum Disorder: A Systematic Review and Meta-Analysis. Front Psychiatry. 2019;10:473.

380. Boertien JM, Pereira PAB, Aho VTE, Scheperjans F. Increasing Comparability and Utility of Gut Microbiome Studies in Parkinson's Disease: A Systematic Review. J Parkinsons Dis. 2019;9[s2]:S297-S312.

381. Wong ML, Inserra A, Lewis MD, Mastronardi CA, Leong L, Choo J, et al. Inflammasome signaling affects anxiety- and depressive-like behavior and gut microbiome composition. Mol Psychiatry. 2016;21[6]:797-805.

382. Mashaqi S, Kallamadi R, Matta A, Quan SF, Patel SI, Combs D, et al. Obstructive Sleep Apnea as a Risk Factor for COVID-19 Severity-The Gut Microbiome as a Common Player Mediating Systemic Inflammation via Gut Barrier Dysfunction. Cells. 2022;11[9].

383. Zhu L, Sha L, Li K, Wang Z, Wang T, Li Y, et al. Dietary flaxseed oil rich in omega-3 suppresses severity of type 2 diabetes mellitus via anti-inflammation and modulating gut microbiota in rats. Lipids Health Dis. 2020;19[1]:20.

384. Saulnier DM, Riehle K, Mistretta TA, Diaz MA, Mandal D, Raza S, et al. Gastrointestinal microbiome signatures of pediatric patients with irritable bowel syndrome. Gastroenterology. 2011;141[5]:1782-91.

385. Kang Y, Li Y, Du Y, Guo L, Chen M, Huang X, et al. Konjaku flour reduces obesity in mice by modulating the composition of the gut microbiota. Int J Obes [Lond]. 2019;43[8]:1631-43.

386. Tilg H, Adolph TE, Gerner RR, Moschen AR. The Intestinal Microbiota in Colorectal Cancer. Cancer Cell. 2018;33[6]:954-64.

387. Santoru ML, Piras C, Murgia A, Palmas V, Camboni T, Liggi S, et al. Cross sectional evaluation of the gut-microbiome metabolome axis in an Italian cohort of IBD patients. Sci Rep. 2017;7[1]:9523.

388. Phan J, Nair D, Jain S, Montagne T, Flores DV, Nguyen A, et al. Alterations in gut microbiome composition and function in irritable bowel syndrome and increased probiotic abundance with daily supplementation. Msystems. 2021;6[6]:e01215-21.

389. Nistal E, Saenz de Miera LE, Ballesteros Pomar M, Sanchez-Campos S, Garcia-Mediavilla MV, Alvarez-Cuenllas B, et al. An altered fecal microbiota profile in patients with non-alcoholic fatty liver disease [NAFLD] associated with obesity. Rev Esp Enferm Dig. 2019;111[4]:275-82.

390. Finegold SM, Dowd SE, Gontcharova V, Liu C, Henley KE, Wolcott RD, et al. Pyrosequencing study of fecal microflora of autistic and control children. Anaerobe. 2010;16[4]:444-53.

391. Yildiz S, Dogan I, Dogruman-Al F, Nalbantoglu U, Ustek D, Sarzhanov F, et al. Association of Enteric Protist Blastocystis spp. and Gut Microbiota with Hepatic Encephalopathy. J Gastrointestin Liver Dis. 2016;25[4]:489-97.

392. Ko CY, Liu QQ, Su HZ, Zhang HP, Fan JM, Yang JH, et al. Gut microbiota in obstructive sleep apnea-hypopnea syndrome: disease-related dysbiosis and metabolic comorbidities. Clin Sci [Lond]. 2019;133[7]:905-17.

393. Sato J, Kanazawa A, Ikeda F, Yoshihara T, Goto H, Abe H, et al. Gut dysbiosis and detection of "live gut bacteria" in blood of Japanese patients with type 2 diabetes. Diabetes Care. 2014;37[8]:2343-50.

394. Takaishi H, Matsuki T, Nakazawa A, Takada T, Kado S, Asahara T, et al. Imbalance in intestinal microflora constitution could be involved in the pathogenesis of inflammatory bowel disease. Int J Med Microbiol. 2008;298[5-6]:463-72.

395. Tana C, Umesaki Y, Imaoka A, Handa T, Kanazawa M, Fukudo S. Altered profiles of intestinal microbiota and organic acids may be the origin of symptoms in irritable bowel syndrome. Neurogastroenterol Motil. 2010;22[5]:512-e115.

396. Valentine GC, Hair AB, Martin CR. Microbiome and pediatric obesity, malnutrition, and nutrition. The Developing Microbiome. 2020:157-81.

397. Minato T, Maeda T, Fujisawa Y, Tsuji H, Nomoto K, Ohno K, et al. Progression of Parkinson's disease is associated with gut dysbiosis: Two-year follow-up study. PLoS One. 2017;12[11]:e0187307.

398. Chen Y, Ji F, Guo J, Shi D, Fang D, Li L. Dysbiosis of small intestinal microbiota in liver cirrhosis and its association with etiology. Sci Rep. 2016;6:34055.

399. Wang J, Li W, Wang C, Wang L, He T, Hu H, et al. Enterotype Bacteroides Is Associated with a High Risk in Patients with Diabetes: A Pilot Study. J Diabetes Res. 2020;2020:6047145.

400. Zhou Y, Zhi F. Lower Level of Bacteroides in the Gut Microbiota Is Associated with Inflammatory Bowel Disease: A Meta-Analysis. Biomed Res Int. 2016;2016:5828959.

401. Noor SO, Ridgway K, Scovell L, Kemsley EK, Lund EK, Jamieson C, et al. Ulcerative colitis and irritable bowel patients exhibit distinct abnormalities of the gut microbiota. BMC Gastroenterol. 2010;10[1]:1-9.

402. Pekmez CT, Dragsted LO, Brahe LK. Gut microbiota alterations and dietary modulation in childhood malnutrition–the role of short chain fatty acids. Clin Nutr. 2019;38[2]:615-30.

403. Dong TS, Guan M, Mayer EA, Stains J, Liu C, Vora P, et al. Obesity is associated with a distinct brain-gut microbiome signature that connects Prevotella and Bacteroides to the brain's reward center. Gut Microbes. 2022;14[1]:2051999.

404. Ahmed SA, Elhefnawy AM, Azouz HG, Roshdy YS, Ashry MH, Ibrahim AE, et al. Study of the gut Microbiome Profile in Children with Autism Spectrum Disorder: a Single Tertiary Hospital Experience. J Mol Neurosci. 2020;70[6]:887-96.

405. Zhang Y, Fan Q, Hou Y, Zhang X, Yin Z, Cai X, et al. Bacteroides species differentially modulate depression-like behavior via gut-brain metabolic signaling. Brain Behav Immun. 2022;102:11-22.

406. Magzal F, Shochat T, Haimov I, Tamir S, Asraf K, Tuchner-Arieli M, et al. Increased physical activity improves gut microbiota composition and reduces short-chain fatty acid concentrations in older adults with insomnia. Sci Rep. 2022;12[1]:2265.

407. Wei X, Tao J, Xiao S, Jiang S, Shang E, Zhu Z, et al. Xiexin Tang improves the symptom of type 2 diabetic rats by modulation of the gut microbiota. Sci Rep. 2018;8[1]:3685.

408. Liu C, Zhan S, Tian Z, Li N, Li T, Wu D, et al. Food Additives Associated with Gut Microbiota Alterations in Inflammatory Bowel Disease: Friends or Enemies? Nutrients. 2022;14[15].

409. Valdez-Palomares F, Nambo-Venegas R, Uribe-García J, Mendoza-Vargas A, Granados-Portillo O, Meraz-Cruz N, et al. Intestinal microbiota fingerprint in subjects with irritable bowel syndrome responders to a low FODMAP diet. Food Funct. 2021;12[7]:3206-18.

410. Zhong H, Abdullah, Deng L, Zhao M, Tang J, Liu T, et al. Probiotic-fermented blueberry juice prevents obesity and hyperglycemia in high fat diet-fed mice in association with modulating the gut microbiota. Food Funct. 2020;11[10]:9192-207.

411. Sarhadi V, Lahti L, Saberi F, Youssef O, Kokkola A, Karla T, et al. Gut Microbiota and Host Gene Mutations in Colorectal Cancer Patients and Controls of Iranian and Finnish Origin. Anticancer Res. 2020;40[3]:1325-34.

412. D'Argenio V, Veneruso I, Gong C, Cecarini V, Bonfili L, Eleuteri AM. Gut Microbiome and Mycobiome Alterations in an In Vivo Model of Alzheimer's Disease. Genes [Basel]. 2022;13[9].

413. Chi L, Khan I, Lin Z, Zhang J, Lee MYS, Leong W, et al. Fructo-oligosaccharides from Morinda officinalis remodeled gut microbiota and alleviated depression features in a stress rat model. Phytomedicine. 2020;67:153157.

414. Wang MW, Ma WJ, Wang Y, Ma XH, Xue YF, Guan J, et al. Comparison of the effects of probiotics, rifaximin, and lactulose in the treatment of minimal hepatic encephalopathy and gut microbiota. Front Microbiol. 2023;14:1091167.

415. Le KA, Li Y, Xu X, Yang W, Liu T, Zhao X, et al. Alterations in fecal Lactobacillus and Bifidobacterium species in type 2 diabetic patients in Southern China population. Front Physiol. 2012;3:496.

416. Fava F, Danese S. Intestinal microbiota in inflammatory bowel disease: friend of foe? World J Gastroenterol. 2011;17[5]:557-66.

417. Iddrisu I, Monteagudo-Mera A, Poveda C, Pyle S, Shahzad M, Andrews S, et al. Malnutrition and gut microbiota in children. Nutrients. 2021;13[8]:2727.

418. Million M, Maraninchi M, Henry M, Armougom F, Richet H, Carrieri P, et al. Obesity-associated gut microbiota is enriched in Lactobacillus reuteri and depleted in Bifidobacterium animalis and Methanobrevibacter smithii. Int J Obes [Lond]. 2012;36[6]:817-25.

419. Zou S, Fang L, Lee MH. Dysbiosis of gut microbiota in promoting the development of colorectal cancer. Gastroenterol Rep [Oxf]. 2018;6[1]:1-12.

420. Aho VTE, Pereira PAB, Voutilainen S, Paulin L, Pekkonen E, Auvinen P, et al. Gut microbiota in Parkinson's disease: Temporal stability and relations to disease progression. EBioMedicine. 2019;44:691-707.

421. Aizawa E, Tsuji H, Asahara T, Takahashi T, Teraishi T, Yoshida S, et al. Possible association of Bifidobacterium and Lactobacillus in the gut microbiota of patients with major depressive disorder. J Affect Disord. 2016;202:254-7.

422. Xia X, Chen J, Xia J, Wang B, Liu H, Yang L, et al. Role of probiotics in the treatment of minimal hepatic encephalopathy in patients with HBV-induced liver cirrhosis. J Int Med Res. 2018;46[9]:3596-604.

423. Liu G, Liang L, Yu G, Li Q. Pumpkin polysaccharide modifies the gut microbiota during alleviation of type 2 diabetes in rats. Int J Biol Macromol. 2018;115:711-7.

424. Berry D, Reinisch W. Intestinal microbiota: a source of novel biomarkers in inflammatory bowel diseases? Best Pract Res Clin Gastroenterol. 2013;27[1]:47-58.

425. Min YW, Rezaie A, Pimentel M. Bile acid and gut microbiota in irritable bowel syndrome. J Neurogastroenterol Motil. 2022;28[4]:549-61.

426. Araujo JR, Tomas J, Brenner C, Sansonetti PJ. Impact of high-fat diet on the intestinal microbiota and small intestinal physiology before and after the onset of obesity. Biochimie. 2017;141:97-106.

427. Ai D, Pan H, Li X, Gao Y, Liu G, Xia LC. Identifying Gut Microbiota Associated With Colorectal Cancer Using a Zero-Inflated Lognormal Model. Front Microbiol. 2019;10:826.

428. Baldini F, Hertel J, Sandt E, Thinnes CC, Neuberger-Castillo L, Pavelka L, et al. Parkinson's disease-associated alterations of the gut microbiome predict disease-relevant changes in metabolic functions. BMC Biol. 2020;18[1]:62.

429. Caso JR, MacDowell KS, Gonzalez-Pinto A, Garcia S, de Diego-Adelino J, Carceller-Sindreu M, et al. Gut microbiota, innate immune pathways, and inflammatory control mechanisms in patients with major depressive disorder. Transl Psychiatry. 2021;11[1]:645.

430. Diaz-Perdigones CM, Munoz-Garach A, Alvarez-Bermudez MD, Moreno-Indias I, Tinahones FJ. Gut microbiota of patients with type 2 diabetes and gastrointestinal intolerance to metformin differs in composition and functionality from tolerant patients. Biomed Pharmacother. 2022;145:112448.

431. Sokol H, Jegou S, McQuitty C, Straub M, Leducq V, Landman C, et al. Specificities of the intestinal microbiota in patients with inflammatory bowel disease and Clostridium difficile infection. Gut Microbes. 2018;9[1]:55-60.

432. Kamil RZ, Murdiati A, Juffrie M, Nakayama J, Rahayu ES. Gut microbiota and short-chain fatty acid profile between normal and moderate malnutrition children in Yogyakarta, Indonesia. Microorganisms. 2021;9[1]:127.

433. Zhong X, Harrington JM, Millar SR, Perry IJ, O'Toole PW, Phillips CM. Gut microbiota associations with metabolic health and obesity status in older adults. Nutrients. 2020;12[8]:2364.

434. Ni JJ, Li XS, Zhang H, Xu Q, Wei XT, Feng GJ, et al. Mendelian randomization study of causal link from gut microbiota to colorectal cancer. BMC Cancer. 2022;22[1]:1371.

435. Li W, Wu X, Hu X, Wang T, Liang S, Duan Y, et al. Structural changes of gut microbiota in Parkinson's disease and its correlation with clinical features. Sci China Life Sci. 2017;60[11]:1223-33.

436. Shen W, Tao Y, Zheng F, Zhou H, Wu H, Shi H, et al. The alteration of gut microbiota in venlafaxine-ameliorated chronic unpredictable mild stress-induced depression in mice. Behav Brain Res. 2023;446:114399.

437. Van Meijel RLJ, Venema K, Canfora EE, Blaak EE, Goossens GH. Mild intermittent hypoxia exposure alters gut microbiota composition in men with overweight and obesity. Benef Microbes. 2022;13[4]:355-63.

438. Alvarez-Silva C, Kashani A, Hansen TH, Pinna NK, Anjana RM, Dutta A, et al. Trans-ethnic gut microbiota signatures of type 2 diabetes in Denmark and India. Genome Med. 2021;13[1]:37.

439. Eeckhaut V, Machiels K, Perrier C, Romero C, Maes S, Flahou B, et al. Butyricicoccus pullicaecorum in inflammatory bowel disease. Gut. 2013;62[12]:1745-52.

440. Enqi W, Jingzhu S, Lingpeng P, Yaqin L. Comparison of the gut microbiota disturbance in rat models of irritable bowel syndrome induced by maternal separation and multiple early-life adversity. Frontiers in Cellular and Infection Microbiology. 2021;10:581974.

441. Yang M, Yin Y, Wang F, Zhang H, Ma X, Yin Y, et al. Supplementation With Lycium barbarum Polysaccharides Reduce Obesity in High-Fat Diet-Fed Mice by Modulation of Gut Microbiota. Front Microbiol. 2021;12:719967.

442. Huang M, Liu K, Wei Z, Feng Z, Chen J, Yang J, et al. Serum Oxytocin Level Correlates With Gut Microbiome Dysbiosis in Children With Autism Spectrum Disorder. Front Neurosci. 2021;15:721884.

443. Qian Y, Yang X, Xu S, Wu C, Song Y, Qin N, et al. Alteration of the fecal microbiota in Chinese patients with Parkinson's disease. Brain Behav Immun. 2018;70:194-202.

444. Zhang Y-J, Li S, Gan R-Y, Zhou T, Xu D-P, Li H-B. Impacts of gut bacteria on human health and diseases. Int J Mol Sci. 2015;16[4]:7493-519.

445. Radwan S, Gilfillan D, Eklund B, Radwan HM, El Menofy NG, Lee J, et al. A comparative study of the gut microbiome in Egyptian patients with Type I and Type II diabetes. PLoS One. 2020;15[9]:e0238764.

446. Gallardo-Becerra L, Cornejo-Granados F, Garcia-Lopez R, Valdez-Lara A, Bikel S, Canizales-Quinteros S, et al. Metatranscriptomic analysis to define the Secrebiome, and 16S rRNA profiling of the gut microbiome in obesity and metabolic syndrome of Mexican children. Microb Cell Fact. 2020;19[1]:61.

447. Cammann D, Lu Y, Cummings MJ, Zhang ML, Cue JM, Do J, et al. Genetic correlations between Alzheimer's disease and gut microbiome genera. Sci Rep. 2023;13[1]:5258.

448. Wu T, Wang H, Lu W, Zhai Q, Zhang Q, Yuan W, et al. Potential of gut microbiome for detection of autism spectrum disorder. Microb Pathog. 2020;149:104568.

449. Zhang P, Huang P, Du J, He Y, Liu J, He G, et al. Specific gut microbiota alterations in essential tremor and its difference from Parkinson's disease. NPJ Parkinsons Dis. 2022;8[1]:98.

450. Philips CA, Augustine P, Ganesan K, Ranade S, Chopra V, Patil K, et al. The role of gut microbiota in clinical complications, disease severity, and treatment response in severe alcoholic hepatitis. Indian J Gastroenterol. 2022;41[1]:37-51.

451. Bien J, Palagani V, Bozko P. The intestinal microbiota dysbiosis and Clostridium difficile infection: is there a relationship with inflammatory bowel disease? Therap Adv Gastroenterol. 2013;6[1]:53-68.

452. Salonen A, de Vos WM, Palva A. Gastrointestinal microbiota in irritable bowel syndrome: present state and perspectives. Microbiology. 2010;156[11]:3205-15.

453. Gomes AC, Hoffmann C, Mota JF. The human gut microbiota: Metabolism and perspective in obesity. Gut Microbes. 2018;9[4]:308-25.

454. Sun J, Xu J, Yang B, Chen K, Kong Y, Fang N, et al. Effect of Clostridium butyricum against Microglia-Mediated Neuroinflammation in Alzheimer's Disease via Regulating Gut Microbiota and Metabolites Butyrate. Mol Nutr Food Res. 2020;64[2]:e1900636.

455. Parracho HM, Bingham MO, Gibson GR, McCartney AL. Differences between the gut microflora of children with autistic spectrum disorders and that of healthy children. J Med Microbiol. 2005;54[Pt 10]:987-91.

456. Palacios N, Hannoun A, Flahive J, Ward D, Goostrey K, Deb A, et al. Effect of Levodopa Initiation on the Gut Microbiota in Parkinson's Disease. Front Neurol. 2021;12:574529.

457. Winter G, Hart RA, Charlesworth RPG, Sharpley CF. Gut microbiome and depression: what we know and what we need to know. Rev Neurosci. 2018;29[6]:629-43.

458. Afolayan A, Adebusoye L, Cadmus E, Ayeni F. Insights into the gut microbiota of Nigerian elderly with type 2 diabetes and non-diabetic elderly persons. Heliyon. 2020;6[5]:e03971.

459. Chen D, Chen G, Chen C, Zeng X, Ye H. Prebiotics effects in vitro of polysaccharides from tea flowers on gut microbiota of healthy persons and patients with inflammatory bowel disease. Int J Biol Macromol. 2020;158:968-76.

460. Malinen E, Krogius-Kurikka L, Lyra A, Nikkilä J, Jääskeläinen A, Rinttilä T, et al. Association of symptoms with gastrointestinal microbiota in irritable bowel syndrome. World journal of gastroenterology: WJG. 2010;16[36]:4532.

461. Gatya M, Fibri DLN, Utami T, Suroto DA, Rahayu ES. Gut Microbiota Composition in Undernourished Children Associated with Diet and Sociodemographic Factors: A Case–Control Study in Indonesia. Microorganisms. 2022;10[9]:1748.

462. Gomez-Arango LF, Barrett HL, Wilkinson SA, Callaway LK, McIntyre HD, Morrison M, et al. Low dietary fiber intake increases Collinsella abundance in the gut microbiota of overweight and obese pregnant women. Gut microbes. 2018;9[3]:189-201.

463. Sheng Q, Du H, Cheng X, Cheng X, Tang Y, Pan L, et al. Characteristics of fecal gut microbiota in patients with colorectal cancer at different stages and different sites. Oncol Lett. 2019;18[5]:4834-44.

464. Huang B, Chau SWH, Liu Y, Chan JWY, Wang J, Ma SL, et al. Gut microbiome dysbiosis across early Parkinson's disease, REM sleep behavior disorder and their first-degree relatives. Nat Commun. 2023;14[1]:2501.

465. Yu X, Jin Y, Zhou W, Xiao T, Wu Z, Su J, et al. Rifaximin Modulates the Gut Microbiota to Prevent Hepatic Encephalopathy in Liver Cirrhosis Without Impacting the Resistome. Front Cell Infect Microbiol. 2021;11:761192.

466. Durgan DJ. Obstructive Sleep Apnea-Induced Hypertension: Role of the Gut Microbiota. Curr Hypertens Rep. 2017;19[4]:35.

467. Huang X, Qiu Y, Gao Y, Zhou R, Hu Q, He Z, et al. Gut microbiota mediate melatonin signalling in association with type 2 diabetes. Diabetologia. 2022;65[10]:1627-41.

468. Scher JU, Ubeda C, Artacho A, Attur M, Isaac S, Reddy SM, et al. Decreased bacterial diversity characterizes the altered gut microbiota in patients with psoriatic arthritis, resembling dysbiosis in inflammatory bowel disease. Arthritis & rheumatology. 2015;67[1]:128-39.

469. Monteleone AM, Troisi J, Fasano A, Dalle Grave R, Marciello F, Serena G, et al. Multi-omics data integration in anorexia nervosa patients before and after weight regain: A microbiome-metabolomics investigation. Clin Nutr. 2021;40[3]:1137-46.

470. Kasai C, Sugimoto K, Moritani I, Tanaka J, Oya Y, Inoue H, et al. Comparison of the gut microbiota composition between obese and non-obese individuals in a Japanese population, as analyzed by terminal restriction fragment length polymorphism and next-generation sequencing. BMC Gastroenterol. 2015;15:100.

471. Andreo-Martinez P, Garcia-Martinez N, Sanchez-Samper EP, Martinez-Gonzalez AE. An approach to gut microbiota profile in children with autism spectrum disorder. Environ Microbiol Rep. 2020;12[2]:115-35.

472. Radjabzadeh D, Bosch JA, Uitterlinden AG, Zwinderman AH, Ikram MA, van Meurs JBJ, et al. Gut microbiome-wide association study of depressive symptoms. Nat Commun. 2022;13[1]:7128.

473. Mashaqi S, Gozal D. Obstructive Sleep Apnea and Systemic Hypertension: Gut Dysbiosis as the Mediator? J Clin Sleep Med. 2019;15[10]:1517-27.

474. Gong X, Xiong L, Bi C, Zhang B. Diosmetin ameliorate type 2 diabetic mellitus by up-regulating Corynebacterium glutamicum to regulate IRS/PI3K/AKT-mediated glucose metabolism disorder in KK-Ay mice. Phytomedicine. 2021;87:153582.

475. Takaishi H, Matsuki T, Nakazawa A, Takada T, Kado S, Asahara T, et al. Imbalance in intestinal microflora constitution could be involved in the pathogenesis of inflammatory bowel disease. Int J Med Microbiol. 2008;298[5-6]:463-72.

476. Gracey M, Suharjono S, Sunoto S, Stone DE. Microbial Contamination of the Gut; another Feature of Malnutrition. Paediatr Indones. 1975;15[7-8]:181-90.

477. Lo CI, Niang EHA, Ndongo S, Raoult D, Fournier PE, Fenollar F. Corynebacterium bouchesdurhonense sp. nov., and Corynebacterium provencense sp. nov., two new species isolated from obese patients. New Microbes New Infect. 2019;31:100581.

478. Hasan R, Bose S, Roy R, Paul D, Rawat S, Nilwe P, et al. Tumor tissue-specific bacterial biomarker panel for colorectal cancer: Bacteroides massiliensis, Alistipes species, Alistipes onderdonkii, Bifidobacterium pseudocatenulatum, Corynebacterium appendicis. Arch Microbiol. 2022;204[6]:348.

479. Chang CH, Lin CH, Lane HY. d-glutamate and Gut Microbiota in Alzheimer's Disease. Int J Mol Sci. 2020;21[8].

480. Aitbali Y, Ba-M'hamed S, Elhidar N, Nafis A, Soraa N, Bennis M. Glyphosate based- herbicide exposure affects gut microbiota, anxiety and depression-like behaviors in mice. Neurotoxicol Teratol. 2018;67:44-9.

481. Kawaguchi T, Suzuki F, Imamura M, Murashima N, Yanase M, Mine T, et al. Rifaximin-altered gut microbiota components associated with liver/neuropsychological functions in patients with hepatic encephalopathy: An exploratory data analysis of phase II/III clinical trials. Hepatol Res. 2019;49[4]:404-18.

482. Nie Q, Hu J, Chen H, Geng F, Nie S. Arabinoxylan ameliorates type 2 diabetes by regulating the gut microbiota and metabolites. Food Chem. 2022;371:131106.

483. Gobert AP, Sagrestani G, Delmas E, Wilson KT, Verriere TG, Dapoigny M, et al. The human intestinal microbiota of constipated-predominant irritable bowel syndrome patients exhibits anti-inflammatory properties. Sci Rep. 2016;6[1]:39399.

484. Karlsson CL, Onnerfalt J, Xu J, Molin G, Ahrne S, Thorngren-Jerneck K. The microbiota of the gut in preschool children with normal and excessive body weight. Obesity [Silver Spring]. 2012;20[11]:2257-61.

485. Li C, Wang N, Zheng G, Yang L. Oral Administration of Resveratrol-Selenium-Peptide Nanocomposites Alleviates Alzheimer's Disease-like Pathogenesis by Inhibiting Abeta Aggregation and Regulating Gut Microbiota. ACS Appl Mater Interfaces. 2021;13[39]:46406-20.

486. Heberling CA, Dhurjati PS, Sasser M. Hypothesis for a systems connectivity model of Autism Spectrum Disorder pathogenesis: links to gut bacteria, oxidative stress, and intestinal permeability. Med Hypotheses. 2013;80[3]:264-70.

487. Murros KE, Huynh VA, Takala TM, Saris PEJ. Desulfovibrio Bacteria Are Associated With Parkinson's Disease. Front Cell Infect Microbiol. 2021;11:652617.

488. Liu L, Wang H, Zhang H, Chen X, Zhang Y, Wu J, et al. Toward a Deeper Understanding of Gut Microbiome in Depression: The Promise of Clinical Applicability. Adv Sci [Weinh]. 2022;9[35]:e2203707.

489. Giuffre M, Campigotto M, Campisciano G, Comar M, Croce LS. A story of liver and gut microbes: how does the intestinal flora affect liver disease? A review of the literature. Am J Physiol Gastrointest Liver Physiol. 2020;318[5]:G889-G906.

490. Elbere I, Silamikelis I, Dindune, II, Kalnina I, Ustinova M, Zaharenko L, et al. Baseline gut microbiome composition predicts metformin therapy short-term efficacy in newly diagnosed type 2 diabetes patients. PLoS One. 2020;15[10]:e0241338.

491. Lopetuso LR, Petito V, Graziani C, Schiavoni E, Sterbini FP, Poscia A, et al. Gut microbiota in health, diverticular disease, irritable bowel syndrome, and inflammatory bowel diseases: time for microbial marker of gastrointestinal disorders. Dig Dis. 2018;36[1]:56-65.

492. Chumpitazi BP, Hollister EB, Oezguen N, Tsai CM, McMeans AR, Luna RA, et al. Gut microbiota influences low fermentable substrate diet efficacy in children with irritable bowel syndrome. Gut microbes. 2014;5[2]:165-75.

493. Toe LC, Kerckhof F-M, De Bodt J, Morel FB, Ouedraogo J-B, Kolsteren P, et al. A prebiotic-enhanced lipid-based nutrient supplement [LNSp] increases Bifidobacterium relative abundance and enhances short-chain fatty acid production in simulated colonic microbiota from undernourished infants. FEMS Microbiol Ecol. 2020;96[7]:fiaa105.

494. Naderpoor N, Mousa A, Gomez-Arango LF, Barrett HL, Dekker Nitert M, de Courten B. Faecal Microbiota Are Related to Insulin Sensitivity and Secretion in Overweight or Obese Adults. J Clin Med. 2019;8[4].

495. Huo RX, Wang YJ, Hou SB, Wang W, Zhang CZ, Wan XH. Gut mucosal microbiota profiles linked to colorectal cancer recurrence. World J Gastroenterol. 2022;28[18]:1946-64.

496. Chiang HL, Lin CH. Altered Gut Microbiome and Intestinal Pathology in Parkinson's Disease. J Mov Disord. 2019;12[2]:67-83.

497. Valles-Colomer M, Falony G, Darzi Y, Tigchelaar EF, Wang J, Tito RY, et al. The neuroactive potential of the human gut microbiota in quality of life and depression. Nat Microbiol. 2019;4[4]:623-32.

498. Li Q, Chang Y, Zhang K, Chen H, Tao S, Zhang Z. Implication of the gut microbiome composition of type 2 diabetic patients from northern China. Sci Rep. 2020;10[1]:5450.

499. Blanton LV, Charbonneau MR, Salih T, Barratt MJ, Venkatesh S, Ilkaveya O, et al. Gut bacteria that rescue growth impairments transmitted by immature microbiota from undernourished children. Science [New York, NY]. 2016;351[6275].

500. Jiao N, Baker SS, Nugent CA, Tsompana M, Cai L, Wang Y, et al. Gut microbiome may contribute to insulin resistance and systemic inflammation in obese rodents: a meta-analysis. Physiol Genomics. 2018;50[4]:244-54.

501. Li B, He Y, Ma J, Huang P, Du J, Cao L, et al. Mild cognitive impairment has similar alterations as Alzheimer's disease in gut microbiota. Alzheimers Dement. 2019;15[10]:1357-66.

502. Nishiwaki H, Ito M, Hamaguchi T, Maeda T, Kashihara K, Tsuboi Y, et al. Short chain fatty acids-producing and mucin-degrading intestinal bacteria predict the progression of early Parkinson's disease. NPJ Parkinsons Dis. 2022;8[1]:65.

503. Zhang Q, Yun Y, An H, Zhao W, Ma T, Wang Z, et al. Gut Microbiome Composition Associated With Major Depressive Disorder and Sleep Quality. Front Psychiatry. 2021;12:645045.

504. Bajaj JS, Hylemon PB, Ridlon JM, Heuman DM, Daita K, White MB, et al. Colonic mucosal microbiome differs from stool microbiome in cirrhosis and hepatic encephalopathy and is linked to cognition and inflammation. Am J Physiol Gastrointest Liver Physiol. 2012;303[6]:G675-85.

505. Tang SS, Liang CH, Liu YL, Wei W, Deng XR, Shi XY, et al. Intermittent hypoxia is involved in gut microbial dysbiosis in type 2 diabetes mellitus and obstructive sleep apnea-hypopnea syndrome. World J Gastroenterol. 2022;28[21]:2320-33.

506. Qin J, Li Y, Cai Z, Li S, Zhu J, Zhang F, et al. A metagenome-wide association study of gut microbiota in type 2 diabetes. Nature. 2012;490[7418]:55-60.

507. Zhan K, Zheng H, Li J, Wu H, Qin S, Luo L, et al. Gut microbiota-bile acid crosstalk in diarrhea-irritable bowel syndrome. BioMed Research International. 2020;2020.

508. Bai J, Shen N, Liu Y. Associations between the Gut Microbiome and Migraines in Children Aged 7-18 Years: An Analysis of the American Gut Project Cohort. Pain Manag Nurs. 2023;24[1]:35-43.

509. Pinart M, Dotsch A, Schlicht K, Laudes M, Bouwman J, Forslund SK, et al. Gut Microbiome Composition in Obese and Non-Obese Persons: A Systematic Review and Meta-Analysis. Nutrients. 2021;14[1].

510. Bai X, Wei H, Liu W, Coker OO, Gou H, Liu C, et al. Cigarette smoke promotes colorectal cancer through modulation of gut microbiota and related metabolites. Gut. 2022;71[12]:2439-50.

511. Wang M, Wan J, Rong H, He F, Wang H, Zhou J, et al. Alterations in Gut Glutamate Metabolism Associated with Changes in Gut Microbiota Composition in Children with Autism Spectrum Disorder. mSystems. 2019;4[1].

512. Lubomski M, Davis RL, Sue CM. The gut microbiota: A novel therapeutic target in Parkinson's disease? Parkinsonism Relat Disord. 2019;66:265-6.

513. Philips CA, Ahamed R, Rajesh S, Singh S, Tharakan A, Abduljaleel JK, et al. Clinical outcomes and gut microbiota analysis of severe alcohol-associated hepatitis patients undergoing healthy donor fecal transplant or pentoxifylline therapy: single-center experience from Kerala. Gastroenterol Rep [Oxf]. 2022;10:goac074.

514. Xu T, Ge Y, Du H, Li Q, Xu X, Yi H, et al. Berberis kansuensis extract alleviates type 2 diabetes in rats by regulating gut microbiota composition. J Ethnopharmacol. 2021;273:113995.

515. Balish E, Warner T. Enterococcus faecalis induces inflammatory bowel disease in interleukin-10 knockout mice. The American journal of pathology. 2002;160[6]:2253-7.

516. Ji M, Huang H, Lan X. Correlation between intestinal microflora in irritable bowel syndrome and severity. Dis Markers. 2022;2022.

517. Lv Z, Wang Y, Yang T, Zhan X, Li Z, Hu H, et al. Vitamin A deficiency impacts the structural segregation of gut microbiota in children with persistent diarrhea. J Clin Biochem Nutr. 2016;59[2]:113-21.

518. Wu GD, Pan A, Zhang X, Cai YY, Wang Q, Huang FQ, et al. Cordyceps Improves Obesity and its Related Inflammation via Modulation of Enterococcus cecorum Abundance and Bile Acid Metabolism. Am J Chin Med. 2022;50[3]:817-38.

519. de Almeida CV, Taddei A, Amedei A. The controversial role of Enterococcus faecalis in colorectal cancer. Therap Adv Gastroenterol. 2018;11:1756284818783606.

520. Fan HX, Sheng S, Zhang F. New hope for Parkinson's disease treatment: Targeting gut microbiota. CNS Neurosci Ther. 2022;28[11]:1675-88.

521. Jang HM, Kim JK, Joo MK, Shin YJ, Lee KE, Lee CK, et al. Enterococcus faecium and Pediococcus acidilactici deteriorate Enterobacteriaceae-induced depression and colitis in mice. Sci Rep. 2022;12[1]:9389.

522. Loguercio C, Del Vecchio Blanco C, Coltorti M. Enterococcus lactic acid bacteria strain SF68 and lactulose in hepatic encephalopathy: a controlled study. J Int Med Res. 1987;15[6]:335-43.

523. Forslund K, Hildebrand F, Nielsen T, Falony G, Le Chatelier E, Sunagawa S, et al. Disentangling type 2 diabetes and metformin treatment signatures in the human gut microbiota. Nature. 2015;528[7581]:262-6.

524. Mirsepasi-Lauridsen HC, Vallance BA, Krogfelt KA, Petersen AM. Escherichia coli pathobionts associated with inflammatory bowel disease. Clin Microbiol Rev. 2019;32[2]:e00060-18.

525. Lee KN, Lee OY. Intestinal microbiota in pathophysiology and management of irritable bowel syndrome. World journal of gastroenterology: WJG. 2014;20[27]:8886.

526. Million M, Angelakis E, Maraninchi M, Henry M, Giorgi R, Valero R, et al. Correlation between body mass index and gut concentrations of Lactobacillus reuteri, Bifidobacterium animalis, Methanobrevibacter smithii and Escherichia coli. Int J Obes [Lond]. 2013;37[11]:1460-6.

527. Radilla-Vazquez RB, Parra-Rojas I, Martinez-Hernandez NE, Marquez-Sandoval YF, Illades-Aguiar B, Castro-Alarcon N. Gut Microbiota and Metabolic Endotoxemia in Young Obese Mexican Subjects. Obes Facts. 2016;9[1]:1-11.

528. Bostanciklioglu M. The role of gut microbiota in pathogenesis of Alzheimer's disease. J Appl Microbiol. 2019;127[4]:954-67.

529. Carissimi C, Laudadio I, Palone F, Fulci V, Cesi V, Cardona F, et al. Functional analysis of gut microbiota and immunoinflammation in children with autism spectrum disorders. Dig Liver Dis. 2019;51[10]:1366-74.

530. Sun MF, Shen YQ. Dysbiosis of gut microbiota and microbial metabolites in Parkinson's Disease. Ageing Res Rev. 2018;45:53-61.

531. Du Toit A. Gut microbiota and depression. Nat Rev Microbiol. 2022;20[4]:190.

532. Liu Q, Duan ZP, Ha DK, Bengmark S, Kurtovic J, Riordan SM. Synbiotic modulation of gut flora: effect on minimal hepatic encephalopathy in patients with cirrhosis. Hepatology. 2004;39[5]:1441-9.

533. Bhute SS, Suryavanshi MV, Joshi SM, Yajnik CS, Shouche YS, Ghaskadbi SS. Gut Microbial Diversity Assessment of Indian Type-2-Diabetics Reveals Alterations in Eubacteria, Archaea, and Eukaryotes. Front Microbiol. 2017;8:214.

534. Jones RB, Alderete TL, Kim JS, Millstein J, Gilliland FD, Goran MI. High intake of dietary fructose in overweight/obese teenagers associated with depletion of Eubacterium and Streptococcus in gut microbiome. Gut Microbes. 2019;10[6]:712-9.

535. Ryu SW, Kim JS, Oh BS, Choi WJ, Yu SY, Bak JE, et al. Gut Microbiota Eubacterium callanderi Exerts Anti-Colorectal Cancer Activity. Microbiol Spectr. 2022;10[6]:e0253122.

536. Shabbir U, Arshad MS, Sameen A, Oh DH. Crosstalk between Gut and Brain in Alzheimer's Disease: The Role of Gut Microbiota Modulation Strategies. Nutrients. 2021;13[2].

537. Merli M, Iebba V, Giusto M. What is new about diet in hepatic encephalopathy. Metab Brain Dis. 2016;31[6]:1289-94.

538. Navab-Moghadam F, Sedighi M, Khamseh ME, Alaei-Shahmiri F, Talebi M, Razavi S, et al. The association of type II diabetes with gut microbiota composition. Microb Pathog. 2017;110:630-6.

539. Cao Y, Shen J, Ran ZH. Association between Faecalibacterium prausnitzii reduction and inflammatory bowel disease: a meta-analysis and systematic review of the literature. Gastroenterol Res Pract. 2014;2014.

540. Maharshak N, Ringel Y, Katibian D, Lundqvist A, Sartor RB, Carroll IM, et al. Fecal and mucosa-associated intestinal microbiota in patients with diarrhea-predominant irritable bowel syndrome. Dig Dis Sci. 2018;63:1890-9.

541. Maioli TU, Borras-Nogues E, Torres L, Barbosa SC, Martins VD, Langella P, et al. Possible Benefits of Faecalibacterium prausnitzii for Obesity-Associated Gut Disorders. Front Pharmacol. 2021;12:740636.

542. Dikeocha IJ, Al-Kabsi AM, Chiu HT, Alshawsh MA. Faecalibacterium prausnitzii Ameliorates Colorectal Tumorigenesis and Suppresses Proliferation of HCT116 Colorectal Cancer Cells. Biomedicines. 2022;10[5].

543. Leblhuber F, Ehrlich D, Steiner K, Geisler S, Fuchs D, Lanser L, et al. The Immunopathogenesis of Alzheimer's Disease Is Related to the Composition of Gut Microbiota. Nutrients. 2021;13[2].

544. Hua X, Zhu J, Yang T, Guo M, Li Q, Chen J, et al. The Gut Microbiota and Associated Metabolites Are Altered in Sleep Disorder of Children With Autism Spectrum Disorders. Front Psychiatry. 2020;11:855.

545. Ye X, Wang D, Zhu H, Wang D, Li J, Tang Y, et al. Gut Microbiota Changes in Patients With Major Depressive Disorder Treated With Vortioxetine. Front Psychiatry. 2021;12:641491.

546. Bajaj JS, Gillevet PM, Patel NR, Ahluwalia V, Ridlon JM, Kettenmann B, et al. A longitudinal systems biology analysis of lactulose withdrawal in hepatic encephalopathy. Metab Brain Dis. 2012;27[2]:205-15.

547. Zhang Y, Si X, Yang L, Wang H, Sun Y, Liu N. Association between intestinal microbiota and inflammatory bowel disease. Animal Models and Experimental Medicine. 2022;5[4]:311-22.

548. Wiredu Ocansey DK, Hang S, Yuan X, Qian H, Zhou M, Valerie Olovo C, et al. The diagnostic and prognostic potential of gut bacteria in inflammatory bowel disease. Gut Microbes. 2023;15[1]:2176118.

549. Duan M, Wang Y, Zhang Q, Zou R, Guo M, Zheng H. Characteristics of gut microbiota in people with obesity. PLoS One. 2021;16[8]:e0255446.

550. Kelly D, Yang L, Pei Z. Gut Microbiota, Fusobacteria, and Colorectal Cancer. Diseases. 2018;6[4].

551. Yan C, Diao Q, Zhao Y, Zhang C, He X, Huang R, et al. Fusobacterium nucleatum infection-induced neurodegeneration and abnormal gut microbiota composition in Alzheimer's disease-like rats. Front Neurosci. 2022;16:884543.

552. Talukdar R, Sarkar P, Jakkampudi A, Sarkar S, Aslam M, Jandhyala M, et al. The gut microbiome in pancreatogenic diabetes differs from that of Type 1 and Type 2 diabetes. Sci Rep. 2021;11[1]:10978.

553. Brand EC, Klaassen MA, Gacesa R, Vila AV, Ghosh H, De Zoete MR, et al. Healthy cotwins share gut microbiome signatures with their inflammatory bowel disease twins and unrelated patients. Gastroenterology. 2021;160[6]:1970-85.

554. Selma MV, Romo-Vaquero M, Garcia-Villalba R, Gonzalez-Sarrias A, Tomas-Barberan FA, Espin JC. The human gut microbial ecology associated with overweight and obesity determines ellagic acid metabolism. Food Funct. 2016;7[4]:1769-74.

555. Romo-Vaquero M, Fernandez-Villalba E, Gil-Martinez AL, Cuenca-Bermejo L, Espin JC, Herrero MT, et al. Urolithins: potential biomarkers of gut dysbiosis and disease stage in Parkinson's patients. Food Funct. 2022;13[11]:6306-16.

556. Chen JJ, Zheng P, Liu YY, Zhong XG, Wang HY, Guo YJ, et al. Sex differences in gut microbiota in patients with major depressive disorder. Neuropsychiatr Dis Treat. 2018;14:647-55.

557. Wasilewska J, Klukowski M, Debkowska K, Kilon J, Citko D, Flisiak M, et al. Helicobacter pylori seroprevalence in children with sleep-disordered breathing. Int J Pediatr Otorhinolaryngol. 2016;87:208-12.

558. Hosseininasab Nodoushan SA, Nabavi A. The Interaction of Helicobacter pylori Infection and Type 2 Diabetes Mellitus. Adv Biomed Res. 2019;8:15.

559. Bai X, Jiang L, Ruan G, Liu T, Yang H. Helicobacter pylori may participate in the development of inflammatory bowel disease by modulating the intestinal microbiota. Chin Med J. 2022;135[06]:634-8.

560. Ng QX, Foo NX, Loke W, Koh YQ, Seah VJM, Soh AYS, et al. Is there an association between Helicobacter pylori infection and irritable bowel syndrome? A meta-analysis. World J Gastroenterol. 2019;25[37]:5702.

561. Siddiqui B, Yakoob J, Abbas Z, Azmat R, Fatima SS, Awan S. Distribution of Helicobacter pylori infection and abnormal body- mass index [BMI] in a developing country. J Infect Dev Ctries. 2018;12[5]:342-6.

562. Dai K, Song Y, Zhang D, Wei Y, Jiang S, Xu F, et al. Thinned peach polyphenols alleviate obesity in high fat mice by affecting gut microbiota. Food Res Int. 2022;157:111255.

563. Dahmus JD, Kotler DL, Kastenberg DM, Kistler CA. The gut microbiome and colorectal cancer: a review of bacterial pathogenesis. J Gastrointest Oncol. 2018;9[4]:769-77.

564. Doulberis M, Kotronis G, Gialamprinou D, Polyzos SA, Papaefthymiou A, Katsinelos P, et al. Alzheimer's disease and gastrointestinal microbiota; impact of Helicobacter pylori infection involvement. Int J Neurosci. 2021;131[3]:289-301.

565. Cao X, Lin P, Jiang P, Li C. Characteristics of the gastrointestinal microbiome in children with autism spectrum disorder: a systematic review. Shanghai Arch Psychiatry. 2013;25[6]:342-53.

566. McGee DJ, Lu XH, Disbrow EA. Stomaching the Possibility of a Pathogenic Role for Helicobacter pylori in Parkinson's Disease. J Parkinsons Dis. 2018;8[3]:367-74.

567. Tsai CF, Chen MH, Wang YP, Liu PY, Hou MC, Lee FY, et al. Increased risk of short-term depressive disorder after Helicobacter pylori eradication: A population-based nested cohort study. Helicobacter. 2021;26[4]:e12824.

568. Schulz C, Schutte K, Malfertheiner P. Does H. pylori eradication therapy benefit patients with hepatic encephalopathy?: systematic review. J Clin Gastroenterol. 2014;48[6]:491-9.

569. Lippert K, Kedenko L, Antonielli L, Kedenko I, Gemeier C, Leitner M, et al. Gut microbiota dysbiosis associated with glucose metabolism disorders and the metabolic syndrome in older adults. Beneficial microbes. 2017;8[4]:545-56.

570. Nardone OM, de Sire R, Petito V, Testa A, Villani G, Scaldaferri F, et al. Inflammatory bowel diseases and sarcopenia: the role of inflammation and gut microbiota in the development of muscle failure. Front Immunol. 2021;12:694217.

571. Chumpitazi BP, Hoffman KL, Smith DP, McMeans AR, Musaad S, Versalovic J, et al. Fructan-sensitive children with irritable bowel syndrome have distinct gut microbiome signatures. Aliment Pharmacol Ther. 2021;53[4]:499-509.

572. Kobayashi T, Osaki T, Oikawa S. Use of T-RFLP and seven restriction enzymes to compare the faecal microbiota of obese and lean Japanese healthy men. Benef Microbes. 2015;6[5]:735-45.

573. Jia W, Rajani C, Xu H, Zheng X. Gut microbiota alterations are distinct for primary colorectal cancer and hepatocellular carcinoma. Protein Cell. 2021;12[5]:374-93.

574. Jang JH, Yeom MJ, Ahn S, Oh JY, Ji S, Kim TH, et al. Acupuncture inhibits neuroinflammation and gut microbial dysbiosis in a mouse model of Parkinson's disease. Brain Behav Immun. 2020;89:641-55.

575. Bloom PP, Luevano JM, Jr., Miller KJ, Chung RT. Deep stool microbiome analysis in cirrhosis reveals an association between short-chain fatty acids and hepatic encephalopathy. Ann Hepatol. 2021;25:100333.

576. Tiwana H, Wilson C, Walmsley R, Wakefield A, Smith M, Cox N, et al. Antibody responses to gut bacteria in ankylosing spondylitis, rheumatoid arthritis, Crohn's disease and ulcerative colitis. Rheumatol Int. 1997;17:11-6.

577. Ganji L, Alebouyeh M, Shirazi MH, Eshraghi SS, Mirshafiey A, Daryani NE, et al. Dysbiosis of fecal microbiota and high frequency of Citrobacter, Klebsiella spp., and Actinomycetes in patients with irritable bowel syndrome and gastroenteritis. Gastroenterology and hepatology from bed to bench. 2016;9[4]:325.

578. Chen YR, Zheng HM, Zhang GX, Chen FL, Chen LD, Yang ZC. High Oscillospira abundance indicates constipation and low BMI in the Guangdong Gut Microbiome Project. Sci Rep. 2020;10[1]:9364.

579. Wang Z, Zhu H, Jiang Q, Zhu YZ. The gut microbiome as non-invasive biomarkers for identifying overweight people at risk for osteoarthritis. Microb Pathog. 2021;157:104976.

580. Cong J, Zhu H, Liu D, Li T, Zhang C, Zhu J, et al. A Pilot Study: Changes of Gut Microbiota in Post-surgery Colorectal Cancer Patients. Front Microbiol. 2018;9:2777.

581. Yildirim S, Nalbantoglu OU, Bayraktar A, Ercan FB, Gundogdu A, Velioglu HA, et al. Stratification of the Gut Microbiota Composition Landscape across the Alzheimer's Disease Continuum in a Turkish Cohort. mSystems. 2022;7[1]:e0000422.

582. Ye F, Gao X, Wang Z, Cao S, Liang G, He D, et al. Comparison of gut microbiota in autism spectrum disorders and neurotypical boys in China: A case-control study. Synth Syst Biotechnol. 2021;6[2]:120-6.

583. Wallen ZD, Demirkan A, Twa G, Cohen G, Dean MN, Standaert DG, et al. Metagenomics of Parkinson's disease implicates the gut microbiome in multiple disease mechanisms. Nat Commun. 2022;13[1]:6958.

584. Lin P, Ding B, Feng C, Yin S, Zhang T, Qi X, et al. Prevotella and Klebsiella proportions in fecal microbial communities are potential characteristic parameters for patients with major depressive disorder. J Affect Disord. 2017;207:300-4.

585. Halawa MR, El-Salam MA, Mostafa BM, Sallout SS. The Gut Microbiome, Lactobacillus acidophilus; Relation with Type 2 Diabetes Mellitus. Curr Diabetes Rev. 2019;15[6]:480-5.

586. Wang W, Chen L, Zhou R, Wang X, Song L, Huang S, et al. Increased proportions of Bifidobacterium and the Lactobacillus group and loss of butyrate-producing bacteria in inflammatory bowel disease. J Clin Microbiol. 2014;52[2]:398-406.

587. Szablewski L. Human Gut Microbiota in Health and Alzheimer's Disease. J Alzheimers Dis. 2018;62[2]:549-60.

588. De Angelis M, Francavilla R, Piccolo M, De Giacomo A, Gobbetti M. Autism spectrum disorders and intestinal microbiota. Gut Microbes. 2015;6[3]:207-13.

589. Macbeth WA, Kass EH, McDermott WV, Jr. Treatment of Hepatic Encephalopathy by Alteration of Intestinal Flora with Lactobacillus Acidophilus. Lancet. 1965;1[7382]:399-403.

590. Tellez Corral MA, Herrera Daza E, Cuervo Jimenez HK, Bravo Becerra MDM, Villamil JC, Hidalgo Martinez P, et al. Cryptic Oral Microbiota: What Is Its Role as Obstructive Sleep Apnea-Related Periodontal Pathogens? Int J Environ Res Public Health. 2023;20[3].

591. Sobhani I, Tap J, Roudot-Thoraval F, Roperch JP, Letulle S, Langella P, et al. Microbial dysbiosis in colorectal cancer [CRC] patients. PLoS One. 2011;6[1]:e16393.

592. Wasen C, Simonsen E, Ekwudo MN, Profant MR, Cox LM. The emerging role of the microbiome in Alzheimer's disease. Int Rev Neurobiol. 2022;167:101-39.

593. Lee Y, Park JY, Lee EH, Yang J, Jeong BR, Kim YK, et al. Rapid Assessment of Microbiota Changes in Individuals with Autism Spectrum Disorder Using Bacteria-derived Membrane Vesicles in Urine. Exp Neurobiol. 2017;26[5]:307-17.

594. Tetz G, Brown SM, Hao Y, Tetz V. Parkinson's disease and bacteriophages as its overlooked contributors. Sci Rep. 2018;8[1]:10812.

595. Ganci M, Suleyman E, Butt H, Ball M. Associations between self-reported psychological symptom severity and gut microbiota: further support for the microgenderome. BMC Psychiatry. 2022;22[1]:307.

596. Chen Z, Radjabzadeh D, Chen L, Kurilshikov A, Kavousi M, Ahmadizar F, et al. Association of Insulin Resistance and Type 2 Diabetes With Gut Microbial Diversity: A Microbiome-Wide Analysis From Population Studies. JAMA Netw Open. 2021;4[7]:e2118811.

597. Hu Y, Chen F, Ye H, Lu B. Integrative analysis of the gut microbiome and metabolome in a rat model with stress induced irritable bowel syndrome. Sci Rep. 2021;11[1]:17596.

598. Han DS, Wu WK, Liu PY, Yang YT, Hsu HC, Kuo CH, et al. Differences in the gut microbiome and reduced fecal butyrate in elders with low skeletal muscle mass. Clin Nutr. 2022;41[7]:1491-500.

599. Wang Y, Zhang Y, Qian Y, Xie YH, Jiang SS, Kang ZR, et al. Alterations in the oral and gut microbiome of colorectal cancer patients and association with host clinical factors. Int J Cancer. 2021.

600. Abraham D, Feher J, Scuderi GL, Szabo D, Dobolyi A, Cservenak M, et al. Exercise and probiotics attenuate the development of Alzheimer's disease in transgenic mice: Role of microbiome. Exp Gerontol. 2019;115:122-31.

601. Abujamel TS, Al-Otaibi NM, Abuaish S, AlHarbi RH, Assas MB, Alzahrani SA, et al. Different Alterations in Gut Microbiota between Bifidobacterium longum and Fecal Microbiota Transplantation Treatments in Propionic Acid Rat Model of Autism. Nutrients. 2022;14[3].

602. Chen TJ, Feng Y, Liu T, Wu TT, Chen YJ, Li X, et al. Fisetin Regulates Gut Microbiota and Exerts Neuroprotective Effect on Mouse Model of Parkinson's Disease. Front Neurosci. 2020;14:549037.

603. Tung TH, Tung YT, Lin IH, Shih CK, Nguyen NTK, Shabrina A, et al. Fish Oil, but Not Olive Oil, Ameliorates Depressive-Like Behavior and Gut Microbiota Dysbiosis in Rats under Chronic Mild Stress. Biomolecules. 2019;9[10].

604. Lucking EF, O'Connor KM, Strain CR, Fouhy F, Bastiaanssen TFS, Burns DP, et al. Chronic intermittent hypoxia disrupts cardiorespiratory homeostasis and gut microbiota composition in adult male guinea-pigs. EBioMedicine. 2018;38:191-205.

605. Torquati L, Gajanand T, Cox E, Willis C, Zaugg J, Keating S, et al. Effects of exercise intensity on gut microbiome composition and function in people with type 2 diabetes. European Journal of Sport Science. 2022:1-12.

606. Ghavami SB, Rostami E, Sephay AA, Shahrokh S, Balaii H, Aghdaei HA, et al. Alterations of the human gut Methanobrevibacter smithii as a biomarker for inflammatory bowel diseases. Microb Pathog. 2018;117:285-9.

607. Huang HL, Chen HT, Luo QL, Xu HM, He J, Li YQ, et al. Relief of irritable bowel syndrome by fecal microbiota transplantation is associated with changes in diversity and composition of the gut microbiota. J Dig Dis. 2019;20[8]:401-8.

608. Coker OO, Wu WKK, Wong SH, Sung JJY, Yu J. Altered Gut Archaea Composition and Interaction With Bacteria Are Associated With Colorectal Cancer. Gastroenterology. 2020;159[4]:1459-70 e5.

609. Garcez ML, Jacobs KR, Guillemin GJ. Microbiota Alterations in Alzheimer's Disease: Involvement of the Kynurenine Pathway and Inflammation. Neurotox Res. 2019;36[2]:424-36.

610. Newell C, Bomhof MR, Reimer RA, Hittel DS, Rho JM, Shearer J. Ketogenic diet modifies the gut microbiota in a murine model of autism spectrum disorder. Mol Autism. 2016;7[1]:37.

611. Ortega MA, Alvarez-Mon MA, Garcia-Montero C, Fraile-Martinez O, Guijarro LG, Lahera G, et al. Gut Microbiota Metabolites in Major Depressive Disorder-Deep Insights into Their Pathophysiological Role and Potential Translational Applications. Metabolites. 2022;12[1].

612. Zhang L, Zhang T, Sun J, Huang Y, Liu T, Ye Z, et al. Calorie restriction ameliorates hyperglycemia, modulates the disordered gut microbiota, and mitigates metabolic endotoxemia and inflammation in type 2 diabetic rats. J Endocrinol Invest. 2023;46[4]:699-711.

613. Pisani A, Rausch P, Bang C, Ellul S, Tabone T, Marantidis Cordina C, et al. Dysbiosis in the gut microbiota in patients with inflammatory bowel disease during remission. Microbiology Spectrum. 2022;10[3]:e00616-22.

614. Tap J, Derrien M, Törnblom H, Brazeilles R, Cools-Portier S, Doré J, et al. Identification of an intestinal microbiota signature associated with severity of irritable bowel syndrome. Gastroenterology. 2017;152[1]:111-23. e8.

615. Xu M, Mo X, Huang H, Chen X, Liu H, Peng Z, et al. Yeast beta-glucan alleviates cognitive deficit by regulating gut microbiota and metabolites in Abeta[1][-][42]-induced AD-like mice. Int J Biol Macromol. 2020;161:258-70.

616. Levi Mortera S, Vernocchi P, Basadonne I, Zandona A, Chierici M, Durighello M, et al. A metaproteomic-based gut microbiota profiling in children affected by autism spectrum disorders. J Proteomics. 2022;251:104407.

617. O'Donovan SM, Crowley EK, Brown JR, O'Sullivan O, O'Leary OF, Timmons S, et al. Nigral overexpression of alpha-synuclein in a rat Parkinson's disease model indicates alterations in the enteric nervous system and the gut microbiome. Neurogastroenterol Motil. 2020;32[1]:e13726.

618. Iebba V, Guerrieri F, Di Gregorio V, Levrero M, Gagliardi A, Santangelo F, et al. Combining amplicon sequencing and metabolomics in cirrhotic patients highlights distinctive microbiota features involved in bacterial translocation, systemic inflammation and hepatic encephalopathy. Sci Rep. 2018;8[1]:8210.

619. Tripathi A, Melnik AV, Xue J, Poulsen O, Meehan MJ, Humphrey G, et al. Intermittent Hypoxia and Hypercapnia, a Hallmark of Obstructive Sleep Apnea, Alters the Gut Microbiome and Metabolome. mSystems. 2018;3[3].

620. Shang J, Liu F, Zhang B, Dong K, Lu M, Jiang R, et al. Liraglutide-induced structural modulation of the gut microbiota in patients with type 2 diabetes mellitus. PeerJ. 2021;9:e11128.

621. Cremon C, Guglielmetti S, Gargari G, Taverniti V, Castellazzi AM, Valsecchi C, et al. Effect of Lactobacillus paracasei CNCM I-1572 on symptoms, gut microbiota, short chain fatty acids, and immune activation in patients with irritable bowel syndrome: A pilot randomized clinical trial. United European gastroenterology journal. 2018;6[4]:604-13.

622. Yang TW, Lee WH, Tu SJ, Huang WC, Chen HM, Sun TH, et al. Enterotype-based Analysis of Gut Microbiota along the Conventional Adenoma-Carcinoma Colorectal Cancer Pathway. Sci Rep. 2019;9[1]:10923.

623. Hang Z, Cai S, Lei T, Zhang X, Xiao Z, Wang D, et al. Transfer of Tumor-Bearing Mice Intestinal Flora Can Ameliorate Cognition in Alzheimer's Disease Mice. J Alzheimers Dis. 2022;86[3]:1287-300.

624. Zhai Q, Cen S, Jiang J, Zhao J, Zhang H, Chen W. Disturbance of trace element and gut microbiota profiles as indicators of autism spectrum disorder: A pilot study of Chinese children. Environ Res. 2019;171:501-9.

625. Zhang M, Li A, Yang Q, Li J, Wang L, Liu X, et al. Beneficial Effect of Alkaloids From Sophora alopecuroides L. on CUMS-Induced Depression Model Mice via Modulating Gut Microbiota. Front Cell Infect Microbiol. 2021;11:665159.

626. Wang Y, Ye X, Ding D, Lu Y. Characteristics of the intestinal flora in patients with peripheral neuropathy associated with type 2 diabetes. J Int Med Res. 2020;48[9]:300060520936806.

627. Zhou Y, Zhang F, Mao L, Feng T, Wang K, Xu M, et al. Bifico relieves irritable bowel syndrome by regulating gut microbiota dysbiosis and inflammatory cytokines. Eur J Nutr. 2023;62[1]:139-55.

628. Wu TR, Lin CS, Chang CJ, Lin TL, Martel J, Ko YF, et al. Gut commensal Parabacteroides goldsteinii plays a predominant role in the anti-obesity effects of polysaccharides isolated from Hirsutella sinensis. Gut. 2019;68[2]:248-62.

629. Sheng QS, He KX, Li JJ, Zhong ZF, Wang FX, Pan LL, et al. Comparison of Gut Microbiome in Human Colorectal Cancer in Paired Tumor and Adjacent Normal Tissues. Onco Targets Ther. 2020;13:635-46.

630. Sun ZZ, Li XY, Wang S, Shen L, Ji HF. Bidirectional interactions between curcumin and gut microbiota in transgenic mice with Alzheimer's disease. Appl Microbiol Biotechnol. 2020;104[8]:3507-15.

631. Gomez-Nguyen A, Basson AR, Dark-Fleury L, Hsu K, Osme A, Menghini P, et al. Parabacteroides distasonis induces depressive-like behavior in a mouse model of Crohn's disease. Brain Behav Immun. 2021;98:245-50.

632. Guarner F. The intestinal flora in inflammatory bowel disease: normal or abnormal? Curr Opin Gastroenterol. 2005;21[4]:414-8.

633. Shen H, Han J, Li Y, Lu C, Zhou J, Li Y, et al. Different host-specific responses in thyroid function and gut microbiota modulation between diet-induced obese and normal mice given the same dose of iodine. Appl Microbiol Biotechnol. 2019;103[8]:3537-47.

634. Salminen A. Activation of aryl hydrocarbon receptor [AhR] in Alzheimer's disease: role of tryptophan metabolites generated by gut host-microbiota. J Mol Med [Berl]. 2023;101[3]:201-22.

635. Dogra N, Mani RJ, Katare DP. The Gut-Brain Axis: Two Ways Signaling in Parkinson's Disease. Cell Mol Neurobiol. 2022;42[2]:315-32.

636. Ko CY, Fan JM, Hu AK, Su HZ, Yang JH, Huang LM, et al. Disruption of sleep architecture in Prevotella enterotype of patients with obstructive sleep apnea-hypopnea syndrome. Brain Behav. 2019;9[5]:e01287.

637. Leite AZ, Rodrigues NC, Gonzaga MI, Paiolo JCC, de Souza CA, Stefanutto NAV, et al. Detection of Increased Plasma Interleukin-6 Levels and Prevalence of Prevotella copri and Bacteroides vulgatus in the Feces of Type 2 Diabetes Patients. Front Immunol. 2017;8:1107.

638. Altomare A, Putignani L, Del Chierico F, Cocca S, Angeletti S, Ciccozzi M, et al. Gut mucosal-associated microbiota better discloses inflammatory bowel disease differential patterns than faecal microbiota. Dig Liver Dis. 2019;51[5]:648-56.

639. Su T, Liu R, Lee A, Long Y, Du L, Lai S, et al. Altered intestinal microbiota with increased abundance of Prevotella is associated with high risk of diarrhea-predominant irritable bowel syndrome. Gastroenterol Res Pract. 2018;2018.

640. Ericsson AC, Akter S, Hanson MM, Busi SB, Parker TW, Schehr RJ, et al. Differential susceptibility to colorectal cancer due to naturally occurring gut microbiota. Oncotarget. 2015;6[32]:33689-704.

641. Guo M, Peng J, Huang X, Xiao L, Huang F, Zuo Z. Gut Microbiome Features of Chinese Patients Newly Diagnosed with Alzheimer's Disease or Mild Cognitive Impairment. J Alzheimers Dis. 2021;80[1]:299-310.

642. Mertsalmi TH, Aho VTE, Pereira PAB, Paulin L, Pekkonen E, Auvinen P, et al. More than constipation - bowel symptoms in Parkinson's disease and their connection to gut microbiota. Eur J Neurol. 2017;24[11]:1375-83.

643. Dhiman RK. Gut microbiota, inflammation and hepatic encephalopathy: a puzzle with a solution in sight. J Clin Exp Hepatol. 2012;2[3]:207-10.

644. Kobyliak N, Falalyeyeva T, Mykhalchyshyn G, Kyriienko D, Komissarenko I. Effect of alive probiotic on insulin resistance in type 2 diabetes patients: Randomized clinical trial. Diabetes Metab Syndr. 2018;12[5]:617-24.

645. Brahe LK, Astrup A, Larsen LH. Can We Prevent Obesity-Related Metabolic Diseases by Dietary Modulation of the Gut Microbiota? Adv Nutr. 2016;7[1]:90-101.

646. Lacroix S, Pechereau F, Leblanc N, Boubertakh B, Houde A, Martin C, et al. Rapid and Concomitant Gut Microbiota and Endocannabinoidome Response to Diet-Induced Obesity in Mice. mSystems. 2019;4[6].

647. Wagner J, Short K, Catto-Smith AG, Cameron DJ, Bishop RF, Kirkwood CD. Identification and characterisation of Pseudomonas 16S ribosomal DNA from ileal biopsies of children with Crohn's disease. PLoS One. 2008;3[10]:e3578.

648. Kerckhoffs AP, Ben-Amor K, Samsom M, van der Rest ME, de Vogel J, Knol J, et al. Molecular analysis of faecal and duodenal samples reveals significantly higher prevalence and numbers of Pseudomonas aeruginosa in irritable bowel syndrome. J Med Microbiol. 2011;60[2]:236-45.

649. Petriz BA, Castro AP, Almeida JA, Gomes CP, Fernandes GR, Kruger RH, et al. Exercise induction of gut microbiota modifications in obese, non-obese and hypertensive rats. BMC Genomics. 2014;15:1-13.

650. Gao Z, Guo B, Gao R, Zhu Q, Qin H. Microbiota disbiosis is associated with colorectal cancer. Front Microbiol. 2015;6:20.

651. Wu S, Liu X, Jiang R, Yan X, Ling Z. Roles and Mechanisms of Gut Microbiota in Patients With Alzheimer's Disease. Front Aging Neurosci. 2021;13:650047.

652. Pulikkan J, Mazumder A, Grace T. Role of the Gut Microbiome in Autism Spectrum Disorders. Adv Exp Med Biol. 2019;1118:253-69.

653. Wang Y, Tong Q, Ma SR, Zhao ZX, Pan LB, Cong L, et al. Oral berberine improves brain dopa/dopamine levels to ameliorate Parkinson's disease by regulating gut microbiota. Signal Transduct Target Ther. 2021;6[1]:77.

654. Waclawikova B, El Aidy S. Role of Microbiota and Tryptophan Metabolites in the Remote Effect of Intestinal Inflammation on Brain and Depression. Pharmaceuticals [Basel]. 2018;11[3].

655. Jang S, Kim Y, Lee C, Kwon B, Noh J, Jee JJ, et al. The effect of formula-based nutritional treatment on colitis in a murine model. J Korean Med Sci. 2021;36[50].

656. Seo KH, Kim DH, Yokoyama WH, Kim H. Synbiotic Effect of Whole Grape Seed Flour and Newly Isolated Kefir Lactic Acid Bacteria on Intestinal Microbiota of Diet-Induced Obese Mice. J Agric Food Chem. 2020;68[46]:13131-7.

657. Romero-Garmendia I, Garcia-Etxebarria K. Host Genetics and Microbiota Interactions in Colorectal Cancer: Shared or Independent Risk? Microorganisms. 2022;10[11].

658. Kim JE, Roh YJ, Choi YJ, Lee SJ, Jin YJ, Song HJ, et al. Dysbiosis of Fecal Microbiota in Tg2576 Mice for Alzheimer's Disease during Pathological Constipation. Int J Mol Sci. 2022;23[23].

659. Getachew B, Tizabi Y. Effects of C-Terminal Domain of the Heavy Chain of Tetanus Toxin on Gut Microbiota in a Rat Model of Depression. Clin Pharmacol Transl Med. 2019;3[2]:152-9.

660. Faden H. The Role of Faecalibacterium, Roseburia, and Butyrate in Inflammatory Bowel Disease. Dig Dis. 2022;40[6]:793-5.

661. Ruusunen A, Rocks T, Jacka F, Loughman A. The gut microbiome in anorexia nervosa: relevance for nutritional rehabilitation. Psychopharmacology [Berl]. 2019;236[5]:1545-58.

662. Robinson H, Barrett H, Gomez-Arango L, McIntyre HD, Callaway L, Dekker Nitert M. Ketonuria Is Associated with Changes to the Abundance of Roseburia in the Gut Microbiota of Overweight and Obese Women at 16 Weeks Gestation: A Cross-Sectional Observational Study. Nutrients. 2019;11[8].

663. Geng J, Fan H, Tang X, Zhai H, Zhang Z. Diversified pattern of the human colorectal cancer microbiome. Gut Pathog. 2013;5[1]:2.

664. Jiang L, Li JC, Tang BS, Guo JF. Associations Between Gut Microbiota and Parkinson's Disease: A Bidirectional Mendelian Randomization Analysis. Eur J Neurol. 2023.

665. Qu W, Liu S, Zhang W, Zhu H, Tao Q, Wang H, et al. Impact of traditional Chinese medicine treatment on chronic unpredictable mild stress-induced depression-like behaviors: intestinal microbiota and gut microbiome function. Food Funct. 2019;10[9]:5886-97.

666. Henke MT, Kenny DJ, Cassilly CD, Vlamakis H, Xavier RJ, Clardy J. Ruminococcus gnavus, a member of the human gut microbiome associated with Crohn’s disease, produces an inflammatory polysaccharide. Proceedings of the National Academy of Sciences. 2019;116[26]:12672-7.

667. Salas-Perez F, Assmann TS, Ramos-Lopez O, Martinez JA, Riezu-Boj JI, Milagro FI. Crosstalk between Gut Microbiota and Epigenetic Markers in Obesity Development: Relationship between Ruminococcus, BMI, and MACROD2/SEL1L2 Methylation. Nutrients. 2023;15[7].

668. Wang L, Christophersen CT, Sorich MJ, Gerber JP, Angley MT, Conlon MA. Increased abundance of Sutterella spp. and Ruminococcus torques in feces of children with autism spectrum disorder. Mol Autism. 2013;4[1]:42.

669. Lukic I, Getselter D, Ziv O, Oron O, Reuveni E, Koren O, et al. Antidepressants affect gut microbiota and Ruminococcus flavefaciens is able to abolish their effects on depressive-like behavior. Transl Psychiatry. 2019;9[1]:133.

670. Silamikele L, Saksis R, Silamikelis I, Kotovica PP, Briviba M, Kalnina I, et al. Spatial variation of the gut microbiome in response to long-term metformin treatment in high-fat diet-induced type 2 diabetes mouse model of both sexes. Gut Microbes. 2023;15[1]:2188663.

671. Ma H-Q, Yu T-T, Zhao X-J, Zhang Y, Zhang H-J. Fecal microbial dysbiosis in Chinese patients with inflammatory bowel disease. World J Gastroenterol. 2018;24[13]:1464.

672. Mohamad NE, Yeap SK, Ky H, Liew NWC, Beh BK, Boo SY, et al. Pineapple Vinegar Regulates Obesity-Related Genes and Alters the Gut Microbiota in High-Fat Diet [HFD] C57BL/6 Obese Mice. Evid Based Complement Alternat Med. 2020;2020:1257962.

673. Getachew B, Aubee JI, Schottenfeld RS, Csoka AB, Thompson KM, Tizabi Y. Ketamine interactions with gut-microbiota in rats: relevance to its antidepressant and anti-inflammatory properties. BMC Microbiol. 2018;18[1]:222.

674. Zhao X, Zhang Y, Guo R, Yu W, Zhang F, Wu F, et al. The Alteration in Composition and Function of Gut Microbiome in Patients with Type 2 Diabetes. J Diabetes Res. 2020;2020:8842651.

675. Cheung YB, Xu Y, Mangani C, Fan YM, Dewey KG, Salminen SJ, et al. Gut microbiota in Malawian infants in a nutritional supplementation trial. Trop Med Int Health. 2016;21[2]:283-90.

676. Nie Q, Chen H, Hu J, Fan S, Nie S. Dietary compounds and traditional Chinese medicine ameliorate type 2 diabetes by modulating gut microbiota. Crit Rev Food Sci Nutr. 2019;59[6]:848-63.

677. Correa TAF, Rogero MM, Hassimotto NMA, Lajolo FM. The Two-Way Polyphenols-Microbiota Interactions and Their Effects on Obesity and Related Metabolic Diseases. Front Nutr. 2019;6:188.

678. Kasai C, Sugimoto K, Moritani I, Tanaka J, Oya Y, Inoue H, et al. Comparison of human gut microbiota in control subjects and patients with colorectal carcinoma in adenoma: Terminal restriction fragment length polymorphism and next-generation sequencing analyses. Oncol Rep. 2016;35[1]:325-33.

679. Chen HM, Kuo PH, Hsu CY, Chiu YH, Liu YW, Lu ML, et al. Psychophysiological Effects of Lactobacillus plantarum PS128 in Patients with Major Depressive Disorder: A Preliminary 8-Week Open Trial. Nutrients. 2021;13[11].

680. Moon J-Y, Zolnik CP, Wang Z, Qiu Y, Usyk M, Wang T, et al. Gut microbiota and plasma metabolites associated with diabetes in women with, or at high risk for, HIV infection. EBioMedicine. 2018;37:392-400.

681. Zareef R, Younis N, Mahfouz R. Inflammatory bowel disease: a key role for microbiota? Meta Gene. 2020;25:100713.

682. Chen X, Zhang D, Sun H, Jiang F, Shen Y, Wei P, et al. Characterization of the gut microbiota in Chinese children with overweight and obesity using 16S rRNA gene sequencing. PeerJ. 2021;9:e11439.

683. Liu C, Shao W, Gao M, Liu J, Guo Q, Jin J, et al. Changes in intestinal flora in patients with type 2 diabetes on a low-fat diet during 6 months of follow-up. Exp Ther Med. 2020;20[5]:40.

684. Zhong Y-B, Kang Z-P, Wang M-X, Long J, Wang H-Y, Huang J-Q, et al. Curcumin ameliorated dextran sulfate sodium-induced colitis via regulating the homeostasis of DCs and Treg and improving the composition of the gut microbiota. J Funct Foods. 2021;86:104716.

685. Lin C, Li B, Tu C, Chen X, Guo M. Correlations between Intestinal Microbiota and Clinical Characteristics in Colorectal Adenoma/Carcinoma. Biomed Res Int. 2022;2022:3140070.

686. Wu Y, Niu X, Li P, Tong T, Wang Q, Zhang M, et al. Lactobacillaceae improve cognitive dysfunction via regulating gut microbiota and suppressing Abeta deposits and neuroinflammation in APP/PS1 mice. Arch Microbiol. 2023;205[4]:118.

687. Singh Y, El-Hadidi M, Admard J, Wassouf Z, Schulze-Hentrich JM, Kohlhofer U, et al. Enriched Environmental Conditions Modify the Gut Microbiome Composition and Fecal Markers of Inflammation in Parkinson's Disease. Front Neurosci. 2019;13:1032.

688. He K, Hu Y, Ma H, Zou Z, Xiao Y, Yang Y, et al. Rhizoma Coptidis alkaloids alleviate hyperlipidemia in B6 mice by modulating gut microbiota and bile acid pathways. Biochim Biophys Acta. 2016;1862[9]:1696-709.

689. Katsidzira L, Ocvirk S, Wilson A, Li J, Mahachi CB, Soni D, et al. Differences in Fecal Gut Microbiota, Short-Chain Fatty Acids and Bile Acids Link Colorectal Cancer Risk to Dietary Changes Associated with Urbanization Among Zimbabweans. Nutr Cancer. 2019;71[8]:1313-24.

690. Yao Y, Qi X, Jia Y, Ye J, Chu X, Wen Y, et al. Evaluating the interactive effects of dietary habits and human gut microbiome on the risks of depression and anxiety. Psychol Med. 2022:1-9.

691. Bui TI, Gill AL, Mooney RA, Gill SR. Modulation of Gut Microbiota Metabolism in Obesity-Related Type 2 Diabetes Reduces Osteomyelitis Severity. Microbiol Spectr. 2022;10[2]:e0017022.

692. Azimirad M, Krutova M, Balaii H, Kodori M, Shahrokh S, Azizi O, et al. Coexistence of Clostridioides difficile and Staphylococcus aureus in gut of Iranian outpatients with underlying inflammatory bowel disease. Anaerobe. 2020;61:102113.

693. Cani PD, Delzenne NM. Interplay between obesity and associated metabolic disorders: new insights into the gut microbiota. Curr Opin Pharmacol. 2009;9[6]:737-43.

694. Pistollato F, Sumalla Cano S, Elio I, Masias Vergara M, Giampieri F, Battino M. Role of gut microbiota and nutrients in amyloid formation and pathogenesis of Alzheimer disease. Nutr Rev. 2016;74[10]:624-34.

695. Chen Y, Xu J, Chen Y. Regulation of Neurotransmitters by the Gut Microbiota and Effects on Cognition in Neurological Disorders. Nutrients. 2021;13[6].

696. Wilkowska A, Szalach LP, Cubala WJ. Gut Microbiota in Depression: A Focus on Ketamine. Front Behav Neurosci. 2021;15:693362.

697. Sun X, Chi X, Zhao Y, Liu S, Xing H. Characteristics and Clinical Significance of Intestinal Microbiota in Patients with Chronic Hepatitis B Cirrhosis and Type 2 Diabetes Mellitus. J Diabetes Res. 2022;2022:1826181.

698. Heidarian F, Noormohammadi Z, Aghdaei HA, Alebouyeh M. Relative abundance of streptococcus spp. and its association with disease activity in inflammatory bowel disease patients compared with controls. Archives of Clinical Infectious Diseases. 2017;12[2].

699. Hong SN, Rhee P-L. Unraveling the ties between irritable bowel syndrome and intestinal microbiota. World journal of gastroenterology: WJG. 2014;20[10]:2470.

700. Wang M, Doenyas C, Wan J, Zeng S, Cai C, Zhou J, et al. Virulence factor-related gut microbiota genes and immunoglobulin A levels as novel markers for machine learning-based classification of autism spectrum disorder. Comput Struct Biotechnol J. 2021;19:545-54.

701. Li Z, Lu G, Luo E, Wu B, Li Z, Guo J, et al. Oral, Nasal, and Gut Microbiota in Parkinson's Disease. Neuroscience. 2022;480:65-78.

702. Smith RP, Easson C, Lyle SM, Kapoor R, Donnelly CP, Davidson EJ, et al. Gut microbiome diversity is associated with sleep physiology in humans. PLoS One. 2019;14[10]:e0222394.

703. Bai Z, Huang X, Wu G, Ye H, Huang W, Nie Q, et al. Polysaccharides from red kidney bean alleviating hyperglycemia and hyperlipidemia in type 2 diabetic rats via gut microbiota and lipid metabolic modulation. Food Chem. 2023;404[Pt A]:134598.

704. Loh G, Blaut M. Role of commensal gut bacteria in inflammatory bowel diseases. Gut microbes. 2012;3[6]:544-55.

705. Khoo XH, Chong CW, Talha AM, Philip K, Teh CSJ, Isa AM, et al. The impact of diet and ethnicity on Gut Microbiota Variation in Irritable Bowel Syndrome: A Multi-centre Study. J Gastroenterol Hepatol. 2023.

706. Chavez-Carbajal A, Nirmalkar K, Perez-Lizaur A, Hernandez-Quiroz F, Ramirez-Del-Alto S, Garcia-Mena J, et al. Gut Microbiota and Predicted Metabolic Pathways in a Sample of Mexican Women Affected by Obesity and Obesity Plus Metabolic Syndrome. Int J Mol Sci. 2019;20[2].

707. Natalello G, Bosello SL, Paroni Sterbini F, Posteraro B, De Lorenzis E, Canestrari GB, et al. Gut microbiota analysis in systemic sclerosis according to disease characteristics and nutritional status. Clin Exp Rheumatol. 2020;38 Suppl 125[3]:73-84.

708. Zhang M, Lv Y, Hou S, Liu Y, Wang Y, Wan X. Differential Mucosal Microbiome Profiles across Stages of Human Colorectal Cancer. Life [Basel]. 2021;11[8].

709. Wang Y, Shen R, Liu M, Zhou Q, Zhai Y-h, Fan L-h, et al. Metagenomic analysis of Tongxie Yaofang therapy for rat models of ulcerative colitis with liver depression and spleen deficiency syndrome. All Life. 2023;16[1]:1-12.

710. Bischoff SC, Nguyen NK, Seethaler B, Beisner J, Kügler P, Stefan T. Gut Microbiota Patterns Predicting Long-Term Weight Loss Success in Individuals with Obesity Undergoing Nonsurgical Therapy. Nutrients. 2022;14[15]:3182.

711. Yang W, Shao L, Heizhati M, Wu T, Yao X, Wang Y, et al. Oropharyngeal Microbiome in Obstructive Sleep Apnea: Decreased Diversity and Abundance. J Clin Sleep Med. 2019;15[12]:1777-88.

712. Tai N, Wong FS, Wen L. The role of gut microbiota in the development of type 1, type 2 diabetes mellitus and obesity. Rev Endocr Metab Disord. 2015;16[1]:55-65.

713. Heimesaat MM, Fischer A, Siegmund B, Kupz A, Niebergall J, Fuchs D, et al. Shift towards pro-inflammatory intestinal bacteria aggravates acute murine colitis via Toll-like receptors 2 and 4. PLoS One. 2007;2[7]:e662.

714. Verdam FJ, Fuentes S, de Jonge C, Zoetendal EG, Erbil R, Greve JW, et al. Human intestinal microbiota composition is associated with local and systemic inflammation in obesity. Obesity [Silver Spring]. 2013;21[12]:E607-15.

715. Purcell RV, Visnovska M, Biggs PJ, Schmeier S, Frizelle FA. Distinct gut microbiome patterns associate with consensus molecular subtypes of colorectal cancer. Sci Rep. 2017;7[1]:11590.

716. Wang J, Ye F, Cheng X, Zhang X, Liu F, Liu G, et al. The Effects of LW-AFC on Intestinal Microbiome in Senescence-Accelerated Mouse Prone 8 Strain, a Mouse Model of Alzheimer's Disease. J Alzheimers Dis. 2016;53[3]:907-19.

717. Afroz KF, Reyes N, Young K, Parikh K, Misra V, Alvina K. Author Correction: Altered gut microbiome and autism like behavior are associated with parental high salt diet in male mice. Sci Rep. 2022;12[1]:5686.

718. Zhu Y, Huan F, Wang J, Xie X, Yu G, Wang X, et al. 1-Methyl-4-phenyl-1,2,3,6-tetrahydropyridine Induced Parkinson's Disease in Mouse: Potential Association between Neurotransmitter Disturbance and Gut Microbiota Dysbiosis. ACS Chem Neurosci. 2020;11[20]:3366-76.

719. Lai WT, Zhao J, Xu SX, Deng WF, Xu D, Wang MB, et al. Shotgun metagenomics reveals both taxonomic and tryptophan pathway differences of gut microbiota in bipolar disorder with current major depressive episode patients. J Affect Disord. 2021;278:311-9.

720. Cai Y, Juszczak HM, Cope EK, Goldberg AN. The microbiome in obstructive sleep apnea. Sleep. 2021;44[8].

721. Zhao F, Liu Q, Cao J, Xu Y, Pei Z, Fan H, et al. A sea cucumber [Holothuria leucospilota] polysaccharide improves the gut microbiome to alleviate the symptoms of type 2 diabetes mellitus in Goto-Kakizaki rats. Food Chem Toxicol. 2020;135:110886.

722. Li H, Christman LM, Li R, Gu L. Synergic interactions between polyphenols and gut microbiota in mitigating inflammatory bowel diseases. Food Funct. 2020;11[6]:4878-91.

723. Angelakis E, Bachar D, Yasir M, Musso D, Djossou F, Melenotte C, et al. Comparison of the gut microbiota of obese individuals from different geographic origins. New Microbes New Infect. 2019;27:40-7.

724. Fox M, Knorr DA, Haptonstall KM. Alzheimer's disease and symbiotic microbiota: an evolutionary medicine perspective. Ann N Y Acad Sci. 2019;1449[1]:3-24.

725. Scassellati C, Marizzoni M, Cattane N, Lopizzo N, Mombelli E, Riva MA, et al. The Complex Molecular Picture of Gut and Oral Microbiota-Brain-Depression System: What We Know and What We Need to Know. Front Psychiatry. 2021;12:722335.

726. Bajer L, Kverka M, Kostovcik M, Macinga P, Dvorak J, Stehlikova Z, et al. Distinct gut microbiota profiles in patients with primary sclerosing cholangitis and ulcerative colitis. World J Gastroenterol. 2017;23[25]:4548.

727. de Souza AZ, Zambom AZ, Abboud KY, Reis SK, Tannihao F, Guadagnini D, et al. Oral supplementation with L-glutamine alters gut microbiota of obese and overweight adults: A pilot study. Nutrition. 2015;31[6]:884-9.

728. Shi F, Liu G, Lin Y, Guo CL, Han J, Chu ESH, et al. Altered gut microbiome composition by appendectomy contributes to colorectal cancer. Oncogene. 2023;42[7]:530-40.

729. Zhang M, Ma W, Zhang J, He Y, Wang J. Analysis of gut microbiota profiles and microbe-disease associations in children with autism spectrum disorders in China. Sci Rep. 2018;8[1]:13981.

730. Hu S, Li A, Huang T, Lai J, Li J, Sublette ME, et al. Gut Microbiota Changes in Patients with Bipolar Depression. Adv Sci [Weinh]. 2019;6[14]:1900752.

731. Salamone D, Rivellese AA, Vetrani C. The relationship between gut microbiota, short-chain fatty acids and type 2 diabetes mellitus: the possible role of dietary fibre. Acta Diabetol. 2021;58[9]:1131-8.

732. Zuo T, Kamm MA, Colombel J-F, Ng SC. Urbanization and the gut microbiota in health and inflammatory bowel disease. Nature Reviews Gastroenterology & Hepatology. 2018;15[7]:440-52.

733. Chen J, He X, Huang J. Diet effects in gut microbiome and obesity. J Food Sci. 2014;79[4]:R442-51.

734. Zhang W, Zhang J, Liu T, Xing J, Zhang H, Wang D, et al. Bidirectional effects of intestinal microbiota and antibiotics: a new strategy for colorectal cancer treatment and prevention. J Cancer Res Clin Oncol. 2022;148[9]:2387-404.

735. Allaband C, Lingaraju A, Martino C, Russell B, Tripathi A, Poulsen O, et al. Intermittent hypoxia and hypercapnia alter diurnal rhythms of luminal gut microbiome and metabolome. Msystems. 2021;6[3]:e00116-21.

736. Remely M, Hippe B, Zanner J, Aumueller E, Brath H, Haslberger AG. Gut Microbiota of Obese, Type 2 Diabetic Individuals is Enriched in Faecalibacterium prausnitzii, Akkermansia muciniphila and Peptostreptococcus anaerobius after Weight Loss. Endocr Metab Immune Disord Drug Targets. 2016;16[2]:99-106.

737. Zhang T, Ji X, Lu G, Zhang F. The potential of Akkermansia muciniphila in inflammatory bowel disease. Appl Microbiol Biotechnol. 2021;105[14-15]:5785-94.

738. Depommier C, Everard A, Druart C, Plovier H, Van Hul M, Vieira-Silva S, et al. Supplementation with Akkermansia muciniphila in overweight and obese human volunteers: a proof-of-concept exploratory study. Nat Med. 2019;25[7]:1096-103.

739. Osman MA, Neoh HM, Ab Mutalib NS, Chin SF, Mazlan L, Raja Ali RA, et al. Parvimonas micra, Peptostreptococcus stomatis, Fusobacterium nucleatum and Akkermansia muciniphila as a four-bacteria biomarker panel of colorectal cancer. Sci Rep. 2021;11[1]:2925.

740. Ou Z, Deng L, Lu Z, Wu F, Liu W, Huang D, et al. Protective effects of Akkermansia muciniphila on cognitive deficits and amyloid pathology in a mouse model of Alzheimer's disease. Nutr Diabetes. 2020;10[1]:12.

741. Fang X, Li FJ, Hong DJ. Potential Role of Akkermansia muciniphila in Parkinson's Disease and Other Neurological/Autoimmune Diseases. Curr Med Sci. 2021;41[6]:1172-7.

742. Ding Y, Bu F, Chen T, Shi G, Yuan X, Feng Z, et al. A next-generation probiotic: Akkermansia muciniphila ameliorates chronic stress-induced depressive-like behavior in mice by regulating gut microbiota and metabolites. Appl Microbiol Biotechnol. 2021;105[21-22]:8411-26.

743. Xu J, Lian F, Zhao L, Zhao Y, Chen X, Zhang X, et al. Structural modulation of gut microbiota during alleviation of type 2 diabetes with a Chinese herbal formula. ISME J. 2015;9[3]:552-62.

744. Dziarski R, Park SY, Kashyap DR, Dowd SE, Gupta D. Pglyrp-Regulated Gut Microflora Prevotella falsenii, Parabacteroides distasonis and Bacteroides eggerthii Enhance and Alistipes finegoldii Attenuates Colitis in Mice. PLoS One. 2016;11[1]:e0146162.

745. Lotankar M, Mokkala K, Houttu N, Koivuniemi E, Sorensen N, Nielsen HB, et al. Distinct Diet-Microbiota-Metabolism Interactions in Overweight and Obese Pregnant Women: a Metagenomics Approach. Microbiol Spectr. 2022;10[2]:e0089321.

746. Sun T, Liu S, Zhou Y, Yao Z, Zhang D, Cao S, et al. Evolutionary biologic changes of gut microbiota in an 'adenoma-carcinoma sequence' mouse colorectal cancer model induced by 1, 2-Dimethylhydrazine. Oncotarget. 2017;8[1]:444-57.

747. Li Z, Zhu H, Guo Y, Du X, Qin C. Gut microbiota regulate cognitive deficits and amyloid deposition in a model of Alzheimer's disease. J Neurochem. 2020;155[4]:448-61.

748. Averina O, Kovtun A, Danilenko V. Bacterial Genes of Metabolite Biomarkers of Autism Spectrum Disorders in Gut Microbiota of Young Children: Detection by Real-Time PCR and In Silico Analysis. Russian Journal of Genetics. 2020;56:1260-8.

749. Avagliano C, Coretti L, Lama A, Pirozzi C, De Caro C, De Biase D, et al. Dual-Hit Model of Parkinson's Disease: Impact of Dysbiosis on 6-Hydroxydopamine-Insulted Mice-Neuroprotective and Anti-Inflammatory Effects of Butyrate. Int J Mol Sci. 2022;23[12].

750. Liu P, Gao M, Liu Z, Zhang Y, Tu H, Lei L, et al. Gut Microbiome Composition Linked to Inflammatory Factors and Cognitive Functions in First-Episode, Drug-Naive Major Depressive Disorder Patients. Front Neurosci. 2021;15:800764.

751. Han Y, Xu J, Yan Y, Zhao X. Dynamics of the gut microbiota in rats after hypobaric hypoxia exposure. PeerJ. 2022;10:e14090.

752. Kulkarni P, Devkumar P, Chattopadhyay I. Could dysbiosis of inflammatory and anti-inflammatory gut bacteria have an implications in the development of type 2 diabetes? A pilot investigation. BMC Res Notes. 2021;14[1]:1-7.

753. Hou Y, Dong L, Lu X, Shi H, Xu B, Zhong W, et al. Distinctions Between Fecal and Intestinal Mucosal Microbiota in Subgroups of Irritable Bowel Syndrome. Dig Dis Sci. 2022;67[12]:5580-92.

754. Del Chierico F, Abbatini F, Russo A, Quagliariello A, Reddel S, Capoccia D, et al. Gut Microbiota Markers in Obese Adolescent and Adult Patients: Age-Dependent Differential Patterns. Front Microbiol. 2018;9:1210.

755. Song CH, Kim N, Nam RH, Choi SI, Jang JY, Lee HN. Changes in Gut Microbiome upon Orchiectomy and Testosterone Administration in AOM/DSS-Induced Colon Cancer Mouse Model. Cancer Res Treat. 2023;55[1]:196-218.

756. Wagner VE, Dey N, Guruge J, Hsiao A, Ahern PP, Semenkovich NP, et al. Effects of a gut pathobiont in a gnotobiotic mouse model of childhood undernutrition. Sci Transl Med. 2016;8[366]:366ra164.

757. Shen WD, Lin X, Liu HM, Li BY, Qiu X, Lv WQ, et al. Gut microbiota accelerates obesity in peri-/post-menopausal women via Bacteroides fragilis and acetic acid. Int J Obes [Lond]. 2022;46[10]:1918-24.

758. Wei Z, Cao S, Liu S, Yao Z, Sun T, Li Y, et al. Could gut microbiota serve as prognostic biomarker associated with colorectal cancer patients' survival? A pilot study on relevant mechanism. Oncotarget. 2016;7[29]:46158-72.

759. Zou R, Xu F, Wang Y, Duan M, Guo M, Zhang Q, et al. Changes in the Gut Microbiota of Children with Autism Spectrum Disorder. Autism Res. 2020;13[9]:1614-25.

760. Luna RA, Foster JA. Gut brain axis: diet microbiota interactions and implications for modulation of anxiety and depression. Curr Opin Biotechnol. 2015;32:35-41.

761. Schafer DF, Jones EA. Hepatic encephalopathy and the gamma-aminobutyric-acid neurotransmitter system. Lancet. 1982;1[8262]:18-20.

762. Ren Y, Hao L, Liu J, Wang P, Ding Q, Chen C, et al. Alterations in the Gut Microbiota in Pregnant Women with Pregestational Type 2 Diabetes Mellitus. mSystems. 2023;8[2]:e0114622.

763. Hu M, Caldarelli G, Gili T. Network analysis of a complex disease: the gut microbiota in the inflammatory bowel disease case. arXiv preprint arXiv:220807763. 2022.

764. Di Luccia B, Ahern PP, Griffin NW, Cheng J, Guruge JL, Byrne AE, et al. Combined Prebiotic and Microbial Intervention Improves Oral Cholera Vaccination Responses in a Mouse Model of Childhood Undernutrition. Cell Host Microbe. 2020;27[6]:899-908 e5.

765. West KA, Yin X, Rutherford EM, Wee B, Choi J, Chrisman BS, et al. Multi-angle meta-analysis of the gut microbiome in Autism Spectrum Disorder: a step toward understanding patient subgroups. Sci Rep. 2022;12[1]:17034.

766. Bi M, Feng L, He J, Liu C, Wang Y, Jiang H, et al. Emerging insights between gut microbiome dysbiosis and Parkinson’s disease: Pathogenic and clinical relevance. Ageing Research Reviews. 2022:101759.

767. Wenxin L, Du Q, Dingding H, Zhang H. The Gut Microbiome and Obstructive Sleep Apnea Syndrome in Children. Sleep Med. 2022.

768. Wu X, Ma C, Han L, Nawaz M, Gao F, Zhang X, et al. Molecular characterisation of the faecal microbiota in patients with type II diabetes. Curr Microbiol. 2010;61[1]:69-78.

769. Schirmer M, Franzosa EA, Lloyd-Price J, McIver LJ, Schwager R, Poon TW, et al. Dynamics of metatranscription in the inflammatory bowel disease gut microbiome. Nat Microbiol. 2018;3[3]:337-46.

770. Hata T, Miyata N, Takakura S, Yoshihara K, Asano Y, Kimura-Todani T, et al. The Gut Microbiome Derived From Anorexia Nervosa Patients Impairs Weight Gain and Behavioral Performance in Female Mice. Endocrinology. 2019;160[10]:2441-52.

771. Nicolucci AC, Hume MP, Martinez I, Mayengbam S, Walter J, Reimer RA. Prebiotics Reduce Body Fat and Alter Intestinal Microbiota in Children Who Are Overweight or With Obesity. Gastroenterology. 2017;153[3]:711-22.

772. Liang S, Sin ZY, Yu J, Zhao S, Xi Z, Bruzzone R, et al. Multi-cohort analysis of depression-associated gut bacteria sheds insight on bacterial biomarkers across populations. Cell Mol Life Sci. 2022;80[1]:9.

773. Qian X, Si Q, Lin G, Zhu M, Lu J, Zhang H, et al. Bifidobacterium adolescentis Is Effective in Relieving Type 2 Diabetes and May Be Related to Its Dominant Core Genome and Gut Microbiota Modulation Capacity. Nutrients. 2022;14[12]:2479.

774. Kowalska-Duplaga K, Gosiewski T, Kapusta P, Sroka-Oleksiak A, Wędrychowicz A, Pieczarkowski S, et al. Differences in the intestinal microbiome of healthy children and patients with newly diagnosed Crohn’s disease. Sci Rep. 2019;9[1]:1-11.

775. Iribarren C, Magnusson MK, Vigsnaes LK, Aziz I, Amundsen ID, Suligoj T, et al. The Effects of Human Milk Oligosaccharides on Gut Microbiota, Metabolite Profiles and Host Mucosal Response in Patients with Irritable Bowel Syndrome. Nutrients. 2021;13[11].

776. Azcarate-Peril MA, Sikes M, Bruno-Barcena JM. The intestinal microbiota, gastrointestinal environment and colorectal cancer: a putative role for probiotics in prevention of colorectal cancer? Am J Physiol Gastrointest Liver Physiol. 2011;301[3]:G401-24.

777. Mulle JG, Sharp WG, Cubells JF. The gut microbiome: a new frontier in autism research. Curr Psychiatry Rep. 2013;15[2]:337.

778. Alfonsetti M, Castelli V, d'Angelo M. Are We What We Eat? Impact of Diet on the Gut–Brain Axis in Parkinson's Disease. Nutrients. 2022;14[2]:380.

779. Guo Y, Xie JP, Deng K, Li X, Yuan Y, Xuan Q, et al. Prophylactic Effects of Bifidobacterium adolescentis on Anxiety and Depression-Like Phenotypes After Chronic Stress: A Role of the Gut Microbiota-Inflammation Axis. Front Behav Neurosci. 2019;13:126.

780. Bloom PP, Donlan J, Torres Soto M, Daidone M, Hohmann E, Chung RT. Fecal microbiota transplant improves cognition in hepatic encephalopathy and its effect varies by donor and recipient. Hepatol Commun. 2022;6[8]:2079-89.

781. Suceveanu AI, Stoian AP, Parepa I, Voinea C, Hainarosie R, Manuc D, et al. Gut microbiota patterns in obese and type 2 diabetes [T2D] patients from romanian black sea coast region. Rev Chim. 2018;69[8]:2260-7.

782. Lee KJ, Tack J. Altered intestinal microbiota in irritable bowel syndrome. Neurogastroenterol Motil. 2010;22[5]:493-8.

783. Wang Q, Wang K, Wu W, Lv L, Bian X, Yang L, et al. Administration of Bifidobacterium bifidum CGMCC 15068 modulates gut microbiota and metabolome in azoxymethane [AOM]/dextran sulphate sodium [DSS]-induced colitis-associated colon cancer [CAC] in mice. Appl Microbiol Biotechnol. 2020;104[13]:5915-28.

784. Kim H, Kim S, Park SJ, Park G, Shin H, Park MS, et al. Administration of Bifidobacterium bifidum BGN4 and Bifidobacterium longum BORI Improves Cognitive and Memory Function in the Mouse Model of Alzheimer's Disease. Front Aging Neurosci. 2021;13:709091.

785. Settanni CR, Bibbo S, Ianiro G, Rinninella E, Cintoni M, Mele MC, et al. Gastrointestinal involvement of autism spectrum disorder: focus on gut microbiota. Expert Rev Gastroenterol Hepatol. 2021;15[6]:599-622.

786. Bullich C, Keshavarzian A, Garssen J, Kraneveld A, Perez-Pardo P. Gut Vibes in Parkinson's Disease: The Microbiota-Gut-Brain Axis. Mov Disord Clin Pract. 2019;6[8]:639-51.

787. Soverini M, Turroni S, Biagi E, Quercia S, Brigidi P, Candela M, et al. Variation of Carbohydrate-Active Enzyme Patterns in the Gut Microbiota of Italian Healthy Subjects and Type 2 Diabetes Patients. Front Microbiol. 2017;8:2079.

788. Conte MP, Schippa S, Zamboni I, Penta M, Chiarini F, Seganti L, et al. Gut-associated bacterial microbiota in paediatric patients with inflammatory bowel disease. Gut. 2006;55[12]:1760-7.

789. Malinen E, Rinttila T, Kajander K, Matto J, Kassinen A, Krogius L, et al. Analysis of the fecal microbiota of irritable bowel syndrome patients and healthy controls with real-time PCR. Am J Gastroenterol. 2005;100[2]:373-82.

790. Greathouse KL, White JR, Padgett RN, Perrotta BG, Jenkins GD, Chia N, et al. Gut microbiome meta-analysis reveals dysbiosis is independent of body mass index in predicting risk of obesity-associated CRC. BMJ Open Gastroenterol. 2019;6[1]:e000247.

791. Ha S, Oh D, Lee S, Park J, Ahn J, Choi S, et al. Altered Gut Microbiota in Korean Children with Autism Spectrum Disorders. Nutrients. 2021;13[10].

792. Bolliri C, Fontana A, Cereda E, Barichella M, Cilia R, Ferri V, et al. Gut Microbiota in Monozygotic Twins Discordant for Parkinson's Disease. Ann Neurol. 2022;92[4]:631-6.

793. Galley JD, Mashburn-Warren L, Blalock LC, Lauber CL, Carroll JE, Ross KM, et al. Maternal anxiety, depression and stress affects offspring gut microbiome diversity and bifidobacterial abundances. Brain Behav Immun. 2023;107:253-64.

794. Badran M, Khalyfa A, Ericsson AC, Puech C, McAdams Z, Bender SB, et al. Gut microbiota mediate vascular dysfunction in a murine model of sleep apnoea: effect of probiotics. Eur Respir J. 2023;61[1].

795. Yao S, Zhao Z, Wang W, Liu X. Bifidobacterium Longum: Protection against Inflammatory Bowel Disease. J Immunol Res. 2021;2021:8030297.

796. Fanigliulo L, Comparato G, Aragona G, Cavallaro L, Iori V, Maino M, et al. Role of gut microflora and probiotic effects in the irritable bowel syndrome. Acta Biomed. 2006;77[2]:85-9.

797. Coretti L, Paparo L, Riccio MP, Amato F, Cuomo M, Natale A, et al. Corrigendum: Gut Microbiota Features in Young Children With Autism Spectrum Disorders. Front Microbiol. 2019;10:920.

798. Tian P, Zou R, Song L, Zhang X, Jiang B, Wang G, et al. Ingestion of Bifidobacterium longum subspecies infantis strain CCFM687 regulated emotional behavior and the central BDNF pathway in chronic stress-induced depressive mice through reshaping the gut microbiota. Food Funct. 2019;10[11]:7588-98.

799. Malaguarnera M, Greco F, Barone G, Gargante MP, Malaguarnera M, Toscano MA. Bifidobacterium longum with fructo-oligosaccharide [FOS] treatment in minimal hepatic encephalopathy: a randomized, double-blind, placebo-controlled study. Dig Dis Sci. 2007;52[11]:3259-65.

800. Olson CA, Iniguez AJ, Yang GE, Fang P, Pronovost GN, Jameson KG, et al. Alterations in the gut microbiota contribute to cognitive impairment induced by the ketogenic diet and hypoxia. Cell Host Microbe. 2021;29[9]:1378-92 e6.

801. Velizarova M, Yanachkova V, Boneva T, Giragosyan S, Mihaleva I, Andreeva-Gateva P, et al. Relationship between Vitamin D status and microbiome changes in Bulgarian patients with type 2 diabetes mellitus. Biotechnology & Biotechnological Equipment. 2023;37[1]:2209662.

802. Devkota S, Chang EB. Interactions between diet, bile acid metabolism, gut microbiota, and inflammatory bowel diseases. Dig Dis. 2015;33[3]:351-6.

803. Jacobs JP, Lagishetty V, Hauer MC, Labus JS, Dong TS, Toma R, et al. Multi-omics profiles of the intestinal microbiome in irritable bowel syndrome and its bowel habit subtypes. Microbiome. 2023;11[1]:5.

804. Jian C, Luukkonen P, Sadevirta S, Yki-Jarvinen H, Salonen A. Impact of short-term overfeeding of saturated or unsaturated fat or sugars on the gut microbiota in relation to liver fat in obese and overweight adults. Clin Nutr. 2021;40[1]:207-16.

805. Verhaar BJH, Hendriksen HMA, de Leeuw FA, Doorduijn AS, van Leeuwenstijn M, Teunissen CE, et al. Gut Microbiota Composition Is Related to AD Pathology. Front Immunol. 2021;12:794519.

806. Kittana M, Ahmadani A, Al Marzooq F, Attlee A. Dietary Fat Effect on the Gut Microbiome, and Its Role in the Modulation of Gastrointestinal Disorders in Children with Autism Spectrum Disorder. Nutrients. 2021;13[11].

807. Stevens BR, Pepine CJ, Richards EM, Kim S, Raizada MK. Depressive hypertension: A proposed human endotype of brain/gut microbiome dysbiosis. Am Heart J. 2021;239:27-37.

808. Di Lodovico L, Mondot S, Dore J, Mack I, Hanachi M, Gorwood P. Anorexia nervosa and gut microbiota: A systematic review and quantitative synthesis of pooled microbiological data. Prog Neuropsychopharmacol Biol Psychiatry. 2021;106:110114.

809. Parnell JA, Reimer RA. Prebiotic fiber modulation of the gut microbiota improves risk factors for obesity and the metabolic syndrome. Gut Microbes. 2012;3[1]:29-34.

810. Zhang H, Chang Y, Zheng Q, Zhang R, Hu C, Jia W. Altered intestinal microbiota associated with colorectal cancer. Front Med. 2019;13[4]:461-70.

811. Gonzalez Cordero EM, Cuevas-Budhart MA, Perez Moran D, Trejo Villeda MA, Gomez-Del-Pulgar Gf-MM. Relationship Between the Gut Microbiota and Alzheimer's Disease: A Systematic Review. J Alzheimers Dis. 2022;87[2]:519-28.

812. Matarazzo I, Toniato E, Robuffo I. Psychobiome Feeding Mind: Polyphenolics in Depression and Anxiety. Curr Top Med Chem. 2018;18[24]:2108-15.

813. Million M, Tidjani Alou M, Khelaifia S, Bachar D, Lagier JC, Dione N, et al. Increased Gut Redox and Depletion of Anaerobic and Methanogenic Prokaryotes in Severe Acute Malnutrition. Sci Rep. 2016;6:26051.

814. Leung J, Burke B, Ford D, Garvin G, Korn C, Sulis C, et al. Possible association between obesity and Clostridium difficile infection. Emerg Infect Dis. 2013;19[11]:1791-8.

815. Jahani-Sherafat S, Azimirad M, Alebouyeh M, Ahmadi Amoli H, Hosseini P, Ghasemian-Safaei H, et al. The rate and importance of Clostridium difficile in colorectal cancer patients. Gastroenterol Hepatol Bed Bench. 2019;12[4]:358-63.

816. Toh MC, Allen-Vercoe E. The human gut microbiota with reference to autism spectrum disorder: considering the whole as more than a sum of its parts. Microb Ecol Health Dis. 2015;26[1]:26309.

817. Evrensel A, Ceylan ME. The Gut-Brain Axis: The Missing Link in Depression. Clin Psychopharmacol Neurosci. 2015;13[3]:239-44.

818. Riordan SM, Williams R. Gut flora and hepatic encephalopathy in patients with cirrhosis. N Engl J Med. 2010;362[12]:1140-2.

819. Swidsinski A, Weber J, Loening-Baucke V, Hale LP, Lochs H. Spatial organization and composition of the mucosal flora in patients with inflammatory bowel disease. J Clin Microbiol. 2005;43[7]:3380-9.

820. Maukonen J, Satokari R, Matto J, Soderlund H, Mattila-Sandholm T, Saarela M. Prevalence and temporal stability of selected clostridial groups in irritable bowel syndrome in relation to predominant faecal bacteria. J Med Microbiol. 2006;55[Pt 5]:625-33.

821. Collado MC, Isolauri E, Laitinen K, Salminen S. Distinct composition of gut microbiota during pregnancy in overweight and normal-weight women. Am J Clin Nutr. 2008;88[4]:894-9.

822. Kim E, Coelho D, Blachier F. Review of the association between meat consumption and risk of colorectal cancer. Nutr Res. 2013;33[12]:983-94.

823. Kleessen B, Kroesen AJ, Buhr HJ, Blaut M. Mucosal and invading bacteria in patients with inflammatory bowel disease compared with controls. Scand J Gastroenterol. 2002;37[9]:1034-41.

824. Palacios T, Coulson S, Butt H, Vitetta L. The gastrointestinal microbiota and multi-strain probiotic therapy: in children and adolescent obesity. Advances in Integrative Medicine. 2014;1[1]:2-8.

825. Luna RA, Oezguen N, Balderas M, Venkatachalam A, Runge JK, Versalovic J, et al. Distinct Microbiome-Neuroimmune Signatures Correlate With Functional Abdominal Pain in Children With Autism Spectrum Disorder. Cell Mol Gastroenterol Hepatol. 2017;3[2]:218-30.

826. Karnachuk OV, Ikkert OP, Avakyan MR, Knyazev YV, M NV, Zyusman VS, et al. Desulfovibrio desulfuricans AY5 Isolated from a Patient with Autism Spectrum Disorder Binds Iron in Low-Soluble Greigite and Pyrite. Microorganisms. 2021;9[12].

827. Wu S, Hammarstedt-Nordenvall L, Jangard M, Cheng L, Radu SA, Angelidou P, et al. Tonsillar Microbiota: a Cross-Sectional Study of Patients with Chronic Tonsillitis or Tonsillar Hypertrophy. Msystems. 2021;6[2]:e01302-20.

828. El-Salhy M, Casen C, Valeur J, Hausken T, Hatlebakk JG. Responses to faecal microbiota transplantation in female and male patients with irritable bowel syndrome. World J Gastroenterol. 2021;27[18]:2219-37.

829. McLeod A, Penalver Bernabe B, Xia Y, Sanchez-Flack J, Lamar M, Schiffer L, et al. Comparing the gut microbiome of obese, African American, older adults with and without mild cognitive impairment. PLoS One. 2023;18[2]:e0280211.

830. Huh JW, Kim MJ, Kim J, Lee HG, Ryoo SB, Ku JL, et al. Enterotypical Prevotella and three novel bacterial biomarkers in preoperative stool predict the clinical outcome of colorectal cancer. Microbiome. 2022;10[1]:203.

831. Bosch JA, Nieuwdorp M, Zwinderman AH, Deschasaux M, Radjabzadeh D, Kraaij R, et al. The gut microbiota and depressive symptoms across ethnic groups. Nat Commun. 2022;13[1]:7129.

832. Badran M, Mashaqi S, Gozal D. The gut microbiome as a target for adjuvant therapy in obstructive sleep apnea. Expert Opin Ther Targets. 2020;24[12]:1263-82.

833. Yu S, Ge X, Xu H, Tan B, Tian B, Shi Y, et al. Gut microbiome and mycobiome in inflammatory bowel disease patients with Clostridioides difficile infection. Front Cell Infect Microbiol. 2023;13:1129043.

834. Shin SY, Park S, Moon JM, Kim K, Kim JW, Chun J, et al. Compositional Changes in the Gut Microbiota of Responders and Non-responders to Probiotic Treatment Among Patients With Diarrhea-predominant Irritable Bowel Syndrome: A Post Hoc Analysis of a Randomized Clinical Trial. J Neurogastroenterol Motil. 2022;28[4]:642-54.

835. Ondee T, Pongpirul K, Janchot K, Kanacharoen S, Lertmongkolaksorn T, Wongsaroj L, et al. Lactiplantibacillus plantarum dfa1 Outperforms Enterococcus faecium dfa1 on Anti-Obesity in High Fat-Induced Obesity Mice Possibly through the Differences in Gut Dysbiosis Attenuation, despite the Similar Anti-Inflammatory Properties. Nutrients. 2021;14[1].

836. Kovtun AS, Averina OV, Alekseeva MG, Danilenko VN. Antibiotic resistance genes in the gut microbiota of children with autistic spectrum disorder as possible predictors of the disease. Microbial Drug Resistance. 2020;26[11]:1307-20.

837. Loguercio C, Abbiati R, Rinaldi M, Romano A, Del Vecchio Blanco C, Coltorti M. Long-term effects of Enterococcus faecium SF68 versus lactulose in the treatment of patients with cirrhosis and grade 1-2 hepatic encephalopathy. J Hepatol. 1995;23[1]:39-46.

838. Munir SS, Sert Kuniyoshi FH, Singh P, Covassin N. Is the Gut Microbiome Implicated in the Excess Risk of Hypertension Associated with Obstructive Sleep Apnea? A Contemporary Review. Antioxidants. 2023;12[4]:866.

839. Tsai H-J, Tsai W-C, Hung W-C, Hung W-W, Chang C-C, Dai C-Y, et al. Gut microbiota and subclinical cardiovascular disease in patients with type 2 diabetes mellitus. Nutrients. 2021;13[8]:2679.

840. Rhodes JM. The role of Escherichia coli in inflammatory bowel disease. Gut. 2007;56[5]:610-2.

841. Liu HN, Wu H, Chen YZ, Chen YJ, Shen XZ, Liu TT. Altered molecular signature of intestinal microbiota in irritable bowel syndrome patients compared with healthy controls: A systematic review and meta-analysis. Dig Liver Dis. 2017;49[4]:331-7.

842. Veziant J, Villeger R, Barnich N, Bonnet M. Gut Microbiota as Potential Biomarker and/or Therapeutic Target to Improve the Management of Cancer: Focus on Colibactin-Producing Escherichia coli in Colorectal Cancer. Cancers [Basel]. 2021;13[9].

843. Wu H, Wei J, Zhao X, Liu Y, Chen Z, Wei K, et al. Neuroprotective effects of an engineered Escherichia coli Nissle 1917 on Parkinson's disease in mice by delivering GLP-1 and modulating gut microbiota. Bioengineering & Translational Medicine. 2022:e10351.

844. Yun S-W, Kim J-K, Han M, Kim D-H. Lacticaseibacillus paracasei NK112 mitigates Escherichia coli-induced depression and cognitive impairment in mice by regulating IL-6 expression and gut microbiota. Beneficial microbes. 2021;12[6]:541-51.

845. Manzhalii E, Moyseyenko V, Kondratiuk V, Molochek N, Falalyeyeva T, Kobyliak N. Effect of a specific Escherichia coli Nissle 1917 strain on minimal/mild hepatic encephalopathy treatment. World J Hepatol. 2022;14[3]:634-46.

846. Morales EJDC. Obstructive Sleep Apnea and the Gut Microbiome: The University of Arizona; 2018.

847. Ge X, Zhang A, Li L, Sun Q, He J, Wu Y, et al. Application of machine learning tools: Potential and useful approach for the prediction of type 2 diabetes mellitus based on the gut microbiome profile. Exp Ther Med. 2022;23[4]:305.

848. Balamurugan R, George G, Kabeerdoss J, Hepsiba J, Chandragunasekaran AM, Ramakrishna BS. Quantitative differences in intestinal Faecalibacterium prausnitzii in obese Indian children. Br J Nutr. 2010;103[3]:335-8.

849. Bojovic K, Ignjatovic Eth I, Sokovic Bajic S, Vojnovic Milutinovic D, Tomic M, Golic N, et al. Gut Microbiota Dysbiosis Associated With Altered Production of Short Chain Fatty Acids in Children With Neurodevelopmental Disorders. Front Cell Infect Microbiol. 2020;10:223.

850. Bedarf JR, Hildebrand F, Coelho LP, Sunagawa S, Bahram M, Goeser F, et al. Erratum to: Functional implications of microbial and viral gut metagenome changes in early stage L-DOPA-naive Parkinson's disease patients. Genome Med. 2017;9[1]:61.

851. Lu Q, Lai J, Lu H, Ng C, Huang T, Zhang H, et al. Gut Microbiota in Bipolar Depression and Its Relationship to Brain Function: An Advanced Exploration. Front Psychiatry. 2019;10:784.

852. Sokol H, Pigneur B, Watterlot L, Lakhdari O, Bermudez-Humaran LG, Gratadoux JJ, et al. Faecalibacterium prausnitzii is an anti-inflammatory commensal bacterium identified by gut microbiota analysis of Crohn disease patients. Proc Natl Acad Sci U S A. 2008;105[43]:16731-6.

853. Chumpitazi BP, Cope JL, Hollister EB, Tsai CM, McMeans AR, Luna RA, et al. Randomised clinical trial: gut microbiome biomarkers are associated with clinical response to a low FODMAP diet in children with the irritable bowel syndrome. Aliment Pharmacol Ther. 2015;42[4]:418-27.

854. Ueda A, Shinkai S, Shiroma H, Taniguchi Y, Tsuchida S, Kariya T, et al. Identification of Faecalibacterium prausnitzii strains for gut microbiome-based intervention in Alzheimer's-type dementia. Cell Rep Med. 2021;2[9]:100398.

855. Unger MM, Spiegel J, Dillmann K-U, Grundmann D, Philippeit H, Bürmann J, et al. Short chain fatty acids and gut microbiota differ between patients with Parkinson's disease and age-matched controls. Parkinsonism Relat Disord. 2016;32:66-72.

856. Zhang X, Li X, Xu H, Fu Z, Wang F, Huang W, et al. Changes in the oral and nasal microbiota in pediatric obstructive sleep apnea. J Oral Microbiol. 2023;15[1]:2182571.

857. Sedighi M, Razavi S, Navab-Moghadam F, Khamseh ME, Alaei-Shahmiri F, Mehrtash A, et al. Comparison of gut microbiota in adult patients with type 2 diabetes and healthy individuals. Microb Pathog. 2017;111:362-9.

858. Lin S, Zhang X, Zhu X, Jiao J, Wu Y, Li Y, et al. Fusobacterium nucleatum aggravates ulcerative colitis through promoting gut microbiota dysbiosis and dysmetabolism. J Periodontol. 2023;94[3]:405-18.

859. Kushak RI, Sengupta A, Winter HS. Interactions between the intestinal microbiota and epigenome in individuals with autism spectrum disorder. Dev Med Child Neurol. 2022;64[3]:296-304.

860. Kountouras J, Polyzos SA, Deretzi G. Helicobacter pylori associated with obstructive sleep apnea might contribute to sleep, cognition, and driving performance disturbances in patients with cirrhosis. Clin Gastroenterol Hepatol. 2015;13[8]:1547.

861. Gupta SS, Mohammed MH, Ghosh TS, Kanungo S, Nair GB, Mande SS. Metagenome of the gut of a malnourished child. Gut Pathog. 2011;3:7.

862. Lender N, Talley NJ, Enck P, Haag S, Zipfel S, Morrison M, et al. Review article: Associations between Helicobacter pylori and obesity--an ecological study. Aliment Pharmacol Ther. 2014;40[1]:24-31.

863. Jahani-Sherafat S, Alebouyeh M, Moghim S, Ahmadi Amoli H, Ghasemian-Safaei H. Role of gut microbiota in the pathogenesis of colorectal cancer; a review article. Gastroenterol Hepatol Bed Bench. 2018;11[2]:101-9.

864. Elfil M, Kamel S, Kandil M, Koo BB, Schaefer SM. Implications of the Gut Microbiome in Parkinson's Disease. Mov Disord. 2020;35[6]:921-33.

865. Mobini R, Tremaroli V, Ståhlman M, Karlsson F, Levin M, Ljungberg M, et al. Metabolic effects of L actobacillus reuteri DSM 17938 in people with type 2 diabetes: A randomized controlled trial. Diabetes, Obesity and Metabolism. 2017;19[4]:579-89.

866. Wang H, Zhou C, Huang J, Kuai X, Shao X. The potential therapeutic role of Lactobacillus reuteri for treatment of inflammatory bowel disease. Am J Transl Res. 2020;12[5]:1569-83.

867. Niv E, Naftali T, Hallak R, Vaisman N. The efficacy of Lactobacillus reuteri ATCC 55730 in the treatment of patients with irritable bowel syndrome--a double blind, placebo-controlled, randomized study. Clin Nutr. 2005;24[6]:925-31.

868. Bell HN, Rebernick RJ, Goyert J, Singhal R, Kuljanin M, Kerk SA, et al. Reuterin in the healthy gut microbiome suppresses colorectal cancer growth through altering redox balance. Cancer Cell. 2022;40[2]:185-200 e6.

869. Liu G, Tan FH, Lau SA, Jaafar MH, Chung FY, Azzam G, et al. Lactic acid bacteria feeding reversed the malformed eye structures and ameliorated gut microbiota profiles of Drosophila melanogaster Alzheimer's disease model. J Appl Microbiol. 2022;132[4]:3155-67.

870. Sgritta M, Dooling SW, Buffington SA, Momin EN, Francis MB, Britton RA, et al. Mechanisms Underlying Microbial-Mediated Changes in Social Behavior in Mouse Models of Autism Spectrum Disorder. Neuron. 2019;101[2]:246-59 e6.

871. Du Y, Li Y, Xu X, Li R, Zhang M, Cui Y, et al. Probiotics for constipation and gut microbiota in Parkinson's disease. Parkinsonism Relat Disord. 2022;103:92-7.

872. Zhou H, Wang R, Zhang S, Zhang X, Zhou H, Wen T, et al. Depression-like symptoms due to Dcf1 deficiency are alleviated by intestinal transplantation of Lactobacillus murine and Lactobacillus reuteri. Biochem Biophys Res Commun. 2022;593:137-43.

873. Rivera-Flores R, Morán-Villota S, Cervantes-Barragán L, López-Macias C, Uribe M. Manipulation of microbiota with probiotics as an alternative for treatment of hepatic encephalopathy. Nutrition. 2020;73:110693.

874. Zhang W-Y, Zhang H-H, Yu C-H, Fang J, Ying H-Z. Ethanol extract of Atractylodis macrocephalae Rhizoma ameliorates insulin resistance and gut microbiota in type 2 diabetic db/db mice. J Funct Foods. 2017;39:139-51.

875. Ghoshal U, Shukla R, Srivastava D, Ghoshal UC. Irritable Bowel Syndrome, Particularly the Constipation-Predominant Form, Involves an Increase in Methanobrevibacter smithii, Which Is Associated with Higher Methane Production. Gut Liver. 2016;10[6]:932-8.

876. Tidjani Alou M, Million M, Traore SI, Mouelhi D, Khelaifia S, Bachar D, et al. Gut Bacteria Missing in Severe Acute Malnutrition, Can We Identify Potential Probiotics by Culturomics? Front Microbiol. 2017;8:899.

877. Liu D, Zhang S, Li S, Zhang Q, Cai Y, Li P, et al. Indoleacrylic acid produced by Parabacteroides distasonis alleviates type 2 diabetes via activation of AhR to repair intestinal barrier. BMC Biol. 2023;21[1]:90.

878. Herndon CC, Wang YP, Lu CL. Targeting the gut microbiota for the treatment of irritable bowel syndrome. Kaohsiung J Med Sci. 2020;36[3]:160-70.

879. Song H, Shen X, Chu Q, Zheng X. Pomegranate fruit pulp polyphenols reduce diet-induced obesity with modulation of gut microbiota in mice. J Sci Food Agric. 2022;102[5]:1968-77.

880. Yuan N, Li X, Wang M, Zhang Z, Qiao L, Gao Y, et al. Gut Microbiota Alteration Influences Colorectal Cancer Metastasis to the Liver by Remodeling the Liver Immune Microenvironment. Gut Liver. 2022;16[4]:575-88.

881. Averina OV, Kovtun AS, Polyakova SI, Savilova AM, Rebrikov DV, Danilenko VN. The bacterial neurometabolic signature of the gut microbiota of young children with autism spectrum disorders. J Med Microbiol. 2020;69[4]:558-71.

882. Wang L, Yu X, Xu X, Ming J, Wang Z, Gao B, et al. The fecal microbiota is already altered in normoglycemic individuals who go on to have type 2 diabetes. Frontiers in cellular and infection microbiology. 2021;11:598672.

883. Ohkusa T, Koido S. Intestinal microbiota and ulcerative colitis. J Infect Chemother. 2015;21[11]:761-8.

884. Goll R, Johnsen PH, Hjerde E, Diab J, Valle PC, Hilpusch F, et al. Effects of fecal microbiota transplantation in subjects with irritable bowel syndrome are mirrored by changes in gut microbiome. Gut Microbes. 2020;12[1]:1794263.

885. Dhopatkar N, Keeler JL, Mutwalli H, Whelan K, Treasure J, Himmerich H. Gastrointestinal symptoms, gut microbiome, probiotics and prebiotics in anorexia nervosa: A review of mechanistic rationale and clinical evidence. Psychoneuroendocrinology. 2023;147:105959.

886. Liu W, Fang X, Zhou Y, Dou L, Dou T. Machine learning-based investigation of the relationship between gut microbiome and obesity status. Microbes Infect. 2022;24[2]:104892.

887. Yoon H, Kim N, Park JH, Kim YS, Lee J, Kim HW, et al. Comparisons of Gut Microbiota Among Healthy Control, Patients With Conventional Adenoma, Sessile Serrated Adenoma, and Colorectal Cancer. J Cancer Prev. 2017;22[2]:108-14.

888. Kong X, Liu J, Cetinbas M, Sadreyev R, Koh M, Huang H, et al. New and Preliminary Evidence on Altered Oral and Gut Microbiota in Individuals with Autism Spectrum Disorder [ASD]: Implications for ASD Diagnosis and Subtyping Based on Microbial Biomarkers. Nutrients. 2019;11[9].

889. Kovtun AS, Averina OV, Angelova IY, Yunes RA, Zorkina YA, Morozova AY, et al. Alterations of the Composition and Neurometabolic Profile of Human Gut Microbiota in Major Depressive Disorder. Biomedicines. 2022;10[9].

890. Alsharairi NA. The Therapeutic Role of Short-Chain Fatty Acids Mediated Very Low-Calorie Ketogenic Diet-Gut Microbiota Relationships in Paediatric Inflammatory Bowel Diseases. Nutrients. 2022;14[19].

891. Petrov V, Alifirova V, Saltykova I, Zhukova I, Zhukova N, Dorofeeva YB, et al. Comparison study of gut microbiota in case of Parkinson’s disease and other neurological disorders. Bulletin of Siberian Medicine. 2017;15[5]:113-25.

892. Yu X, Jin Y, Zhou W, Xiao T, Wu Z, Su J, et al. Rifaximin modulates the gut microbiota to prevent hepatic encephalopathy in liver cirrhosis without impacting the Resistome. Frontiers in Cellular and Infection Microbiology. 2022;11:1427.

893. Bakir-Gungor B, Hacilar H, Jabeer A, Nalbantoglu OU, Aran O, Yousef M. Inflammatory bowel disease biomarkers of human gut microbiota selected via different feature selection methods. PeerJ. 2022;10:e13205.

894. Lyra A, Rinttila T, Nikkila J, Krogius-Kurikka L, Kajander K, Malinen E, et al. Diarrhoea-predominant irritable bowel syndrome distinguishable by 16S rRNA gene phylotype quantification. World J Gastroenterol. 2009;15[47]:5936-45.

895. Wang Y, Lim YY, He Z, Wong WT, Lai WF. Dietary phytochemicals that influence gut microbiota: Roles and actions as anti-Alzheimer agents. Crit Rev Food Sci Nutr. 2022;62[19]:5140-66.

896. Wan Y, Zuo T, Xu Z, Zhang F, Zhan H, Dorothy C, et al. Underdevelopment of the gut microbiota and bacteria species as non-invasive markers of prediction in children with autism spectrum disorder. Gut. 2022;71[5]:910-8.

897. Kwak MS, Cha JM, Shin HP, Jeon JW, Yoon JY. Development of a Novel Metagenomic Biomarker for Prediction of Upper Gastrointestinal Tract Involvement in Patients With Crohn's Disease. Front Microbiol. 2020;11:1162.

898. Hynonen U, Rasinkangas P, Satokari R, Paulin L, de Vos WM, Pietila TE, et al. Isolation and whole genome sequencing of a Ruminococcus-like bacterium, associated with irritable bowel syndrome. Anaerobe. 2016;39:60-7.

899. Togo AH, Diop A, Bittar F, Maraninchi M, Valero R, Armstrong N, et al. Correction to: Description of Mediterraneibacter massiliensis, gen. nov., sp. nov., a new genus isolated from the gut microbiota of an obese patient and reclassification of Ruminococcus faecis, Ruminococcus lactaris, Ruminococcus torques, Ruminococcus gnavus and Clostridium glycyrrhizinilyticum as Mediterraneibacter faecis comb. nov., Mediterraneibacter lactaris comb. nov., Mediterraneibacter torques comb. nov., Mediterraneibacter gnavus comb. nov. and Mediterraneibacter glycyrrhizinilyticus comb. nov. Antonie Van Leeuwenhoek. 2018;111[11]:2129-30.

900. Matto J, Maunuksela L, Kajander K, Palva A, Korpela R, Kassinen A, et al. Composition and temporal stability of gastrointestinal microbiota in irritable bowel syndrome--a longitudinal study in IBS and control subjects. FEMS Immunol Med Microbiol. 2005;43[2]:213-22.

901. Han K, Ji L, Wang C, Shao Y, Chen C, Liu L, et al. The host genetics affects gut microbiome diversity in Chinese depressed patients. Front Genet. 2022;13:976814.

902. Yukawa-Muto Y, Kamiya T, Fujii H, Mori H, Toyoda A, Sato I, et al. Distinct responsiveness to rifaximin in patients with hepatic encephalopathy depends on functional gut microbial species. Hepatol Commun. 2022;6[8]:2090-104.

903. Huang QY, Yao F, Zhou CR, Huang XY, Wang Q, Long H, et al. Role of gut microbiome in regulating the effectiveness of metformin in reducing colorectal cancer in type 2 diabetes. World J Clin Cases. 2020;8[24]:6213-28.

904. Weingarden AR, Vaughn BP. Intestinal microbiota, fecal microbiota transplantation, and inflammatory bowel disease. Gut Microbes. 2017;8[3]:238-52.

905. Beatty JK, Bhargava A, Buret AG. Post-infectious irritable bowel syndrome: mechanistic insights into chronic disturbances following enteric infection. World J Gastroenterol. 2014;20[14]:3976-85.

906. Subramanian S, Huq S, Yatsunenko T, Haque R, Mahfuz M, Alam MA, et al. Persistent gut microbiota immaturity in malnourished Bangladeshi children. Nature. 2014;510[7505]:417-21.

907. Mehal WZ. The Gordian Knot of dysbiosis, obesity and NAFLD. Nat Rev Gastroenterol Hepatol. 2013;10[11]:637-44.

908. Gao R, Gao Z, Huang L, Qin H. Gut microbiota and colorectal cancer. Eur J Clin Microbiol Infect Dis. 2017;36[5]:757-69.

909. Vojdani A, Vojdani E, Saidara E, Kharrazian D. Reaction of Amyloid-beta Peptide Antibody with Different Infectious Agents Involved in Alzheimer's Disease. J Alzheimers Dis. 2018;63[2]:847-60.

910. Mehta KK, Bhat R, Markande AR. Effects of gut bacteria and their amyloids on mental health and neurodegeneration in Parkinson’s disease. Journal of Applied Biology and Biotechnology. 2023;11[3]:38-46.

Table S2. Functionality reactions carried out by gut microbiota.

Reaction	Structural group	Examples of substrate	References
Hydrolysis	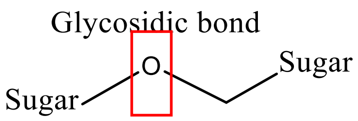	Amygdalin (i.e., plant toxin) to prunasin, to mandelonitrile and, finally, to acetonitrile and benzaldehyde.	[1, 2]
	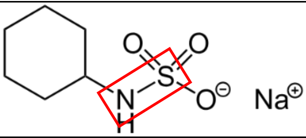	Cyclamate (i.e., artificial sweetener) to cyclohexylamine.	[3, 4]
	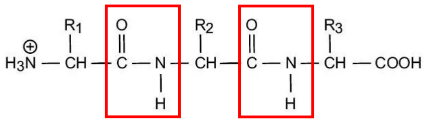	Casein and bovine serum albumin (i.e., protein) to ammonium and volatile fatty acid (e.g., acetate, propionate, butyrate, 2-methylbutyrate).	[5, 6]
Lysis	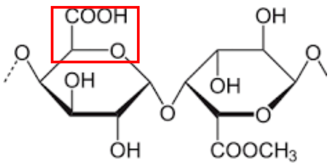	Pectin (i.e., polysaccharide) to pyruvate, then to short-chain fatty acids (e.g., acetate, butyrate, propionate) and galacturonan.	[7]
Homolysis [i.e., radical bond breaking]	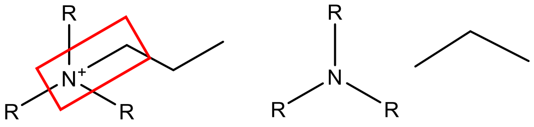	1] Choline [i.e., food nutrient] to trimethylamine and acetaldehyde.<br>2] L-tryptophan [i.e., amino acid] to products such as indole-3-propionic acid, tryptamine and indole.	[8, 9]
Reduction	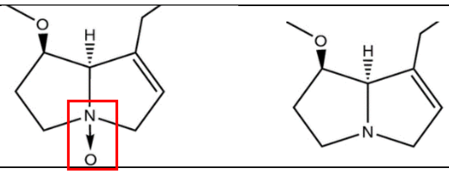	Pyrrolizidine alkaloid N-oxides [i.e., plant toxin] to parent pyrrolizidine alkaloids.	[10, 11]

	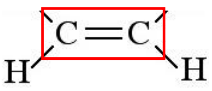 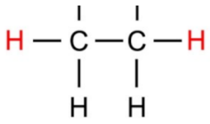	1] Cholesterol [i.e., sterol] to coprostanol.<br>2] Digoxin [i.e., heart disease drug] to 20R-dihydrodigoxin.	[12, 13]
	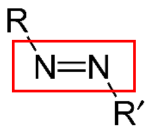 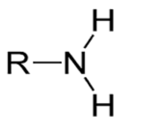	1] Azo dyes to aniline and para amino phenol.<br>2] Prontosil [i.e., antibacterial drug] to sulphanilamide and benzene-1,2,4-triamine.<br>3] Sulfasalazine [i.e., drug for IBD] to sulfapyridine [SP] and 5-acetylsalycylic acid [5-ASA].	[14-16]
	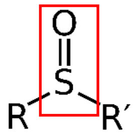 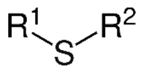	1] Sulindac [i.e., nonsteroidal anti-inflammatory prodrug with sulfoxide functional group] to sulindac sulfide.<br>2] Sulphinpyrazone [i.e., gout drug] to sulphinpyrazone sulfide.	[17]
	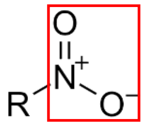 $R{-}NH_2$	1] Chloramphenicol [i.e., eye infection drug] to amino chloramphenicol.<br>2] Nitrobenzodiazepines [i.e., nitrazepam or seizure drug] to 7-amino-benzodiazepine.<br>3] Berberine [i.e., plant-based supplement] to dihydroberberine.	[18-20]
	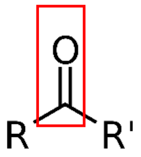 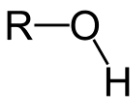	1] Nabumetonen [i.e., nonsteroidal anti-inflammatory drug] to 6-methoxy-2-naphthylacetic acid [6-MNA].<br>2] Doxorubicin [i.e., cancer chemotherapy drug] to 7-deoxydoxorubicinol and 7-deoxydoxorubicinolone.<br>3] Zearalenone [i.e., mycotoxin] to α-ZEL and β-ZEL.	[21-23]
Conjugation	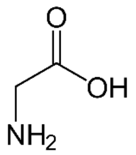 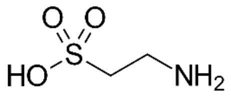 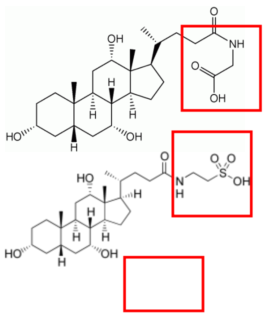	1] Cholic acid [i.e., primary bile acid] can be conjugated with either glycine or taurine into glycocholic acid and taurocholic acid, respectively.<br>2] Deoxycholic acid [i.e., secondary bile acid] with	[24]

	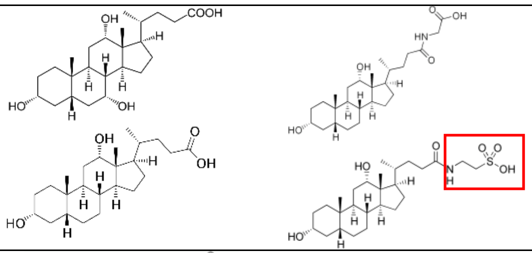	glycine and taurine into glycodeoxycholic acid and taurodeoxycholic acid.	
Deconjugation	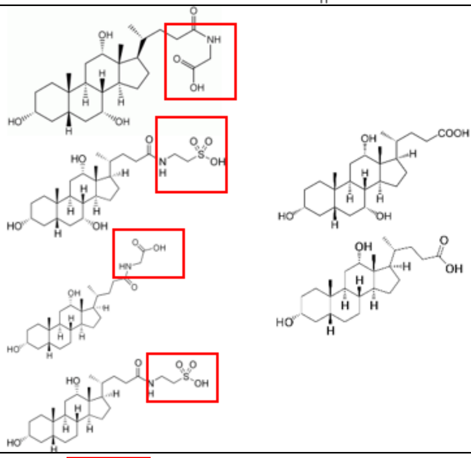	1] Glycocholic acid and taurocholic acid are deconjugated into cholic acid.<br>2] Glycodeoxycholic acid and taurodeoxycholic acid are deconjugated into deoxycholic acid.	[24]
Acetylation	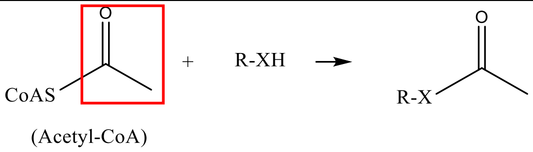	5-aminosalicylic acid (i.e., mesalamine, 5-ASA or IBD drug) to N-acetyl ASA.	[25]
Deacetylation	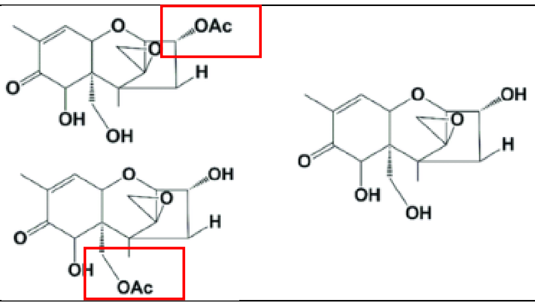	3-acetyl- deoxynivalenol and 15-acetyl-deoxynivalenol to deoxynivalenol [i.e., mycotoxin].	[26]
Deglucuronidation	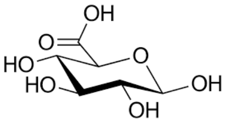 Glucuronic acid	2-amino-3-methylimidazo (4,5-*f*)-quinoline-N-glucuronide (IQ-N-glucuronide) to IQ.	[27]
Decarboxylation	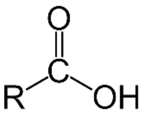 Carboxylic acid	1) p-hydroxyphenylacetate (i.e., tyrosine derived metabolite) to p-cresol.<br>2) Ellagic acid (i.e., plant secondary metabolites with antioxidant activities) to urolithins.<br>3) L-DOPA(i.e., amino acid) to dopamine.	[28-30]
Dehydroxylation	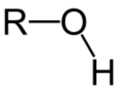 Hydroxyl group	1) L-DOPA to m-tyramine or to hydroxyphenylacetic acid.<br>2) Secoisolariciresinol diglucoside (SDG, i.e., dietary lignan) to secoisolariciresinol (SECO), to 2,3-bis(3,4-dihydroxybenzyl)butene-1,4-diol, to enterodiol	[31-33]

		(ED), to enterolactone (EL).<br>3) Gallate esters and catechins to metabolites such as gallic acid.	
Deamination	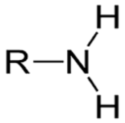<br>Amine functional group	Flucytosine (i.e., antifungals) to fluorouracil.	[34]
Ring opening	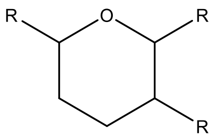 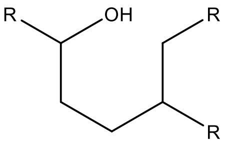	1] Epigallocatechin gallate [EGCG] and a series of downstream reactions to ring-opened metabolites.<br>2] Polyphenols ring opening to metabolites.	[35, 36]
Substitution	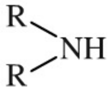 to 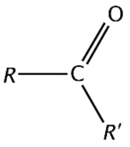	Melamine [i.e., chemical for industrial usage] to ammonia and cyanuric acid.	[37]
Decomposition	n.a.	Xylitol and other sugars to short-chain fatty acids and CO_2.	[38]

References

1. Strugala GJ, Rauws AG, Elbers R. Intestinal first pass metabolism of amygdalin in the rat in vitro. Biochem Pharmacol. 1986;35[13]:2123-8.
2. Manhardt MA. Correlation of injected amygdalin and urinary thiocyanate in JAX C57 BL/KsJ mice. 1981.
3. Niimura T, TOKIEDA T, YAMAHA T. Partial purification and some properties of cyclamate sulfamatase. The Journal of Biochemistry. 1974;75[2]:407-17.
4. Koppel N, Maini Rekdal V, Balskus EP. Chemical transformation of xenobiotics by the human gut microbiota. Science. 2017;356[6344]:eaag2770.
5. Macfarlane G, Allison C. Utilisation of protein by human gut bacteria. FEMS Microbiol Ecol. 1986;2[1]:19-24.
6. Macfarlane G, Cummings J, Allison C. Protein degradation by human intestinal bacteria. Microbiology. 1986;132[6]:1647-56.
7. Elshahed MS, Miron A, Aprotosoaie AC, Farag MA. Pectin in diet: Interactions with the human microbiome, role in gut homeostasis, and nutrient-drug interactions. Carbohydr Polym. 2021;255:117388.
8. Romano KA, Vivas EI, Amador-Noguez D, Rey FE. Intestinal microbiota composition modulates choline bioavailability from diet and accumulation of the proatherogenic metabolite trimethylamine-N-oxide. MBio. 2015;6[2]:e02481-14.
9. Williams BB, Van Benschoten AH, Cimermancic P, Donia MS, Zimmermann M, Taketani M, et al. Discovery and characterization of gut microbiota decarboxylases that can produce the neurotransmitter tryptamine. Cell Host Microbe. 2014;16[4]:495-503.
10. Yang M, Ma J, Ruan J, Ye Y, Fu PP-C, Lin G. Intestinal and hepatic biotransformation of pyrrolizidine alkaloid N-oxides to toxic pyrrolizidine alkaloids. Arch Toxicol. 2019;93[8]:2197-209.
11. Widjaja F, Wesseling S, Rietjens IMCM. Physiologically based kinetic modelling predicts the in vivo relative potency of riddelliine N-oxide compared to riddelliine in rat to be dose dependent. Arch Toxicol. 2022;96[1]:135-51.
12. Juste C, Gérard P. Cholesterol-to-coprostanol conversion by the gut microbiota: what we know, suspect, and ignore. Microorganisms. 2021;9[9]:1881.
13. Robertson L, Chandrasekaran A, Reuning R, Hui J, Rawal B. Reduction of digoxin to 20R-dihydrodigoxin by cultures of Eubacterium lentum. Appl Environ Microbiol. 1986;51[6]:1300-3.
14. Xu H, Heinze TM, Chen S, Cerniglia CE, Chen H. Anaerobic metabolism of 1-amino-2-naphthol-based azo dyes [Sudan dyes] by human intestinal microflora. Appl Environ Microbiol. 2007;73[23]:7759-62.
15. Gingell R, Bridges J, Williams R. The role of the gut flora in the metabolism of prontosil and neoprontosil in the rat. Xenobiotica. 1971;1[2]:143-56.
16. Klotz U, Maier K, Fischer C, Heinkel K. Therapeutic efficacy of sulfasalazine and its metabolites in patients with ulcerative colitis and Crohn's disease. N Engl J Med. 1980;303[26]:1499-502.
17. Strong H, Renwick A, George C, Liu Y, Hill M. The reduction of sulphinpyrazone and sulindac by intestinal bacteria. Xenobiotica. 1987;17[6]:685-96.
18. Xin Z, Fengwei T, Gang W, Xiaoming L, Qiuxiang Z, Hao Z, et al. Isolation, identification and characterization of human intestinal bacteria with the ability to utilize chloramphenicol as the sole source of carbon and energy. FEMS Microbiol Ecol. 2012;82[3]:703-12.
19. LinWu S-W, Syu C-J, Chen Y-L, Wang AH-J, Peng F-C. Characterization of Escherichia coli nitroreductase NfsB in the metabolism of nitrobenzodiazepines. Biochem Pharmacol. 2009;78[1]:96-103.
20. Feng R, Shou J-W, Zhao Z-X, He C-Y, Ma C, Huang M, et al. Transforming berberine into its intestine-absorbable form by the gut microbiota. Sci Rep. 2015;5[1]:12155.

21. Jourova L, Anzenbacher P, Matuskova Z, Vecera R, Strojil J, Kolar M, et al. Gut microbiota metabolizes nabumetone in vitro: Consequences for its bioavailability in vivo in the rodents with altered gut microbiome. Xenobiotica. 2019.
22. Yan A, Culp E, Perry J, Lau JT, MacNeil LT, Surette MG, et al. Transformation of the anticancer drug doxorubicin in the human gut microbiome. ACS infectious diseases. 2018;4[1]:68-76.
23. Mendez-Catala DM, Spenkelink A, Rietjens IM, Beekmann K. An in vitromodel to quantify interspecies differences in kinetics for intestinal microbial bioactivation and detoxification of zearalenone. Toxicology Reports. 2020;7:938-46.
24. Bachmann V, Kostiuk B, Unterweger D, Diaz-Satizabal L, Ogg S, Pukatzki S. Bile salts modulate the mucin-activated type VI secretion system of pandemic Vibrio cholerae. PLoS Negl Trop Dis. 2015;9[8]:e0004031.
25. Deloménie C, Fouix S, Longuemaux S, Brahimi Nm, Bizet C, Picard B, et al. Identification and functional characterization of arylamine N-acetyltransferases in eubacteria: evidence for highly selective acetylation of 5-aminosalicylic acid. J Bacteriol. 2001;183[11]:3417-27.
26. Li F, Jin J, Rietjens IM, Xing F. Interindividual differences in in vitro human intestinal microbial conversion of 3-acetyl-DON and 15-acetyl-DON. Toxins [Basel]. 2022;14[3]:199.
27. Humblot C, Murkovic M, Rigottier-Gois L, Bensaada M, Bouclet A, Andrieux C, et al. β-glucuronidase in human intestinal microbiota is necessary for the colonic genotoxicity of the food-borne carcinogen 2-amino-3-methylimidazo [4, 5-f] quinoline in rats. Carcinogenesis. 2007;28[11]:2419-25.
28. van Kessel SP, Frye AK, El-Gendy AO, Castejon M, Keshavarzian A, van Dijk G, et al. Gut bacterial tyrosine decarboxylases restrict levels of levodopa in the treatment of Parkinson's disease. Nature communications. 2019;10[1]:310.
29. Saito Y, Sato T, Nomoto K, Tsuji H. Identification of phenol-and p-cresol-producing intestinal bacteria by using media supplemented with tyrosine and its metabolites. FEMS Microbiol Ecol. 2018;94[9]:fiy125.
30. García-Villalba R, Beltrán D, Espín JC, Selma MV, Tomás-Barberán FA. Time course production of urolithins from ellagic acid by human gut microbiota. J Agric Food Chem. 2013;61[37]:8797-806.
31. GOLDIN BR, PEPPERCORN MA, GOLDMAN P. Contributions of host and intestinal microflora in the metabolism of L-dopa by the rat. J Pharmacol Exp Ther. 1973;186[1]:160-6.
32. Clavel T, Henderson G, Engst W, Dore J, Blaut M. Phylogeny of human intestinal bacteria that activate the dietary lignan secoisolariciresinol diglucoside. FEMS Microbiol Ecol. 2006;55[3]:471-8.
33. Zhang S, Mao B, Cui S, Zhang Q, Zhao J, Tang X, et al. Absorption, metabolism, bioactivity, and biotransformation of epigallocatechin gallate. Crit Rev Food Sci Nutr. 2023:1-21.
34. Malet-Martino M-C, Martino R, De Forni M, Andremont A, Hartmann O, Armand J. Flucytosine conversion to fluorouracil in humans: does a correlation with gut flora status exist? A report of two cases using fluorine-19 magnetic resonance spectroscopy. Infection. 1991;19[3]:178-80.
35. Liu Z, de Bruijn WJ, Bruins ME, Vincken J-P. Reciprocal interactions between epigallocatechin-3-gallate [EGCG] and human gut microbiota in vitro. J Agric Food Chem. 2020;68[36]:9804-15.
36. Espín JC, González-Sarrías A, Tomás-Barberán FA. The gut microbiota: A key factor in the therapeutic effects of [poly] phenols. Biochem Pharmacol. 2017;139:82-93.
37. Zheng X, Zhao A, Xie G, Chi Y, Zhao L, Li H, et al. Melamine-induced renal toxicity is mediated by the gut microbiota. Sci Transl Med. 2013;5[172]:172ra22-ra22.
38. Oku T, Nakamura S. Digestion, absorption, fermentation, and metabolism of functional sugar substitutes and their available energy. Pure Appl Chem. 2002;74[7]:1253-61.
